# Reframing the Biological Basis of Neuroprotection Using Functional Genomics: Differentially Weighted, Time-Dependent Multifactor Pathogenesis of Human Ischemic Brain Damage

**DOI:** 10.3389/fneur.2018.00497

**Published:** 2018-06-26

**Authors:** William A. Kofke, Yue Ren, John G. Augoustides, Hongzhe Li, Katherine Nathanson, Robert Siman, Qing Cheng Meng, Weiming Bu, Sukanya Yandrawatthana, Guy Kositratna, Cecilia Kim, Joseph E. Bavaria

**Affiliations:** ^1^Department of Anesthesiology and Critical Care, University of Pennsylvania, Philadelphia, PA, United States; ^2^Department of Biostatistics and Epidemiology, University of Pennsylvania, Philadelphia, PA, United States; ^3^Department of Medicine, Division of Translational Medicine and Human Genetics Abramson Cancer Center Perelman School of Medicine at the University of Pennsylvania, Philadelphia, PA, United States; ^4^Department of Neurosurgery, University of Pennsylvania, Philadelphia, PA, United States; ^5^The Center for Applied Genomics, Children's Hospital of Philadelphia, Philadelphia, PA, United States; ^6^Department of Surgery, University of Pennsylvania, Philadelphia, PA, United States

**Keywords:** neuroprotection, functional genomics, cerebral ischemia, cardiac surgery, cardiopulmonary bypass, biomarkers, systems biology, clinical trials

## Abstract

**Background:** Neuroprotection studies are generally unable to demonstrate efficacy in humans. Our specific hypothesis is that multiple pathophysiologic pathways, of variable importance, contribute to ischemic brain damage. As a corollary to this, we discuss the broad hypothesis that a multifaceted approach will improve the probability of efficacious neuroprotection. But to properly test this hypothesis the nature and importance of the multiple contributing pathways needs elucidation. Our aim is to demonstrate, using functional genomics, in human cardiac surgery procedures associated with cerebral ischemia, that the pathogenesis of perioperative human ischemic brain damage involves the function of multiple variably weighted proteins involving several pathways. We then use these data and literature to develop a proposal for rational design of human neuroprotection protocols.

**Methods:** Ninety-four patients undergoing deep hypothermic circulatory arrest (DHCA) and/or aortic valve replacement surgery had brain damage biomarkers, S100β and neurofilament H (NFH), assessed at baseline, 1 and 24 h post-cardiopulmonary bypass (CPB) with analysis for association with 92 single nucleotide polymorphisms (SNPs) (selected by co-author WAK) related to important proteins involved in pathogenesis of cerebral ischemia.

**Results:** At the nominal significance level of 0.05, changes in S100β and in NFH at 1 and 24 h post-CPB were associated with multiple SNPs involving several prospectively determined pathophysiologic pathways, but were not individually significant after multiple comparison adjustments. Variable weights for the several evaluated SNPs are apparent on regression analysis and, notably, are dissimilar related to the two biomarkers and over time post CPB. Based on our step-wise regression model, at 1 h post-CPB, SOD2, SUMO4, and GP6 are related to relative change of NFH while TNF, CAPN10, NPPB, and SERPINE1 are related to the relative change of S100B. At 24 h post-CPB, ADRA2A, SELE, and BAX are related to the relative change of NFH while SLC4A7, HSPA1B, and FGA are related to S100B.

**Conclusions:** In support of the proposed hypothesis, association SNP data suggest function of specific disparate proteins, as reflected by genetic variation, may be more important than others with variation at different post-insult times after human brain ischemia. Such information may support rational design of post-insult time-sensitive multifaceted neuroprotective therapies.

## Introduction

Donnan (1) in the 2007 Feinberg lecture made this remarkable statement about neuroprotection research:

**“*We have reached a stage at which research in this area should stop altogether or*** ***radical new approaches adopted*.**”

Donnan's challenge was to develop a radical, transformative, new approach to studying neuroprotection. However, little has changed since 2007 in the approach to clinical neuroprotection studies other than efforts to do a better job with the process of monotherapy preclinical research (2, 3). Over 23,000 publications can be found dealing with stroke and its treatment in various preclinical models. Over 4,700 clinical trials with over 6,300 interventions are listed on the Internet Stroke Trials Registry (4) based on this extensive volume of preclinical work. However, unfortunately there are few apparent reproducible and fully implemented results of any demonstrable efficacy in the acute context in humans. This constitutes a massive failure to translate preclinical findings to humans. Various causes of this futility in neuroprotection research have been suggested (5–14). These include the use of animal models lacking the concomitant anatomy and diseases seen in humans, overly homogeneous animal model insults, inbred animal strains without co-morbid diseases, timing and dosing of therapy in relation to ischemia (before, during, after), the problematic use of monotherapy for a pathophysiologically multifaceted disease (5, 8, 11, 13, 15–17), and variations in the health care system with varying approaches to overall care (16, 18–25).

Several high level commissions were tasked to solve this problem. This has resulted in the STAIR (3) and ARRIVE (26) recommendations for proper standards in the conduct and transparent reporting of preclinical neuroprotective drug development (Notably the STAIR guidelines do not discount so-called “cocktail” combination therapies). The International Mission on Prognosis and Clinical Trial Design (IMPACT) (27–29) was also assembled to evaluate comparable issues in traumatic brain injury neuroprotection trials. In addition, the NINDS sponsored a workshop on Improving the Quality of NINDS-Supported Preclinical and Clinical Research through Rigorous Study Design and Transparent Reporting was held in 2012 (30) with promulgation of the RIGOR guidelines (31). In addition the Multicentre Preclinical Animal Research Team (Multi-Part) (32) provides a detailed prescription for the conduct of multi-institutional randomized pre-clinical trials. Others provide support for this notion (33, 34) and have also written reviews and editorials urging better quality preclinical research in support of the concepts espoused in the STAIR and ARRIVE guidelines (35–37). It is notable that all of these efforts to improve the translation of preclinical research generally entail simply improving existing approaches without truly innovative approaches to the problem, which we believe we are herein suggesting.

Neuroprotection, since Donnan's comments in 2007, notwithstanding the aforementioned several commissions (STAIR, ARRIVE, IMPACT, Multi-Part) addressing the problem, continues as a major challenge, and opportunity, in clinical medicine. The massive failure to translate preclinical findings to humans is the essence of the challenge facing us. Presently, it is apparent that the new transformative approach suggested as needed by Donnan has not yet been developed. Of the numerous reasons for the many failed human neuroprotection studies that have been suggested, one important contributor is certainly the notion that many time-dependent pathophysiologic processes are undoubtedly involved in the final outcome of a cerebral insult (16). Notably, these pathways likely interact (38), sometimes merging into so called “hub” pathways (39), and their import likely varies in relation to each other and over time after the ischemic insult (40). Thus, it follows that using a therapy oriented to a single or a few molecules or pathways of unknown relative importance and with no consideration of temporal changes in importance after an ischemic insult is problematic (16). This report explores an alternate way to think about neuroprotection…a new frame… taking into consideration multiple pathways and their relative varying time-dependent importance, which may support development of a transformative strategy to more reliably achieve human neuroprotection.

Following Donnan's charge, a potentially radical new approach would be to implement multifaceted therapy to address the multiple pathways involved in the pathogenesis of ischemic brain injury (16). Notably, the efficacious use of combination therapy is already employed in management of hypertension (41), cancer (42, 43), coronary artery disease (44), AIDS (45), and even postoperative nausea (46). However, this approach has not penetrated in a rational manner into neuroprotection. Given the time-related complexity of acute cerebral ischemia, in order to do that rationally, one needs to have information in humans of the post-ischemia time-related various pathways and their relative importance which leads to damage. The purpose of this Hypothesis and Theory report is two-fold:

1. To test the hypothesis, in humans after cardiopulmonary bypass, using functional genomics, that multiple biochemical pathways already known to be involved in ischemic brain damage have disparate importance which varies with time post-insult; and
2. Use our observations as the basis for a proposal to reframe the manner in which neuroprotection research is developed, designed, and translated.

The data presented and associated hypothesis and theory delineated is an answer to Donnan's challenge to develop a radical, *transformative*, new approach to studying and delivering neuroprotection in humans.

## Materials and methods

The protocol was approved by the institutional biomedical review board of the University of Pennsylvania. After obtaining written informed consent, 98 patients were enrolled. Four non-Caucasian patients were not included in the analysis leaving 94 patients for downstream data analysis. Patients undergoing aortic surgery with deep hypothermic circulatory arrest (DHCA) or aortic valve replacement (AVR) surgery were enrolled. Each had blood drawn for biomarkers of brain damage, S100β (47) and neurofilament H (NFH) (48, 49), immediately after induction of anesthesia (baseline-BL), just prior to CPB (aCPB), 1 h after the end of CPB (pCPB), and 24 h (24H) postoperatively. The group of patients we studied, undergoing cardiac surgery with cardiopulmonary bypass, constitute relatively homogeneous and reproducible clinical situations associated with subtle focal and global ischemic neurologic injury (50).

Each patient underwent analysis for status of 92 single nucleotide polymorphisms (SNPs) thought to be related to proteins and pathways described in the pathogenesis of ischemic brain damage. This approach was employed to avoid the need for an extremely large sample size that would be required with a more standard Genome Wide Association Study. The SNPs chosen were based on a literature review of pathogenesis of ischemic brain damage followed by determination of presence of SNPs related to relevant proteins identified from this literature search. SNPs had to have been demonstrated to have biological impact (not necessarily neurologic) on a human phenotype, and have a distribution of base-pair incidences which support valid statistical inference.

Candidate SNP's proteins include those with roles in: lipoprotein metabolism; nucleotide metabolism; vascular regulation; inflammation; protein chaperone/repair; peroxidation; calcium regulation; 2nd messenger/cell signaling; energy metabolism; platelets/coagulation; apoptotic factors; neurotransmitters; acid-base/cell volume regulation; and estrogen. The candidate proteins and the specific associated SNPs (and bibliographic citations) are fully detailed in the Supplementary Material File (Table I) and summarized in Table 1.

**Table 1:** SNPs evaluated; according to ischemic axes.

**Ischemic axis**	SNP Symbols^*^
Lipoproteins	*APOE, OLR1, LPL, MMP3*
Nucleotide metabolism	*MTHFR*
Vascular regulation	*NOS3, REN, AGT, AGTR1, CYP11B2, ANGPT1, FLT1, EDN1, ADRA1A, ADRB2, TH*
Inflammation	*IL6R, IL2, IL11, SELP, SELE, SERPINE1, TNF, IL10, TGFB1, TGFB1, NFKB1, SUMO4, THBS4, TLR2, TLR4, TLR5*
Protein chaperone/Repair	*EPO, HSPA1B, HSPA1L*
Peroxidation	*MPO, GPX1, SOD2, CAT, NOX4, MTNR1B*
Calcium regulation	*CACNA1C, SLC8A1, CACNA2D2, PLCE1, CALM2, RYR3*
2nd messenger/cell signaling	*GNB3, NOS2, ROCK2, NPPA, NPPB, NPR3, CDH1*
Energy metabolism	*UCP2, UCP3, POLG, SLC2A1, IRS1, CYP11B2*
Platelets and coagulation	*F2 (2 SNPs), GP6, ITGA2, GP1BA, FGA, FGB, MCF2L*
Apoptotic factors	*BAX, CASP10, CASP8, MDM2, TP53, CAPN10*
Neurotransmitters	*GRIA1, GRM3, GRIK2, GCOM1, SLC1A2, OPRM1, OPRD1, GABRG2, SLC18A2, ADRA2A, ADRA2C, ADRB1*
Acid-Base/Cell volume	*SLC4A7, KCNJ10, AQP4*
Estrogen	*ESR1, ESR2*
Other	*NOTCH3*

Nomenclature derived from the dbSNP database of the National Center for Biotechnology Information; http://www.ncbi.nlm.nih.gov/projects/SNP/.

* *SNP gene names and rs- ID numbers and supporting literature citations are in the Supporting Information File S1 (Table I)*.

### Surgery and anesthesia

The DHCA protocol used at the University of Pennsylvania has been described (51). Briefly, patients undergo balanced general endotracheal anesthesia with direct intra-arterial blood pressure monitoring and cardiac output monitoring via an oximetric pulmonary arterial catheter (Baxter/Edwards, Deerfield, IL). Temperature is continuously measured in the nasopharynx and bladder. For DHCA patients retrograde cerebral perfusion is initiated via superior vena cava cannula with its tip cephalad to the azygos vein and continued for the duration of DHCA at 10°C with perfusion pressure 25 mmHg, flow 200–300 mL/min and 10° Trendelenburg position. DHCA is < 1 h, after which withdrawal of cardiopulmonary bypass is effected. Cardiotomy suction is routinely returned to the cardiopulmonary bypass circuit and hence to the systemic circulation. AVR patients were similarly managed with hypothermic cardiopulmonary bypass to 30–34°C but without retrograde cerebral perfusion.

### Biomarkers

#### S100β

Samples were assayed for S100β by radioimmunoassay using human S100β ELISA kits obtained from EMD Millipore Corp. (Billerica, MA, USA). The detection limit of S100β is 2.7 pg/mL using a 50 μL sample size. The sensitivity was determined by plotting the standard curve and then measuring the point of the curve at a distance of three SD from the standard. The isoform S100β is the 21,000 Dalton homodimer ββ. It is present in high concentration in glial cells and Schwann cells (52). The S100 test kit is specific for the β-subunit of the S100 protein, and it measures the β-subunit concentration in both the ββ and αβ isoforms of the protein.

### Hypophosphorylated neurofilament H (NFH)

NFH was quantified from human sera using fluorescence sandwich ELISAs developed, described, and validated by coauthor Siman (48). For the sensitive and specific measurement of our target protein analytes in protein-rich and highly complex serum samples, we employ fluorescence-based sandwich immunoassays (ELIFAs), in which alkaline phosphatase is the reporter enzyme and 4-methylumbelliferyl-phosphate is the fluorogenic substrate. This approach increases sensitivity over standard colorimetric ELISA by at least an order of magnitude (53). For the described studies, we measured serum levels of pNFH by ELIFA. We used a modified version of a well-established immunoassay to measure a hypophosphorylated form of the high molecular weight neurofilament subunit. For sensitive and specific detection of pNFH, separate mouse and rabbit antibodies were used for the capture and detection of a hypophosphorylated form of the protein.

The ELIFAs are standardized across experiments so that treatment effects on the relative amounts of each biomarker may be compared directly. For pNFH we employed as standard a mouse spinal cord extract. Fluorescence signals from serially diluted standard samples were fit by nonlinear regression using Graph Pad Prism to create standard curves. In all cases, the fluorescence signal for the serum protein is represented as the relative fluorescence units per unit volume of standard, which in turn is normalized to the volume of input serum. Negative controls were tested to ensure the fidelity of each ELIFA by systematic deletion of either the capture reagent, antigen, or detecting antibody.

### Single nucleotide polymorphism (SNP) genotyping methods

Custom targeted SNP assays, designed in collaboration with Illumina technical service, based on the Illumina GoldenGate system were used. The GoldenGate custom multiplex platform genotypes between 96 and 1536 SNPs (in increments of 96) using primer extension and ligation reactions (54, 55). The design process, resulted in the customized list of analyzed SNPs, which were incorporated into the custom GoldenGate reagent kit. Call rates are typically over 99%, with very high reproducibility and accuracy.

### Statistical analysis

#### Population stratification

Due to the small number of African-American and Asian patients, our analysis focused only on Caucasians.

Linear regression was used to detect SNPs that are differentially associated with changes in NFH or S100β values. The model takes the form:

$$\begin{array}{l} (Y_{aCPB}-Y_{BL})/Y_{BL}=c_{0}+c_{1}\cdot Age+c_{2}\cdot Gender\\ \;+\;c_{3}\cdot First\;Glucose\;in\;ICU+c_{4}\cdot DHCA\\ \;+\;c_{5}\cdot SNP\ldots \end{array}$$

where *Y*_*aCPB*_ stands for the value of NFH or S100B at aCPB, *Y*_*BL*_ stands for the value of NFH or S100B at baseline. Similar forms of the equation were used for times pCPB and 24 H.

Bonferroni *p*-values for Studentized residuals were calculated based on t-distribution for each observation. If the *p* < 0.05, this observation was considered as an outlier and was deleted. Benjamini-Hochberg procedure was used for the multiple comparison problem.

GEE model was used to analyze the effect between different treatment groups and the time effect for S100β and NFH. In this study, observations of the sample patients were correlated with each other while those from different patients were assumed to be independent. GEE model can take into account the dependency of within group observations by specifying a working correlation structure.

Akaike information criterion (AIC) based backward stepwise regression was used to select a group of differentially associated SNPs. The model starts with a list of candidate variables which includes significant SNPs (raw *p* < 0.05) from previous linear regression result and other covariates. At each step, AIC, which measures the information lost for the model, is calculated. If deleting a variable results in a lower AIC, then the variable is dropped. Otherwise, it is kept in the model. Similar outlier detection methods are applied and the outliers are removed in the final model.

## Results

Ninety-four enrolled patients underwent analysis. Eleven underwent DHCA only, 65 underwent AVR only with hypothermic cardiopulmonary bypass, and 18 underwent both procedures. Patient characteristics are summarized in Table 2.

**Table 2:** Patient characteristics.

**Ethnicity**	**N**	**Age (Yr)**		**Date of birth**	
Caucasian	94	24–59	28	1900–1935	26
		60–69	26	1936–1945	23
		70–79	25	1946–1955	22
		80–88	15	1956–1988	23
**Gender**		**Education completed**		**Surgery**	
Male	72	Under 8th grade	94	AVR	65
Female	22	8–12 grade	94	DHCA	11
		College	62	AVR & DHCA	18
		Post graduate	8		

*Data listed are numbers of patients in each category*.

**Figure 1:**
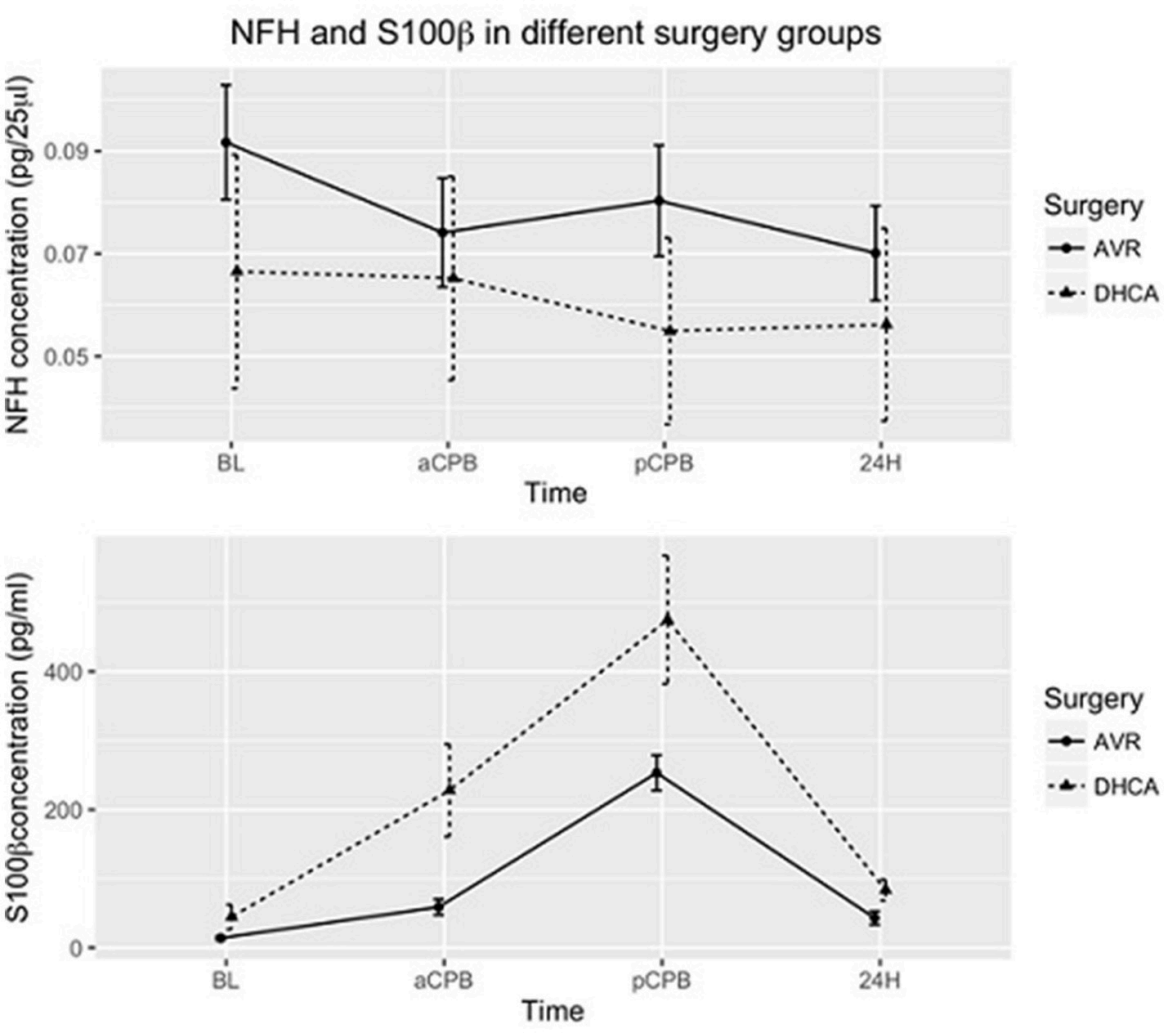
Mean of NFH and S100β concentrations in patients undergoing DHCA (with or without AVR) and patients undergoing AVR only. The error bars show the mean ± standard error. Group differences for S100 β are significant (*P* = 0.0014). In addition, S100β concentrations change significantly over time (*P* < 2 × 10^−16^). Group assignments were: immediately after induction of anesthesia (baseline-BL), just prior to CPB (aCPB), 1 h after the end of CPB (pCPB), and 24 h (24 H) postoperatively.

The result of the GEE model shows that the surgical group difference of S100β concentration between DHCA patients and AVR-only patients is significant (*P* = 0.0014) while no group differences are apparent for NFH concentration (*P* = 0.78; Figure 1) NFH concentration has a large variability in both surgery groups and the error bars have overlap. As for the S100β concentrations, patients undergoing DHCA procedure have higher S100β concentrations than those undergoing only AVR surgery. Moreover, S100β concentration changes significantly (*P* < 2 × 10^−16^) over time.

**Figure 2:**
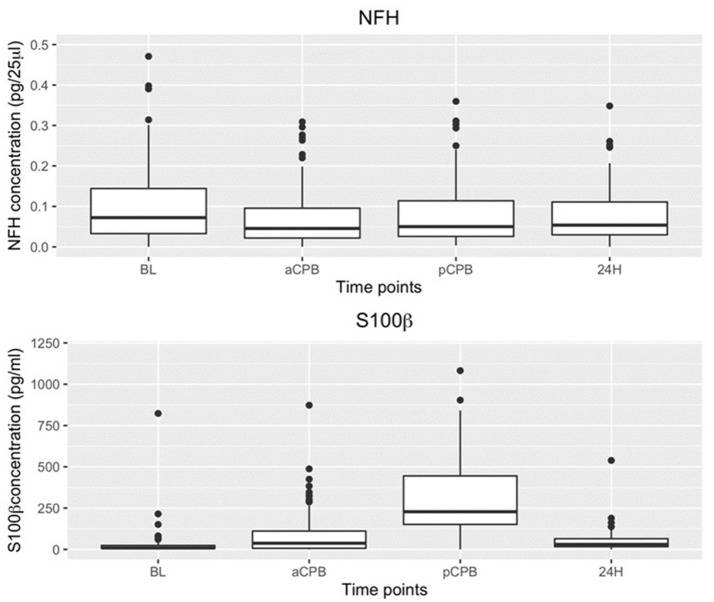
Boxplot of NFH and S100β concentrations at baseline (BL) before (aCPB) and after (pCPB)CPB and 24 h post CPB(24 h) for AVR and DHCA patients combined. Compared to BL NFH values were not increased before or after CPB. S100β increased from baseline before (aCPB) (*P* = 2.99 × 10^−6^) and after (pCPB) (*P* = 3.33 × 10^−13^) CPB but not at 24 h.

In the combined group analysis, S100β values compared to baseline were elevated before (*P* = 2.99 × 10^−6^) and after (*P* = 3.33 × 10^−13^) CPB but not at 24 h (*P* = 0.136). NFH values overall were minimally changed from baseline (Figure 2).

Genotyping call scores were acceptable in 85 subjects. Among these patients the median call score was 1.0 with IQR 0.989–1.0 and range 0.213–1.0; and mean call score was 0.926 ± 0.194 (SD).

At the nominal significance level of *P* = 0.05, absolute levels of S100β and NFH and relative changes in S100β and NFH compared to baseline were associated with SNPs in multiple genes involving several pathways with different patterns over time. However, the associations were not significant after multiple comparison adjustments. These nominally significant associations are listed in Table 3 (presented without multiple comparison considerations in order to demonstrate the multiplicity of involved ischemic axes and support future specific SNP and pathway hypothesis testing). Among these SNPs for which a nominal association with biomarkers was present, specific base pair combinations related to elevations in biomarker levels were observed. Figures depicting these can be found in the Supplementary Material File (Figure I).

**Table 3:** Nominally significant SNP associations for relative changes in biomarkers.

**Pathway**	**SNP**	**Gene**	**aCPB**	**pCPB**	**24H**
**S100**β **vs. BL**					
2nd mess cell signaling	rs198388	*NPPB*		x	
2nd mess cell signaling	rs4477886	*ROCK2*			x
Acid base cell vol regulation	rs4973768	*SLC4A7*			x
Apoptotic factors	rs3749166	*CAPN10*		x	x
Estrogen	rs4986938	*ESR2*	x		
Inflammation	rs2228145	*IL6R*			x
Inflammation	rs2227631	*SERPINE1*		x	
Inflammation	rs1640827	*TLR5*	x		
Inflammation	rs1800629	*TNF*	x	x	
Neurotransmitters	rs1801253	*ADRB1*	x		
Neurotransmitters	rs211014	*GABRG2*		x	
Platelets and coagulation	rs2070011	*FGA*			x
Protein chaperone repair	rs1617640	*EPO*	x		x
Protein chaperone repair	rs2763979	*HSPA1B*			x
**NFH vs. BL**					
2nd mess cell signaling	rs10061804	*NPR3*		x	
Acid base cell vol regulation	rs9951307	*AQP4*		x	
Apoptotic factors	rs1805419	*BAX*			x
Apoptotic factors	rs3900115	*CASP10*	x		
Apoptotic factors	rs3769827	*CASP8*	x		
Apoptotic factors	rs937283	*MDM2*	x	x	
Calcium regulation	rs815815	*CALM2*		x	
Inflammation	rs1126757	*IL11*	x		
Inflammation	rs2298885	*IL11*	x		x
Inflammation	rs230529	*NFKB1*		x	
Inflammation	rs5361	*SELE*	x		x
Inflammation	rs237025	*SUMO4*		x	x
Inflammation	rs1866389	*THBS4*	x		
Neurotransmitters	rs553668	*ADRA2A*			x
Neurotransmitters	rs1461225	*GRIA1*		x	
Other	rs10423702	*NOTCH3*	x		
Peroxidation	rs6917589	*SOD2*		x	x
Platelets/Coagulation	rs1654431	*GP6*		x	
Vascular regulation	rs1042713	*ADRB2*			x

*x- P < 0.05 for association without correction for multiple comparisons. Blood samples were drawn immediately after induction of anesthesia (baseline-BL), just prior to CPB (aCPB), 1 h after the end of CPB (pCPB), and 24 h (24 H) postoperatively*.

In order to make a preliminary assessment of relative weights of specific genes in the contribution to biomarker elevations at different times, multiple regression equations were calculated for changes in NFH and S100β at pCPB and 24H times related to SNPs. The results of the multiple regression analyses depicting the most highly weighted SNPs are shown in Table 4, indicating disparate genes (and presumably downstream proteins) with varying weights associating with changes in biomarkers and with a different pattern of associations at the two times post-CPB.

**Table 4:** Regression data: change in biomarkers from BL: pCPB and 24H.

**Variable**	**Coefficient**	**Std. Error**	***t-*value**	***p*-value**	**Gene**
**NFH (EQUATION 1) CHANGE AT pCPB**					
(Intercept)	−0.24	0.10	−2.36	2.25e-02	NA
rs6917589	−0.20	0.06	−3.29	1.95e-03	*SOD2*
rs237025	0.11	0.05	2.22	3.14e-02	*SUMO4*
rs1654431	0.10	0.05	2.10	4.12e-02	*GP6*
rs815815	−0.09	0.06	−1.45	1.53e-01	*CALM2*
rs937283	−0.09	0.06	−1.47	1.50e-01	*MDM2*
rs230529	0.08	0.05	1.64	1.07e-01	*NFKB1*
**S100**β **(EQUATION 2) CHANGE AT pCPB**					
(Intercept)	2.38	0.35	6.80	6.34e-09	NA
rs1800629	0.57	0.27	2.09	4.15e-02	*TNF*
rs3749166	0.55	0.21	2.57	1.28e-02	*CAPN10*
rs198388	−0.50	0.21	−2.40	1.97e-02	*NPPB*
rs2227631	0.43	0.19	2.23	2.99e-02	*SERPINE1*
**NFH (EQUATION 3) CHANGE AT 24H**					
(Intercept)	−1.34	0.30	−4.42	8.09e-05	NA
rs553668	0.28	0.12	2.39	2.20e-02	*ADRA2A*
rs5361	−0.21	0.10	−2.05	4.77e-02	*SELE*
rs1805419	0.21	0.10	2.17	3.61e-02	*BAX*
rs237025	0.17	0.08	1.99	5.37e-02	*SUMO4*
rs2298885	0.13	0.10	1.37	1.80e-01	*IL11*
rs1042713	0.13	0.09	1.48	1.47e-01	*ADRB2*
rs6917589	0.13	0.09	1.38	1.77e-01	*SOD2*
First ICU Glc	4.42*e*−03	1.71e-03	2.59	1.35e-02	NA
**S100**β **(EQUATION 4) CHANGE AT 24H**					
(Intercept)	−1.50	0.87	−1.73	9.06e-02	NA
rs4973768	0.57	0.22	2.53	1.46e-02	*SLC4A7*
rs2763979	0.55	0.21	2.60	1.21e-02	*HSPA1B*
rs2070011	−0.50	0.23	−2.17	3.45e-02	*FGA*
rs3749166	0.40	0.21	1.94	5.85e-02	*CAPN10*
rs4477886	0.40	0.22	1.83	7.29e-02	*ROCK2*
rs1617640	−0.29	0.22	−1.32	1.92e-01	*EPO*
Age	0.02	0.01	2.31	2.50e-02	*NA*

## Discussion

### Interpretation of data

Our biomarker data in humans after cardiopulmonary bypass, a procedure associated with subtle ischemic brain damage (50), support, but do not prove, the notion that multiple specific pathophysiologic processes, with variable contributing importance, are likely involved in the pathogenesis of ischemic brain damage in humans. Nonetheless, our data suggest that the importance of different pathways differ according to biomarker and may change with time post insult. From these data we hypothesize that neuroprotection studies, in order to be successful, must be multifaceted, focused on demonstrably important pathways, and, moreover, the nature of the multifaceted therapy may change with time after the onset of the ischemic insult.

The human model we chose was based on the relative homogeneity and reproducibility of the ischemic insult with previously reported increases in neural biomarkers which have been associated with neurologic outcomes. Cardiopulmonary bypass such as we studied, is associated with cerebral ischemia which can have relatively silent impact but produces abnormalities in postoperative MRI (50), biomarkers of brain injury which vary with specific SNPs (56), and neuropsychiatric changes (57).

S100β and hypophosphorylated neurofilament H (NFH) were assessed as biomarkers of brain injury for this study. Both biomarkers were chosen based on neural element origins and prior reports indicating elevations after ischemic insults and associations of such elevations with neurologic outcome. The S100β protein is thought to derive from astrocytes (52, 58). Increases in S100β have been associated with neurologic outcomes after cardiac surgery (47, 59) cardiac arrest (60) and stroke (61). The major weakness with S100β in our study is observations of very high levels reported in cardiotomy and mediastinal blood with suggestions that injured pericardial or myocardial tissues may be contributing to early post-CPB elevations (62), likely an important element in the S100β increases we observed at pCPB. Notwithstanding these reasonable concerns there are ample reports in other contexts of S100β clearly being related to volume of brain injury on imaging and with clinical outcomes (47, 59, 60, 63), such that the increase in mean S100β we observed at pCPB likely represents both neural and non-neural release of the protein. Notably NFH, based on structural neural proteins released with neuronal degeneration (48), would not be expected to be associated with cardiotomy suction admixture, although this has not been formally evaluated. The lack of a comparable abrupt rise in mean NFH concentration at pCPB in our data supports the notion that NFH is not contaminated by cardiotomy suctioned blood.

NFH was developed as a biomarker based on a review of abundant structural neural proteins by coauthor Siman with preclinical studies indicating NFH release from degenerating cultured neurons (48). NFH elevations in cerebrospinal fluid have been observed in previous studies involving cardiac surgery (49). Moreover other studies in humans report increases in NFH in CSF of patients sustaining traumatic brain injury (TBI) (64), DHCA (49), and subarachnoid hemorrhage (SAH) with vasospasm (65), and with outcome in SAH linked to CSF NFH levels (65). Some increase in NFH has been observed in serum of patients after TBI relative to controls but link to neurological outcome was not evaluated (64).

In an effort to avoid an extremely large sample size that would be required with a more standard genome wide association study, biologically active SNPs representing protein function and pathways already reported to be involved in the pathogenesis of ischemic brain damage were studied. This of course provides an inherent bias of the investigator choosing the SNPs (WAK) but nonetheless allows us to better ascertain the primary goal of demonstrating involvement of multiple pathways and proteins contributing to post-ischemic brain damage. Clearly, previously unknown biological contributors could be missed with this approach and is a consideration for future studies. Nonetheless, this study is underpowered to confidently offer any valid conclusions about specific SNPs studied but does suggest important hypotheses regarding the notions of multiple involved genes and function of their downstream proteins, varying relative weights, and temporal changes in disparate proteins involved in pathogenesis of ischemic brain damage. Moreover, the nominal SNP associations before multiple comparison adjustment, although possibly a random finding, may suggest a smaller more focused SNP sample for future studies.

### Theoretical basis to reframe neuroprotection research

Our protocol was designed to explore the feasibility of a novel approach, using functional genomics to evaluate the role of genetic variation in specific multifactorial vulnerability to ischemia. This sort of approach should contribute to the theoretical underpinnings for rational design of multifaceted clinical neuroprotection trials. Notably, the main obstacle in devising a multifaceted approach to neuroprotection is determining the physiological and biochemical processes to target…. logically expected to be those which are most heavily weighted in terms of impact on ischemic outcome and the correct timing of such therapy. Our regression analyses were done in an endeavor to address this issue, suggesting that multiple disparate genes (and likely disparate function of downstream proteins) involved in pathogenesis of ischemic brain damage have varying contributing weights which vary temporally post insult. This forms the basis for the theoretical rationale subsequently described in more detail.

In a biological system undergoing a complex injury characterized by severity, **S**, an equation can be derived comprised of numerous pathophysiologic factors, **F**_*i*_, and weighting factors, **W**_*i*_ that vary with post-insult time (66, 67)***, t*:**

(1) $$S_{\text{t}}\;=\;W_{\text{1t}}F_{1}+W_{\text{2t}}F_{2}+W_{\text{3t}}F_{3}+W_{\text{4t}}F_{4}+W_{\text{5t}}F_{5}\ldots$$

This overall concept, originally presented by Kofke (16) was the basis for performing the multiple regression analysis of our data, where the weighting factors are our regression coefficients.

DNA based systems have inherent variation in genes and in translation of important proteins thereby leading to a large number of disparate and interacting factors (14, 68–72) contributing to **S** with also an unknown number of as yet unknown other factors with correspondingly unknown variability and weighting. Such uncertainty supports the need to do proper foundational studies before clinical efficacy trials.

This functional genomics approach lays the groundwork for larger studies; perhaps targeting conventional cerebral ischemia syndromes such as stroke, vasospasm, and cardiac arrest; to determine more conclusively the most important pathways and proteins contributing to susceptibility to neural damage after such ischemic insults. This can be expected to lead to a systems biology (67, 73) type detailed genome-linked description of the pathophysiology of human cerebral ischemia. This approach will likely include vertical gene-linked phenotypes assessments (74) of the pathophysiology of cerebral ischemia, including the potentially pivotal role of so called “hub” pathways (39). Such validation in larger studies will involve mechanistically different biomarkers demonstrated to be predictive of outcome, e.g., microdialysis data, magnetic resonance imaging and spectroscopy, various other blood/CSF biomarkers, and so on. Moreover, if and when methods become available that indicate changes in the cerebral transcriptome (75, 76) post-insult then potentially even more relevant information may become available. Neuroprotection based on these concepts will necessarily be multifaceted with the specific combinations of therapies based on weighting factors which also vary with time post insult, as suggested by the pathophysiologic multiple regression equations parameters in Table 4 and further illustrated in Table 5.

**Table 5:** Example of possible multifaceted therapy derived from post CPB-based functional genomic analysis.

**SNP**	**Symbol (regression coefficient)**	**Gene name**	**Gene function**	**Plausible therapeutic strategy**
**pCPB**				
rs6917589	*SOD2* (−0.20)	Superoxide dismutase 2	This gene is a member of the iron/manganese superoxide dismutase family. It encodes a mitochondrial protein that forms a homotetramer and binds one manganese ion per subunit. This protein binds to the superoxide byproducts of oxidative phosphorylation and converts them to hydrogen peroxide and diatomic oxygen [provided by RefSeq, Apr 2016]	•Antioxidant drugs
rs3749166	*CAPN10* (+0.55)	Calpain 10	Calpains represent a ubiquitous, well-conserved family of calcium-dependent cysteine proteases. The calpain proteins are heterodimers consisting of an invariant small subunit and variable large subunits. The large catalytic subunit has four domains: domain I, the N-terminal regulatory domain that is processed upon calpain activation; domain II, the protease domain; domain III, a linker domain of unknown function; and domain IV, the calmodulin-like calcium-binding domain. This gene encodes a large subunit. It is an atypical calpain in that it lacks the calmodulin-like calcium-binding domain and instead has a divergent C-terminal domain. It is similar in organization to calpains 5 and 6. [provided by RefSeq, Sep 2010]	•Calpain inhibitor drugs •Tight glucose control
rs198388	*NPPB* (−0.50)	Natriuretic peptide B	This gene is a member of the natriuretic peptide family and encodes a secreted protein which functions as a cardiac hormone. The protein undergoes two cleavage events, one within the cell and a second after secretion into the blood. The protein's biological actions include natriuresis, diuresis, vasorelaxation, inhibition of renin and aldosterone secretion, and a key role in cardiovascular homeostasis. A high concentration of this protein in the bloodstream is indicative of heart failure. The protein also acts as an antimicrobial peptide with antibacterial and antifungal activity. [provided by RefSeq, Nov 2014]	•Natriuretic hormones •Angiotensin converting enzyme inhibitors •Angiotensin receptor blockers
rs2227631	*SERPINE1* (+0.43)	Serpin family E member 1	This gene encodes a member of the serine proteinase inhibitor (serpin) superfamily. This member is the principal inhibitor of tissue plasminogen activator (tPA) and urokinase (uPA), and hence is an inhibitor of fibrinolysis. [provided by RefSeq, Sep 2009]	•Anticoagulation
rs237025	*SUMO4* (+0.17)	Small ubiquitin-like modifier 4	This gene is a member of the SUMO gene family. This family of genes encode small ubiquitin-related modifiers that are attached to proteins and control the target proteins' subcellular localization, stability, or activity. The protein described in this record is located in the cytoplasm and specifically modifies IKBA, leading to negative regulation of NF-kappa-B-dependent transcription of the IL12B gene. The RefSeq contains this polymorphism. [provided by RefSeq, Jul 2008]	•Anti-inflammatory?
**24H**				
rs553668	*ADRA2A* (+0.28)	Adrenoceptor alpha 2A	Alpha-2-adrenergic receptors are members of the G protein-coupled receptor superfamily. They include 3 highly homologous subtypes: alpha2A, alpha2B, and alpha2C. These receptors have a critical role in regulating neurotransmitter release from sympathetic nerves and from adrenergic neurons in the central nervous system. [provided by RefSeq, Jul 2008]	•Alpha-2 receptor drugs •Yohimbine •dexmedetomidine
rs5361	*SELE* (−0.21)	Selectin E	The protein encoded by this gene is found in cytokine-stimulated endothelial cells and is thought to be responsible for the accumulation of blood leukocytes at sites of inflammation by mediating the adhesion of cells to the vascular lining. These proteins are part of the selectin family of cell adhesion molecules. Adhesion molecules participate in the interaction between leukocytes and the endothelium and appear to be involved in the pathogenesis of atherosclerosis. [provided by RefSeq, Jul 2008]	•Anti-inflammatory drugs with interfere with leukocyte adhesion •glucocorticoids
rs1805419	*BAX* (+0.21)	BCL2 associated X, apoptosis regulator	The protein encoded by this gene belongs to the BCL2 protein family. BCL2 family members form hetero- or homodimers and act as anti- or pro-apoptotic regulators that are involved in a wide variety of cellular activities. This protein forms a heterodimer with BCL2, and functions as an apoptotic activator. This protein is reported to interact with, and increase the opening of, the mitochondrial voltage-dependent anion channel (VDAC), which leads to the loss in membrane potential and the release of cytochrome c. The expression of this gene is regulated by the tumor suppressor P53 and has been shown to be involved in P53-mediated apoptosis. [provided by RefSeq, Jul 2008]	•Anti-apoptotic drugs •Drugs with affect mitochondrial permeability pore, e.g., cyclosporine
rs4973768	*SLC4A7* (+0.57)	Solute carrier family 4 member 7	This locus encodes a sodium bicarbonate cotransporter. The encoded transmembrane protein appears to transport sodium and bicarbonate ions in a 1:1 ratio, and is thus considered an electroneutral cotransporter. The encoded protein likely plays a critical role in regulation of intracellular pH involved in visual and auditory sensory transmission. [provided by RefSeq, Apr 2012]	•Drugs increasing intracellular pH •THAM, tromethamine •Citrate •Hyperventilation
rs2763979	*HSPA1B* (+0.55)	Heat shock protein family A (Hsp70) member 1B	This intronless gene encodes a 70 kDa heat shock protein which is a member of the heat shock protein 70 family. In conjunction with other heat shock proteins, this protein stabilizes existing proteins against aggregation and mediates the folding of newly translated proteins in the cytosol and in organelles. [provided by RefSeq, Jul 2008]	•Heat shock protein supporting therapy •Ischemic preinduction
rs2070011	*FGA* (−0.50)	fibrinogen alpha chain	This gene encodes the alpha subunit of the coagulation factor fibrinogen, which is a component of the blood clot. Following vascular injury, the encoded preproprotein is proteolytically processed by thrombin during the conversion of fibrinogen to fibrin. Mutations in this gene lead to several disorders, including dysfibrinogenemia, hypofibrinogenemia, afibrinogenemia and renal amyloidosis [provided by RefSeq, Jan 2016]	•Anticoagulation

*Data from Table 4 with regression coefficients with P < 0.05 were included in this table indicating how such information might be used to develop a multifaceted neuroprotection strategy in cardiac surgery. The drugs and therapies suggested are based on an association with noted genes but without regard to the whether the coefficient is positive or negative. A high and statistically significant coefficient was used to indicate importance of a pathway. Therapies suggested were then made based on prior knowledge or data indicating a neuroprotective potential. Notably this table supports the importance of a potential therapy with regard to time after CPB*.

A further complicating factor includes the notion that there are endogenous biological accommodation factors which may attenuate or worsen an insult. Such factors will need to be considered in future studies exploring pathophysiologic pathways, understanding that the impact of a pathway may be exacerbated or attenuated over time post insult based on such endogenous responses. For example cerebral ischemia/acidosis begets spontaneous hyperventilation (77–80) and systemic hypertension is a typical concomitant of brainstem ischemia (81). Other examples include ischemia-mediated adjustments in protein transcription and ischemic preconditioning (82–90) and issues in resilience or plasticity of pathophysiological networks, e.g., apoptosis networks (91, 92). In addition, other peri-insult time related epigenetic processes that may affect the transcription and translation of a gene (93, 94) may also be included in these issues.

Although not specifically addressed in our analyses many authors have suggested that biological complexity is further embellished by social factors e.g., nursing numbers relative to patient numbers (and complexity), health care worker experience, availability of pharmaceuticals and innovative equipment, quality of emergency teams, local cultures and ethics related to safety, cost containment, and morale, practice variation, and so on (19–21, 95, 96), which are also important to the outcome severity of injury. Notably, inter-center variance has been reported as a very important element in the quality, complexity, and reproducibility of clinical research (19, 20, 36, 97). This is one of the factors underlying the Multi-PART initiative (32) which one may surmise will simulate such inter-center variation in creating a realistic real-word environment for preclinical trials (33, 98).

**Figure 3:**
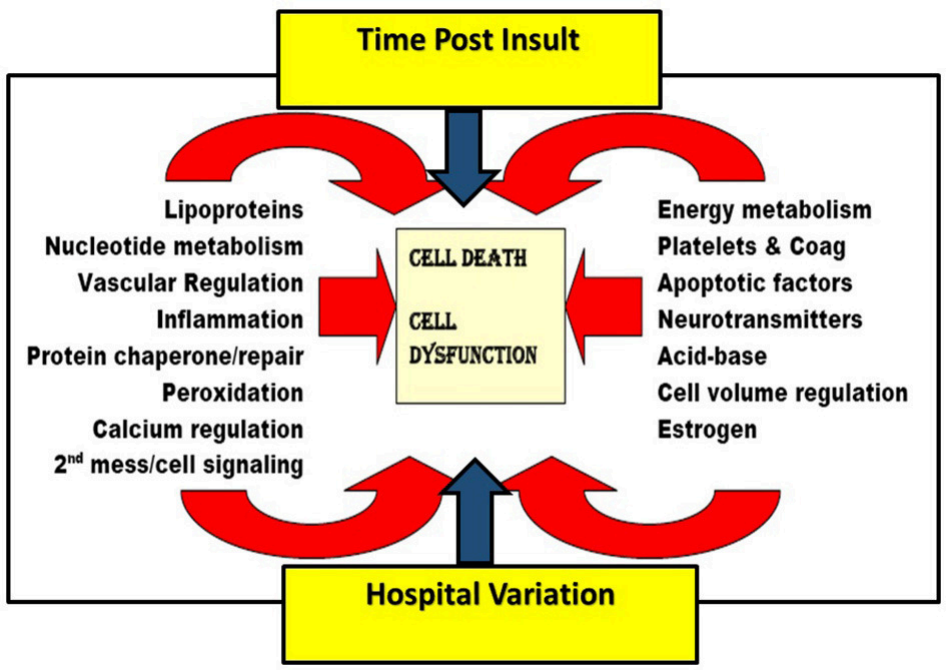
Schematic depicting many of the pathways involved in the pathogenesis of ischemic brain injury, illustrating the impact of time post ischemia and health system variation on multiple involved pathways. Figure adapted and altered from Kofke (16).

Such multiple interactions and complexity are summarized in Figure 3 and have been suggested in pathophysiology reviews by Lipton (68) and others (14, 20, 40, 70–72, 91, 92, 99). Given the numerous highly variable, possibly nonlinear, biological and health system/social factors that contribute to post insult brain damage, it is unsurprising that clinical studies directed at improving only one of numerous complex interacting factors tend to show no or little effect. This is particularly true in multi-institutional trials (increasing social variation), unless it is truly a breakthrough phenomenon [large W for brain blood flow as seen in early endovascular thrombectomy in ischemic stroke (100)] or if the therapy itself exerts a multifaceted effect [e.g., hypothermia (101)]. This then leads to the notion that the current widely practiced methods of advancing clinical therapy for complex problems is generally a fruitless waste of public resources and that an alternate method is needed which is based on a multifactorial approach. Rogalewski et al. (8), Candelario et al. (15), Ginsberg (11), and O'Collins et al. (13) have reviewed and endorsed this concept; however, they and others who endorse the notion of combination therapy (5, 6, 10) do not suggest a rational method for establishing foundational information regarding time-dependent weights of various facets, and then building a multimodal approach rather than trying everything at once…another prescription for failure. A rational method for introducing neuroprotective therapies in research protocols is needed…indeed, the entire field of neuroprotection research needs to be reframed in a manner which accounts for these important pathophysiological principles.

### An example of implementation

We propose fairly abstract concepts relating to biological complexity which may be used to guide development of neuroprotection strategies. We therefore, suggest that foundational human research is needed to first define the importance of potential target pathways and proteins at various times after insult. And only after such information is garnered should any further neuroprotection trials be attempted. Such trials optimally will be multifaceted based on the recommended foundational research. An example of how this might happen using our data follows.

The most important genes from the regression tables with *P* < 0.05 are delineated in Table 5 with a description of their function taken from the www.ncbi.nlm.nih.gov web pages describing various aspects of individual genes. From these descriptions one can make inferences, and generate concrete ideas regarding the most important proteins and pathways involved in the development of post-CPB brain damage as indicated by the biomarker surrogates used in these studies. Overall, if further validated, this suggests that a multifaceted approach to neuroprotection in the context of cardiac surgery would be different early post CPB vs. the next day and that specific elements of a multifaceted approach could be as suggested in Table 5. Based on this table one might imagine a neuroprotective cocktail, grounded also in preclinical studies, being developed comprised at CPB of tirilazad (102) (antioxidant), to-be-developed anti-apoptotic drug, tight glucose control, nasiritide (103), enalopril (104), and tranexamic acid (105) and 24 h later provide a neuroprotective cocktail comprised of dexmedetomidine (106), cyclosporine (107), glucocorticoids, tromethamine (THAM) (108, 109), and to-be-developed heat shock protein supporting drugs or gene therapy (110). Note that this is an entirely theoretical construct based on our data which we know is not compelling and is in need of future studies corroborating this approach to building a foundation for logically designed neuroprotection. We are simply describing a concrete application example of the notions presented.

This hypothesized strategy to developing neuroprotective therapies is necessarily population-based, i.e., definition of important factors and their time-dependent weighting factors are derived from and applied to populations of patients. The possibility also exists, if genomics analyses could be done quickly, or a patient's genome is available in the medical record (111), to design individualized multifaceted neuroprotection strategies on hospital admission or preoperatively. Given current work to include patients' genomes in their medical records (111, 112), and link such data to phenotypes (74) this may be the time-based multifaceted personalized neuroprotection strategy of the future.

## Conclusion

We describe putative associations of biomarkers of ischemic brain damage with variation in polymorphisms of multiple genes, suggesting expected variation in downstream protein function, relevant to the pathogenesis of ischemic brain damage. Phenotype-linked genetic and epigenetic variation may be useful to indicate the most important time-dependent biochemical proteins and pathways involved in the pathogenesis of ischemic damage. This approach, if amplified with larger population-based or personalized innovations, may provide a theoretical basis for the transformation and reframing in the approach to neuroprotection as advocated by Donnan a decade ago.

## Author contributions

WK: study design, oversight, data interpretation, primary author of manuscript, sole author of most of the concepts in introduction and discussion; YR: statistical analysis; JA and JB: study design and implementation; HL: study design, statistical genomics strategy, data interpretation; KN: genomics oversight and interpretation; RS: NFH analysis; QM: S100 analysis; WB: S100 analysis; SY and GK: data analysis; CK: genomic analysis. All authors contributed to manuscript revision, read and approved the submitted version.

### Conflict of interest statement

The authors declare that the research was conducted in the absence of any commercial or financial relationships that could be construed as a potential conflict of interest.
