# Supplementary material for: Reframing the Biological Basis of Neuroprotection Using Functional Genomics: Differentially Weighted, Time-Dependent Multifactor Pathogenesis of Human Ischemic Brain Damage
Source: Front Neurol. 2018 Jun 26;9:497. doi: 10.3389/fneur.2018.00497 (PMC6028620; doi:10.3389/fneur.2018.00497)
Supplement: Supplementary file 1 [file Presentation_1.PDF]

SUPPLEMENTARY MATERIAL

Reframing Neuroprotection: Differentially Weighted, Time-Dependent Multifactor Pathogenesis of Human Ischemic Brain Damage

W Andrew Kofke*, Yue Ren, MA, John G Augoustides, Hongzhe Li, Katherine Nathanson, Robert Siman, Qing Cheng Meng, Weiming Bu, Sukanya Yandrawatthana, Guy Kositratna, Cecilia Kim, Joseph E. Bavaria

*Correspondence:
W Andrew Kofke
kofkea@uphs.upenn.edu

**Table I. SNPs Evaluated; According to Ischemic Axes *With Literature Citations***			
**Ischemic Axis**	**SNP ID**	**SNP Symbol**	**Gene Name**
**Lipoproteins[1-6]**	rs7412	*APOE*	apolipoprotein E
	rs1050283	*OLR1*	oxidized low density lipoprotein (lectin-like) receptor 1
	rs268	*LPL*	lipoprotein lipase
	rs35068180	*MMP3*	matrix metallopeptidase 3
**Nucleotide Metabolism[7-9,3,10-13,1,2,14,15]**	rs17037390	*MTHFR*	methylenetetrahydrofolate reductase (NAD(P)H)
**Vascular Regulation[14,15,1,16-29]**	rs2070744	*NOS3*	nitric oxide synthase 3 (endothelial cell)
	rs2368564	*REN*	renin
	rs699	*AGT*	angiotensinogen (serpin peptidase inhibitor, clade A, member 8)
	rs5186	*AGTR1*	angiotensin II receptor, type 1
	rs1799998	*CYP11B2*	cytochrome P450, family 11, subfamily B, polypeptide 2 (Aldosterone Synthase
	rs2507800	*ANGPT1*	angiopoietin 1
	rs17086609	*FLT1*	fms-related tyrosine kinase 1
	rs5370	*EDN1*	endothelin 1
	rs1048101	*ADRA1A*	adrenoceptor alpha 1A
	rs1042713	*ADRB2*	adrenoceptor beta 2, surface
	rs10770141	*TH*	tyrosine hydroxylase
**Inflammation[30-32]**	rs2228145	*IL6R*	interleukin 6 receptor
	rs2069762	*IL2*	interleukin 2
	rs2298885	*IL11*	interleukin 11
	rs1126757	*IL11*	interleukin 11
	rs6136	*SELP*	selectin P (granule membrane protein 140kDa, antigen CD62)
	rs5361	*SELE*	selectin E
	rs2227631	*SERPINE1*	serpin peptidase inhibitor, clade E (nexin, plasminogen activator inhibitor type 1), member 1
	rs1800629	*TNF*	tumor necrosis factor
	rs1800872	*IL10*	interleukin 10
	rs1800469	*TGFB1*	transforming growth factor, beta 1

	rs1800471	*TGFB1*	transforming growth factor, beta 1
	rs230529	*NFKB1*	nuclear factor of kappa light polypeptide gene enhancer in B-cells 1
	rs237025	*SUMO4*	small ubiquitin-like modifier 4
	rs1866389	*THBS4*	thrombospondin 4
	rs4696480	*TLR2*	toll-like receptor 2
	rs1927911	*TLR4*	toll-like receptor 4
	rs1640827	*TLR5*	toll-like receptor 5
**Protein Chaperone/ Repair[33-40]**	rs1617640	*EPO*	erythropoietin
	rs2763979	*HSPA1B*	heat shock 70kDa protein 1B
	rs2075799	*HSPA1L*	heat shock 70kDa protein 1-like
**Peroxidation[41-46,3,47,2]**	rs2071409	*MPO*	myeloperoxidase
	rs1800668	*GPX1*	glutathione peroxidase 1
	rs6917589	*SOD2*	superoxide dismutase 2, mitochondrial
	rs7943316	*CAT*	catalase
	rs11018628	*NOX4*	NADPH oxidase 4
	rs10830963	*MTNR1B*	melatonin receptor 1B
**Calcium Regulation [2,48-51,37]**	rs1006737	*CACNA1C*	calcium channel, voltage-dependent, L type, alpha 1C subunit
	rs449383	*SLC8A1*	solute carrier family 8 (sodium/calcium exchanger), member 1
	rs2236957	*CACNA2D2*	calcium channel, voltage-dependent, alpha 2/delta subunit 2
	rs2274223	*PLCE1*	phospholipase C, epsilon 1
	rs815815	*CALM2*	calmodulin 2 (phosphorylase kinase, delta)
	rs2229116	*RYR3*	ryanodine receptor 3
**2nd messenger/cell signaling[37,52-56,3]**	rs5443	*GNB3*	guanine nucleotide binding protein (G protein), beta polypeptide 3
	rs9282801	*NOS2*	nitric oxide synthase 2, inducible
	rs4477886	*ROCK2*	Rho-associated, coiled-coil containing protein kinase 2
	rs5065	*NPPA*	natriuretic peptide A
	rs198388	*NPPB*	natriuretic peptide B
	rs10061804	*NPR3*	natriuretic peptide receptor C/guanylate cyclase C (atrionatriuretic peptide receptor C)

	rs16260	*CDH1*	cadherin 1, type 1, E-cadherin (epithelial)
**Energy Metabolism [3,18]**	rs659366	*UCP2*	uncoupling protein 2 (mitochondrial, proton carrier)
	rs647126	*UCP3*	uncoupling protein 3 (mitochondrial, proton carrier)
	rs2307449	*POLG*	polymerase (DNA directed), gamma
	rs841853	*SLC2A1*	solute carrier family 2 (facilitated glucose transporter), member 1
	rs17208239	*IRS1*	insulin receptor substrate 1
	rs3802230	*CYP11B2*	cytochrome P450, family 11, subfamily B, polypeptide 2
**Platelets and Coagulation[8,57,58,9,59,60]**	rs2070852	*F2*	coagulation factor II (thrombin)
	rs1799963	*F2*	coagulation factor II (thrombin)
	rs1654431	*GP6*	glycoprotein VI (platelet)
	rs2243093	*GP1BA*	glycoprotein Ib (platelet), alpha polypeptide
	rs2070011	*FGA*	fibrinogen alpha chain
	rs1800788	*FGB*	fibrinogen beta chain
	rs964617	*MCF2L*	MCF.2 cell line derived transforming sequence-like
**Apoptotic Factors[61-79]**	rs1805419	*BAX*	BCL2-associated X protein
	rs3900115	*CASP10*	caspase 10, apoptosis-related cysteine peptidase
	rs3769827	*CASP8*	caspase 8, apoptosis-related cysteine peptidase
	rs937283	*MDM2*	MDM2 oncogene, E3 ubiquitin protein ligase
	rs1042522	*TP53*	tumor protein p53
	rs3749166	*CAPN10*	calpain 10
**Neurotransmitters[80,81]**	rs1461225	*GRIA1*	glutamate receptor, ionotropic, AMPA 1
	rs6465084	*GRM3*	glutamate receptor, metabotropic 3
	rs2227283	*GRIK2*	glutamate receptor, ionotropic, kainate 2
	rs937254	*GCOM1*	GRINL1A complex locus 1
	rs3794087	*SLC1A2*	solute carrier family 1 (glial high affinity glutamate transporter), member 2
	rs2075572	*OPRM1*	opioid receptor, mu 1
	rs595725	*OPRD1*	opioid receptor, delta 1
	rs211014	*GABRG2*	gamma-aminobutyric acid (GABA) A receptor, gamma 2
	rs363333	*SLC18A2*	solute carrier family 18 (vesicular monoamine transporter), member

	rs553668	*ADRA2A*	adrenoceptor alpha 2A
	rs7678463	*ADRA2C*	adrenoceptor alpha 2C
	rs1801253	*ADRB1*	adrenoceptor beta 1
**Acid-Base/Cell Volume[82,83]**	rs4973768	*SLC4A7*	solute carrier family 4, sodium bicarbonate cotransporter, member
	rs1053074	*KCNJ10*	potassium inwardly-rectifying channel, subfamily J, member 10
	rs9951307	*AQP4*	aquaporin 4
**Estrogen[84]**	rs2234693	*ESR1*	estrogen receptor 1
	rs4986938	*ESR2*	estrogen receptor 2 (ER beta)
**Other[85]**	rs10423702	*NOTCH3*	notch 3

Nomenclature derived from the dbSNP database of the National Center for Biotechnology Information; http://www.ncbi.nlm.nih.gov/projects/SNP/

-

Supplementary Material Figure I: SNP Associations:

Individual SNP associations with nominal level of significance or inclustion in regression equations at $P<0.05$ are depicted in the following figures. Associations with absolute levels of S100β(pg/ml) and NFH((pg/25µl) and with changes in both biomarkers (ch S100β and chNFH) from baseline are shown. Data depicted are the absolute biomarker concentrations or changes in concentrations for each denoted polymorphism depicted by rs identifier nomenclature, all of which are identified and referenced in supplemental Table I.

Abbreviations: NFH-Neurofilament H, BL-baseline, aCPB-1hour prior to cardiopulmonary bypass, pCPB-1 hour after cardiopulmonary bypass, 24h-24 hours after cardiopulmonary bypass

NFH Absolute Values Associations-BL (NFH1)

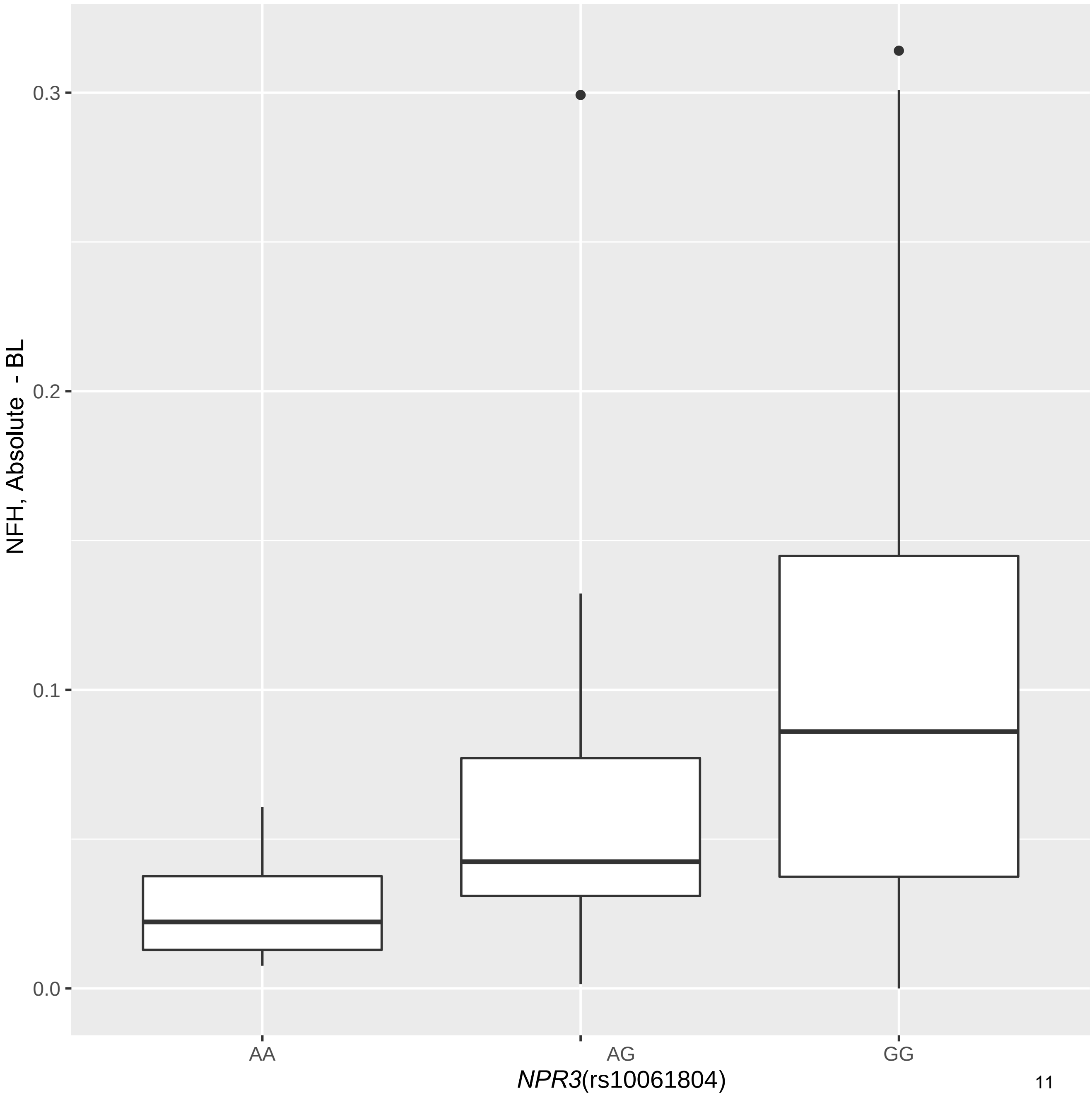
NFH, Absolute - BL
0.3
0.2
0.1
0.0
AA
AG
GG
NPR3(rs10061804)

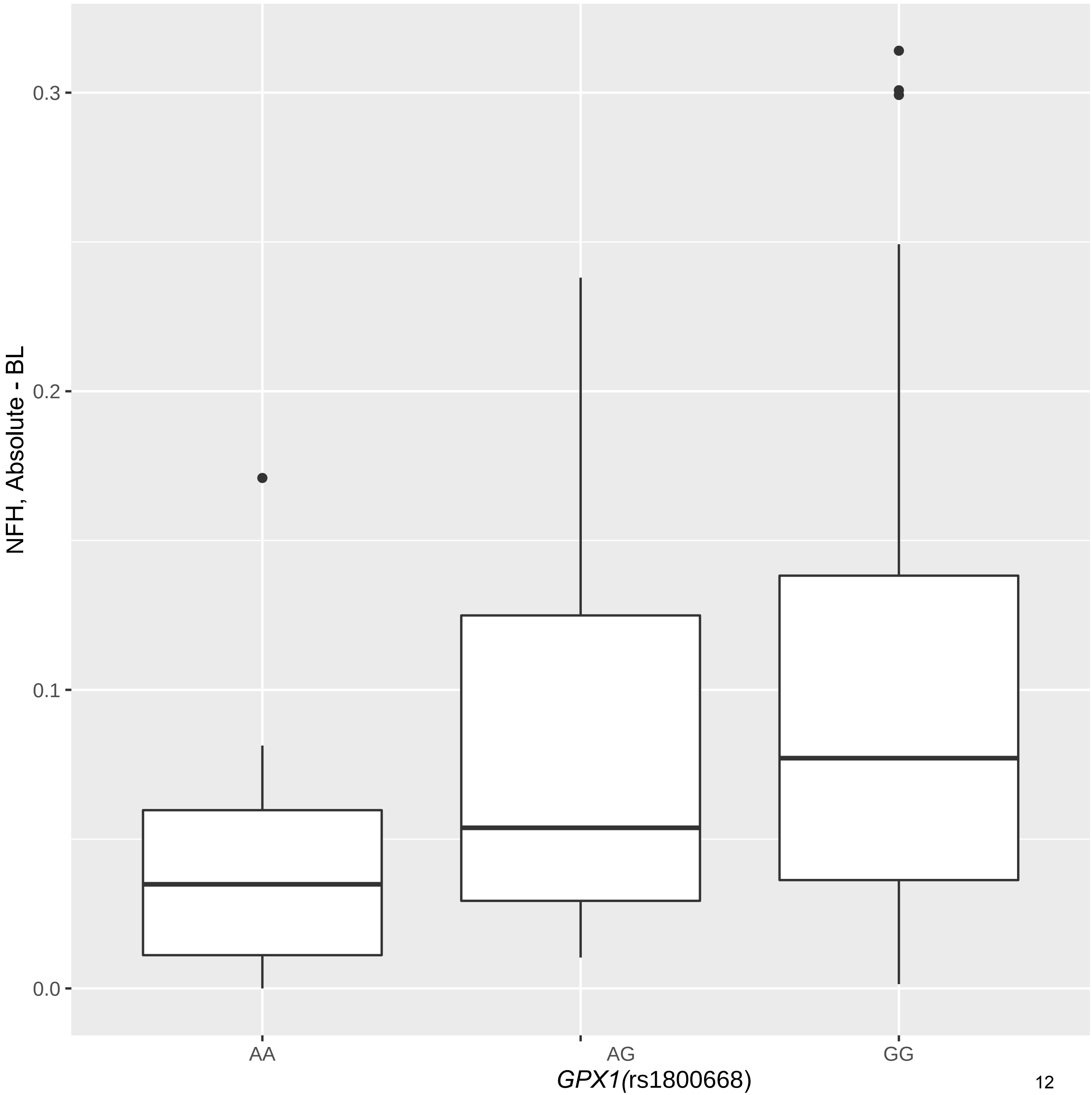
NFH, Absolute - BL
0.3
0.2
0.1
0.0
AA
AG
GG
GPX1(rs1800668)

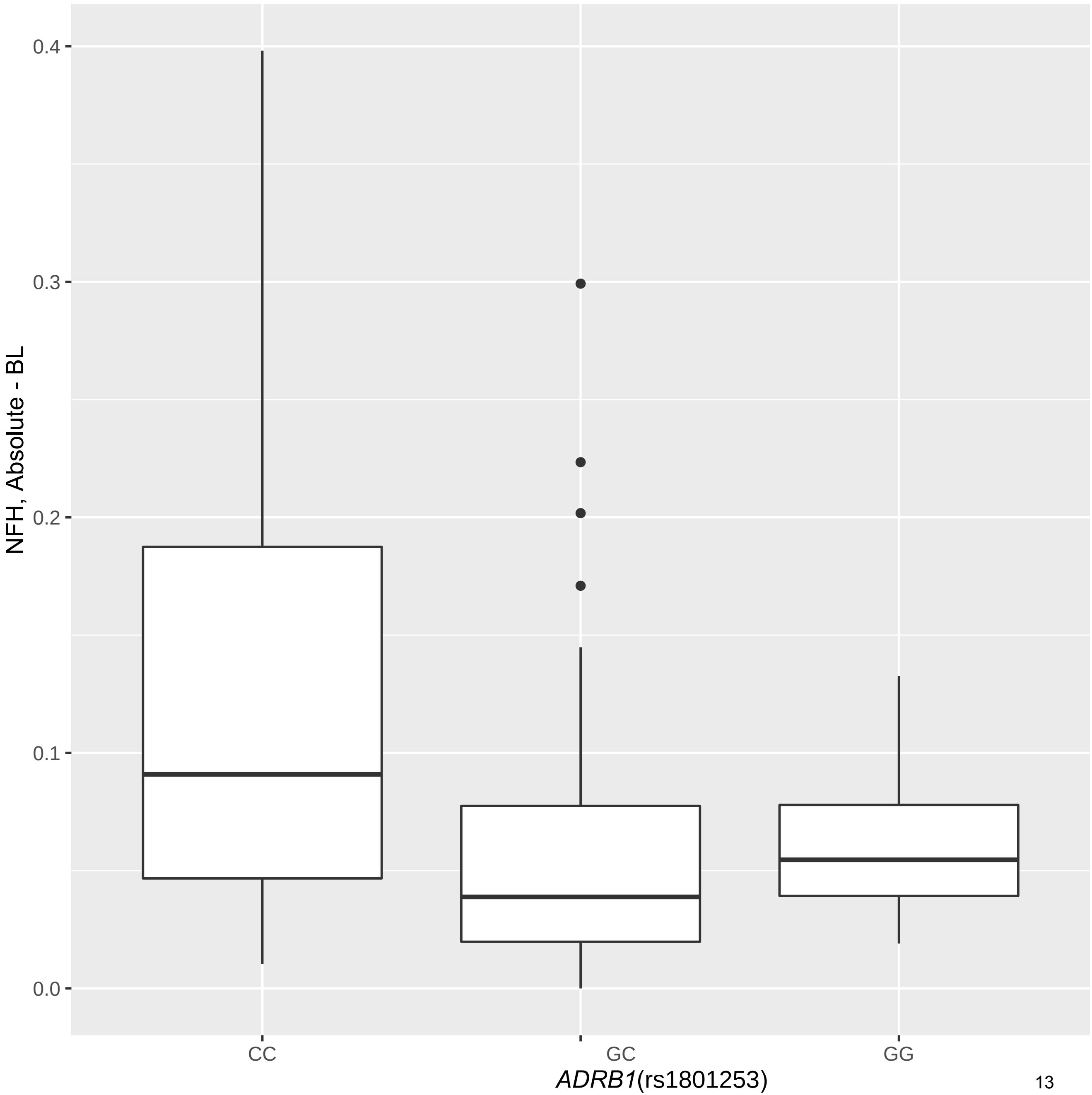
0.4
0.3
0.2
0.1
0.0
NFH, Absolute - BL
CC
GC
GG
ADRB1(rs1801253)

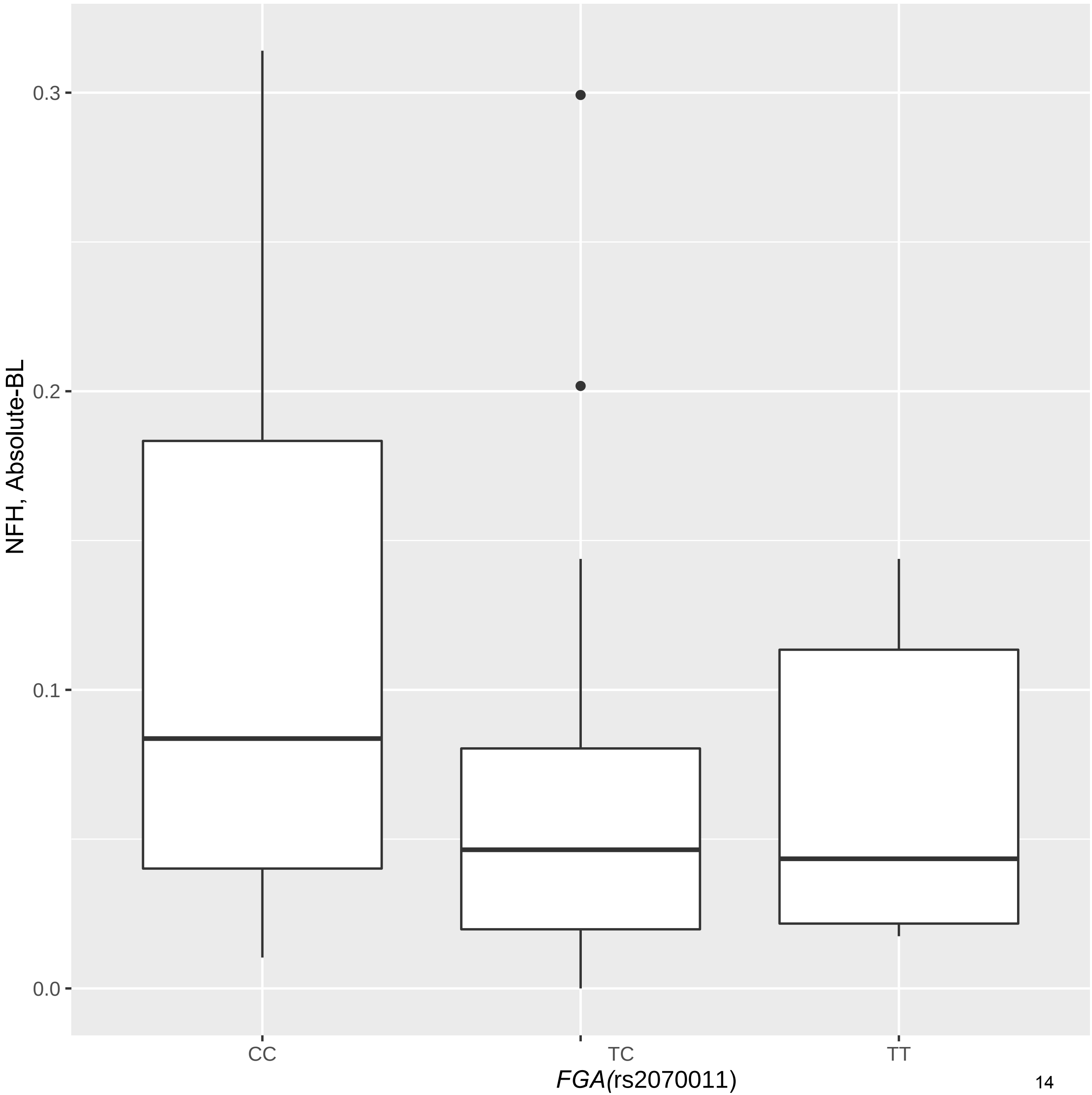
NFH, Absolute-BL
0.3
0.2
0.1
0.0
CC
TC
TT
FGA(rs2070011)

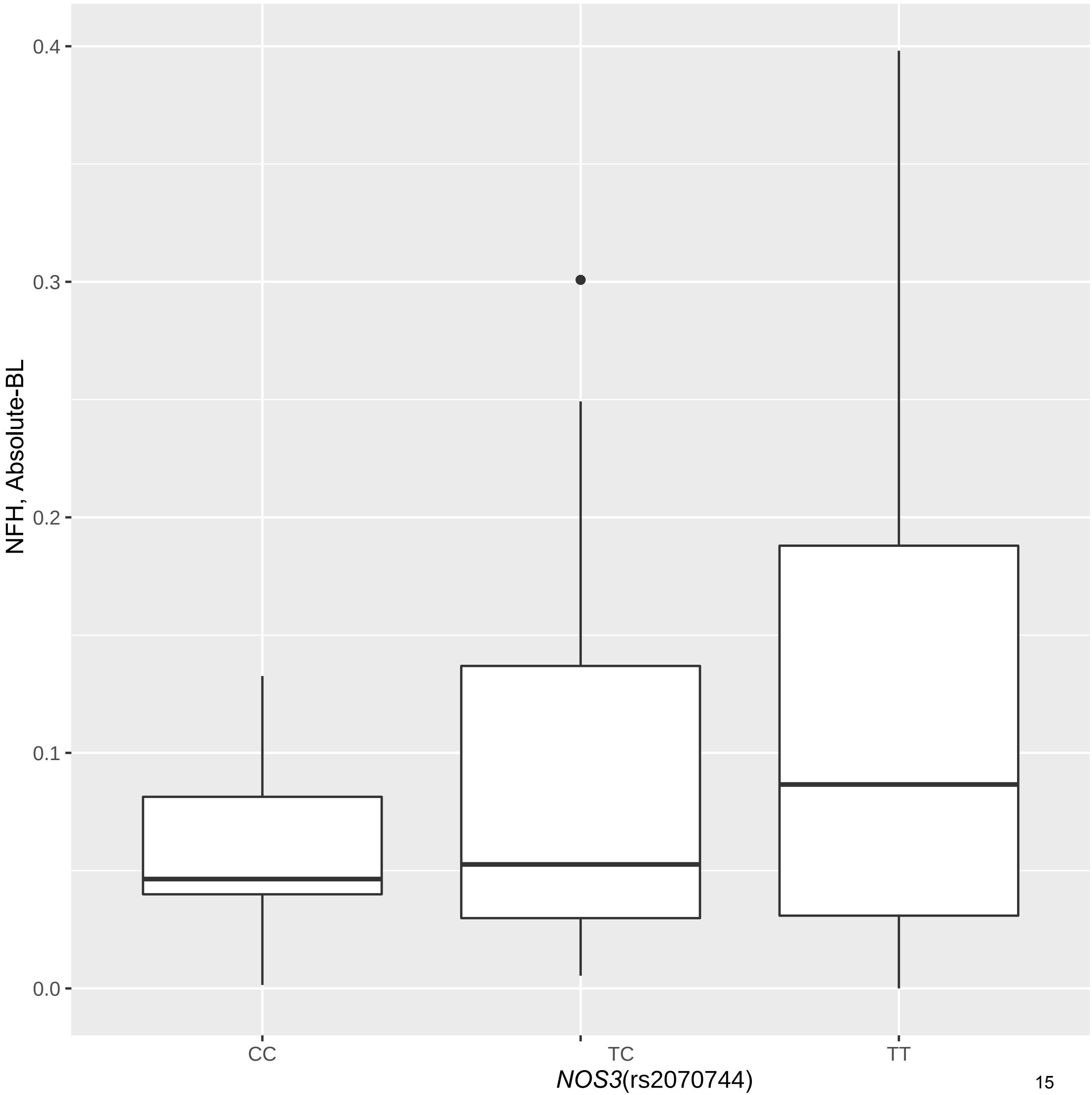
NFH, Absolute-BL
0.4
0.3
0.2
0.1
0.0
CC
TC
TT
NOS3(rs2070744)

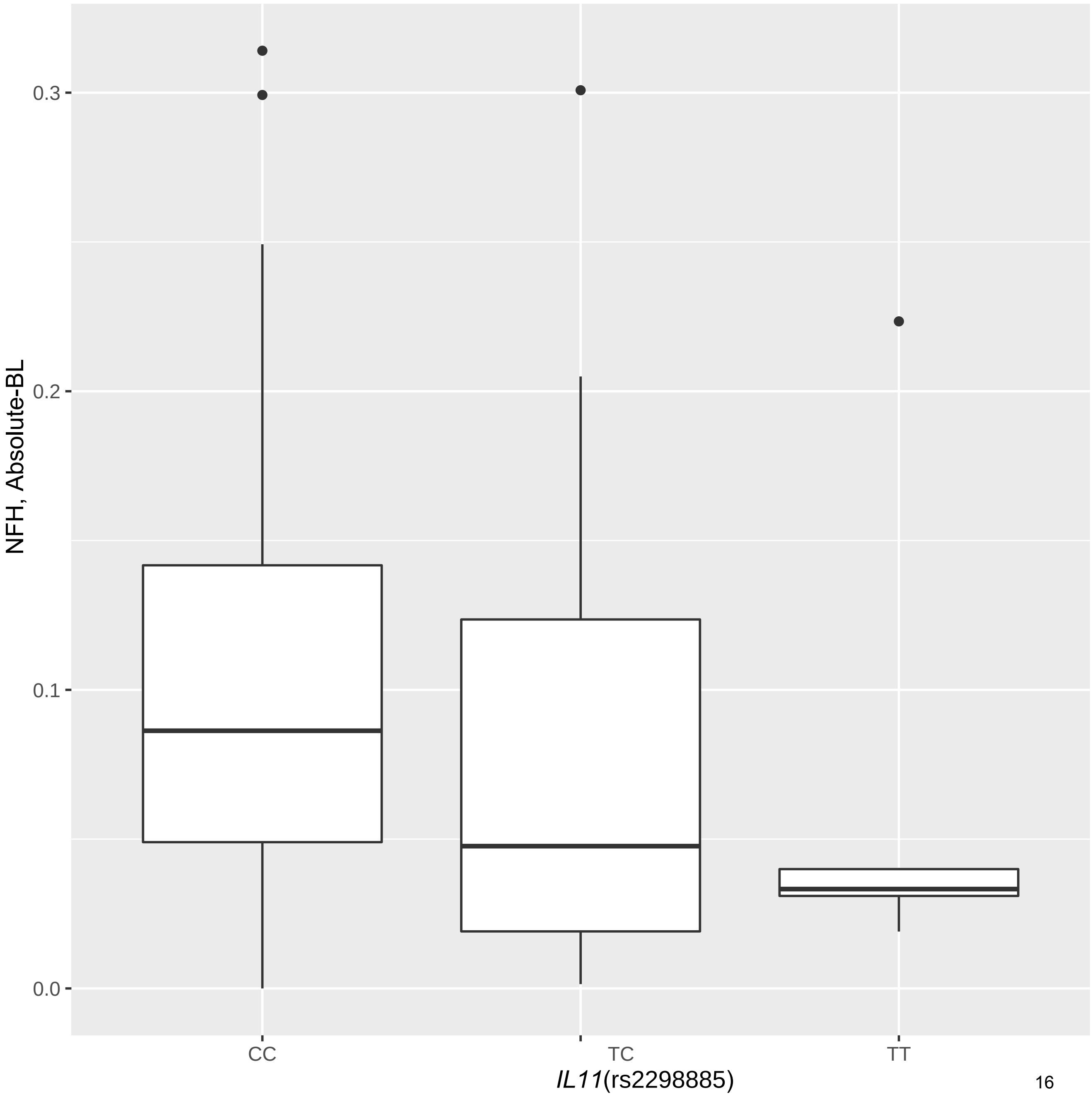
0.3
0.2
0.1
0.0
NFH, Absolute-BL
CC
TC
TT
IL11(rs2298885)

NFH Absolute Values Associations-aCPB (NFH2)

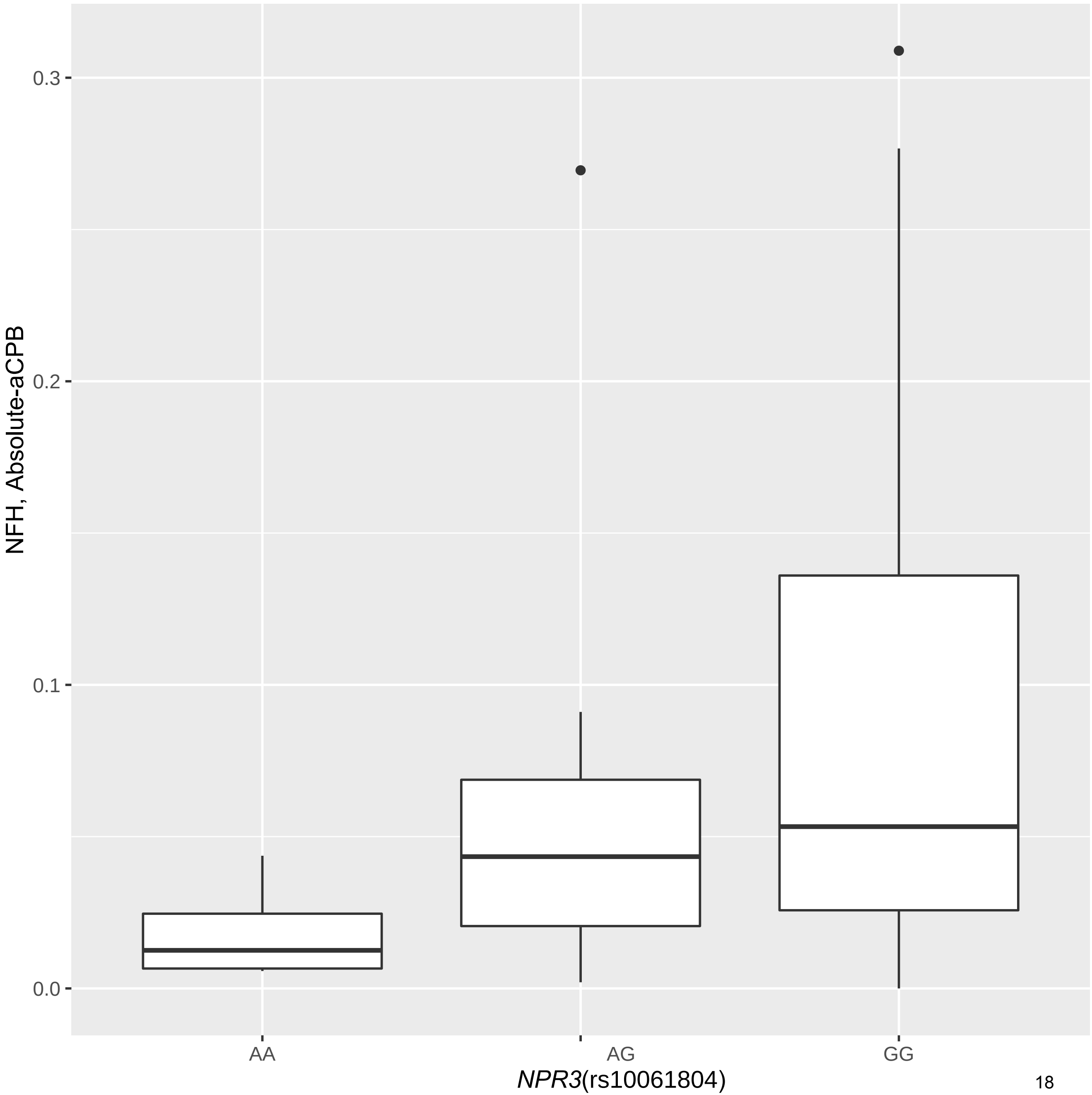
0.3
0.2
0.1
0.0
NFH, Absolute-aCPB
AA
AG
GG
NPR3(rs10061804)

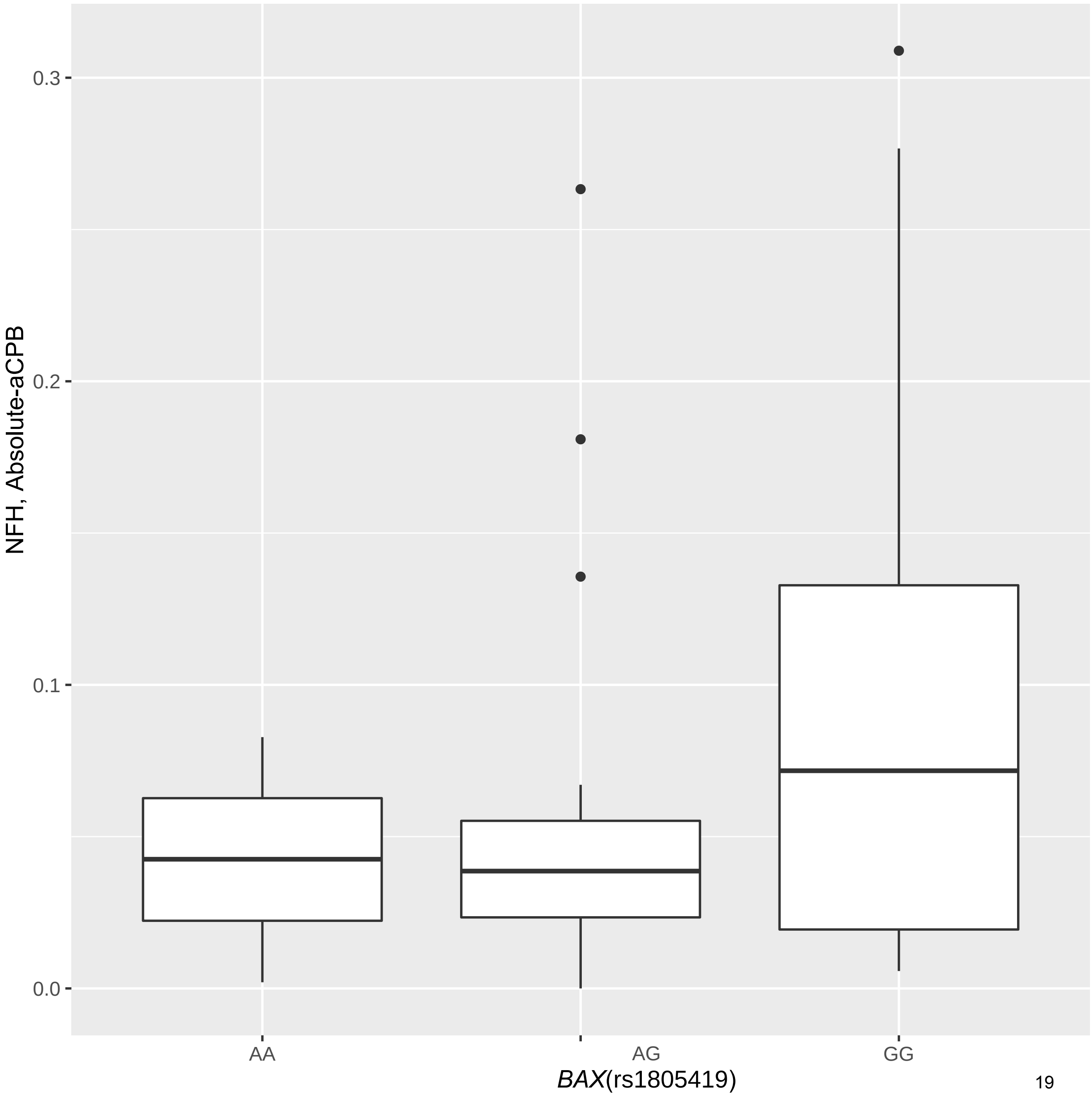
0.3
0.2
0.1
0.0
NFH, Absolute-aCPB
AA
AG
GG
BAX(rs1805419)

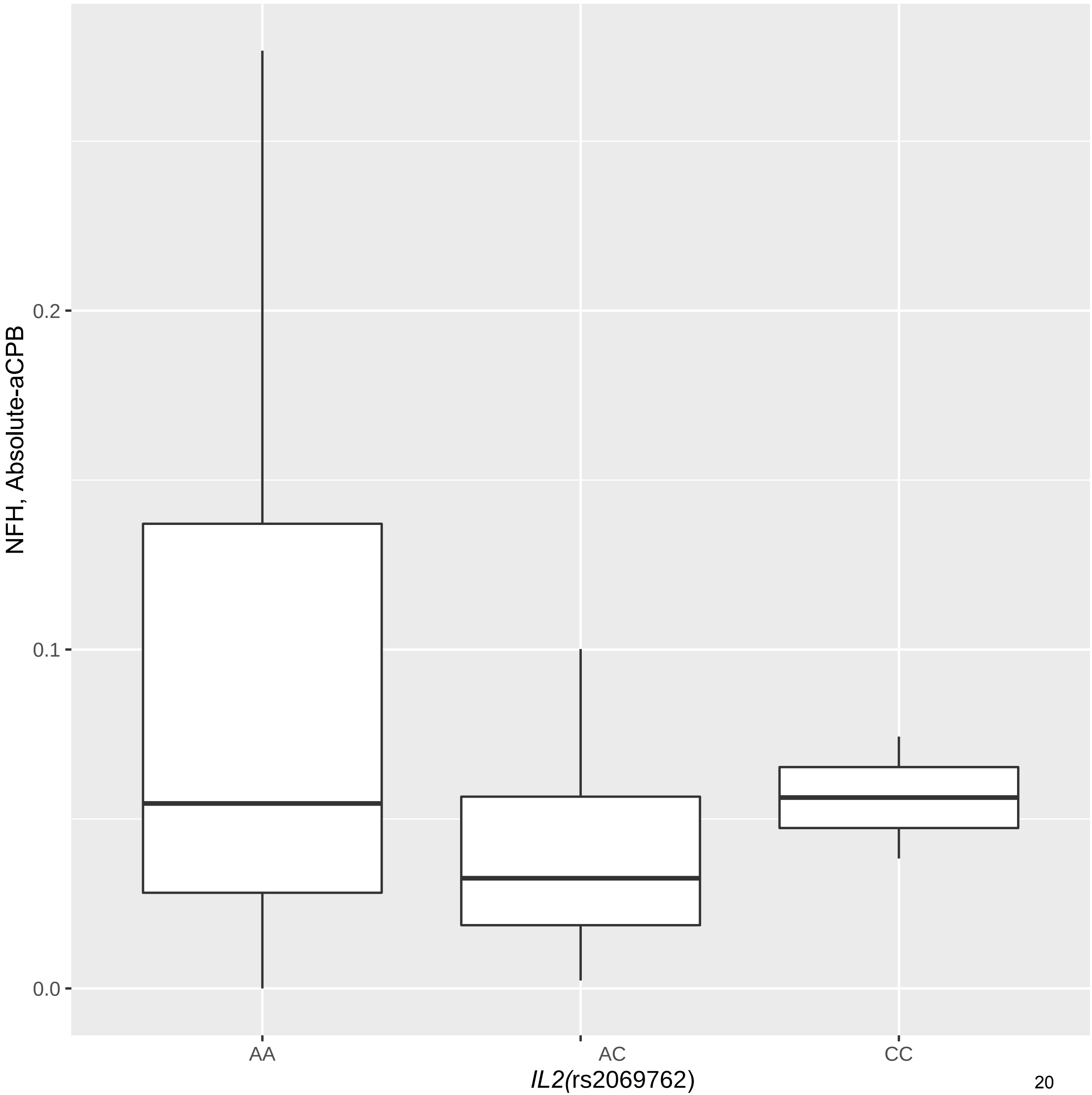
NFH, Absolute-aCPB
0.2
0.1
0.0
AA
AC
CC
IL2(rs2069762)

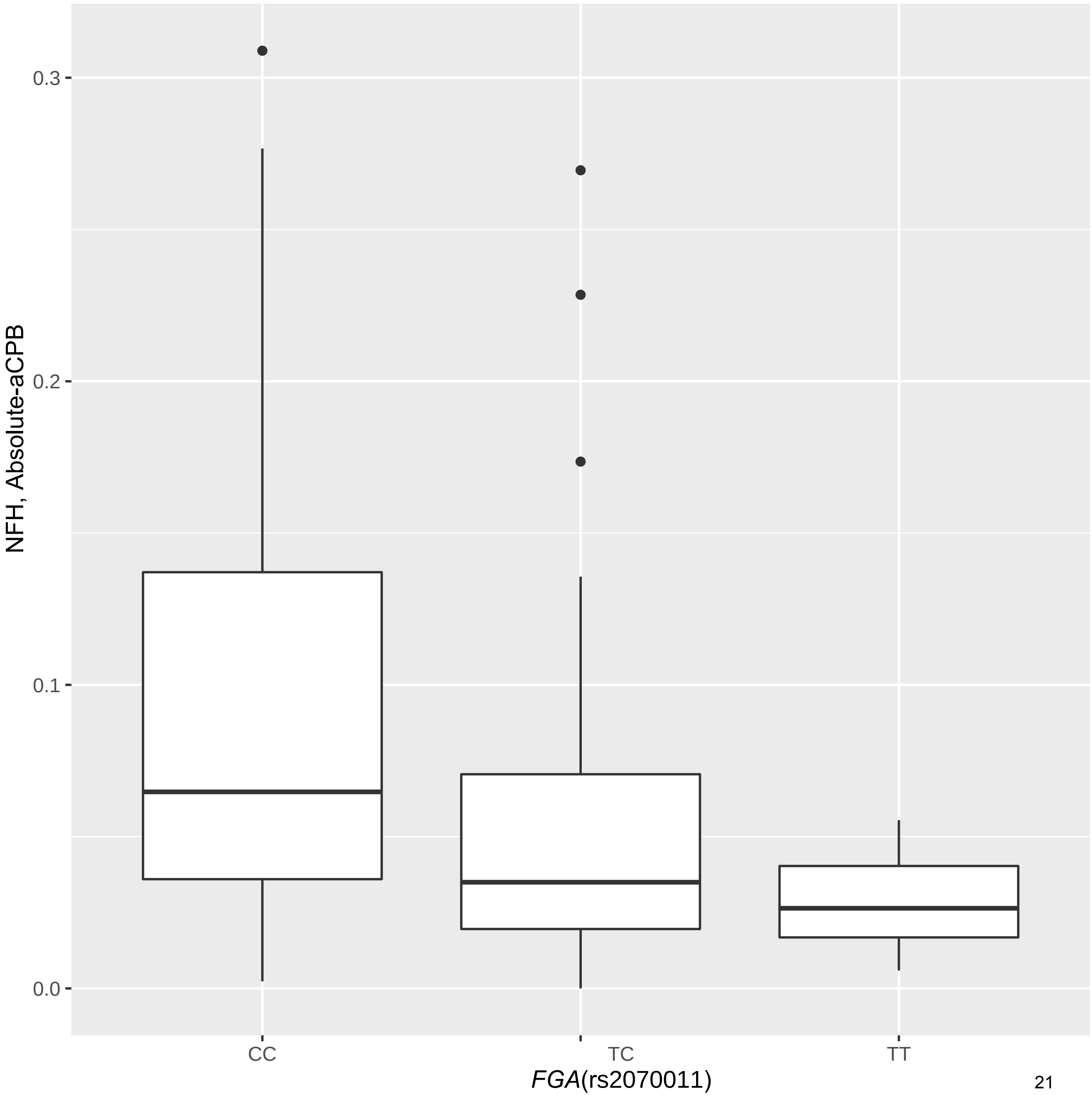

0.3
0.2
0.1
0.0
NFH, Absolute-aCPB
CC
TC
TT
FGA(rs2070011)

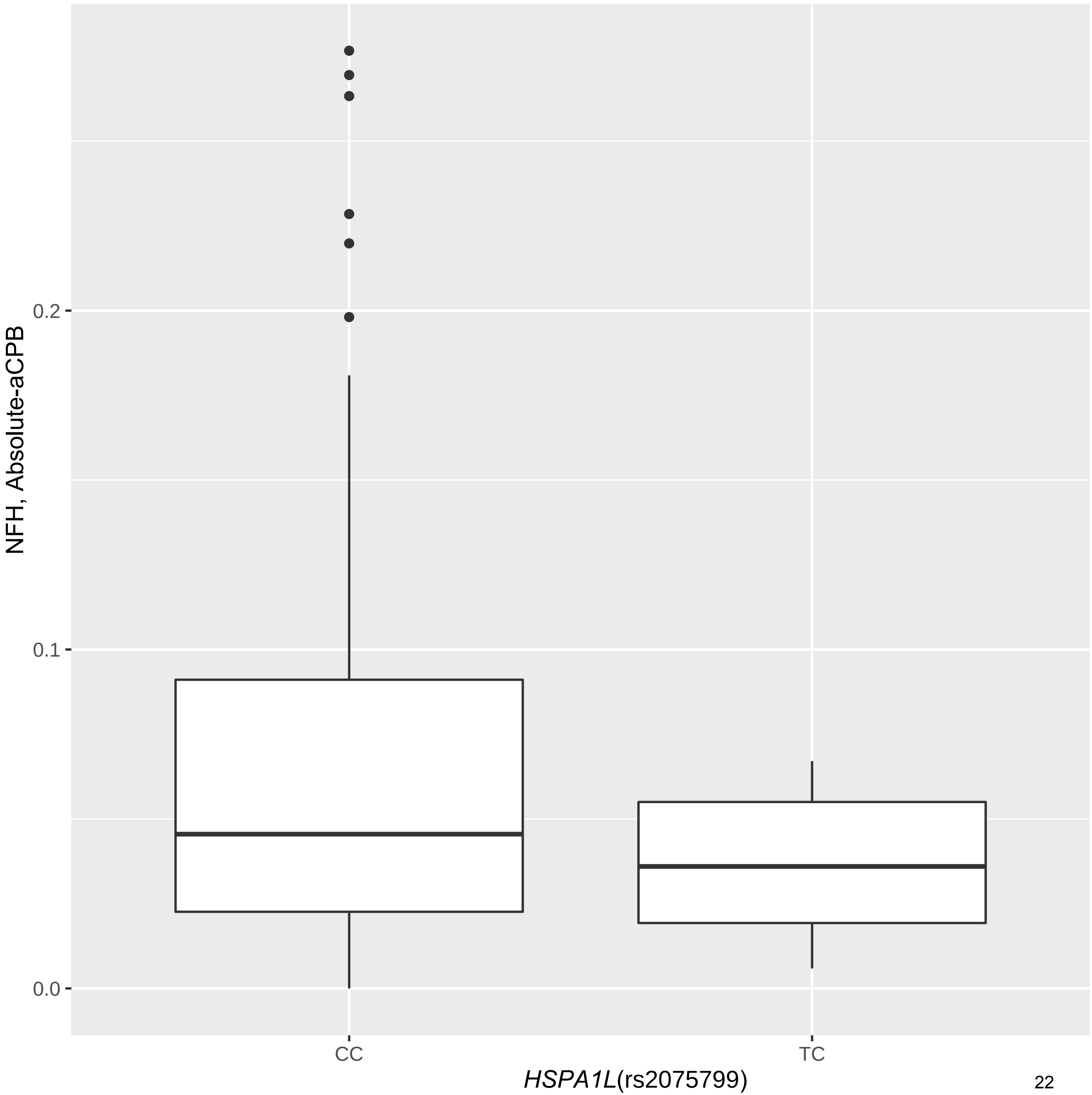
NFH, Absolute-aCPB
0.2
0.1
0.0
CC
TC
HSPA1L(rs2075799)

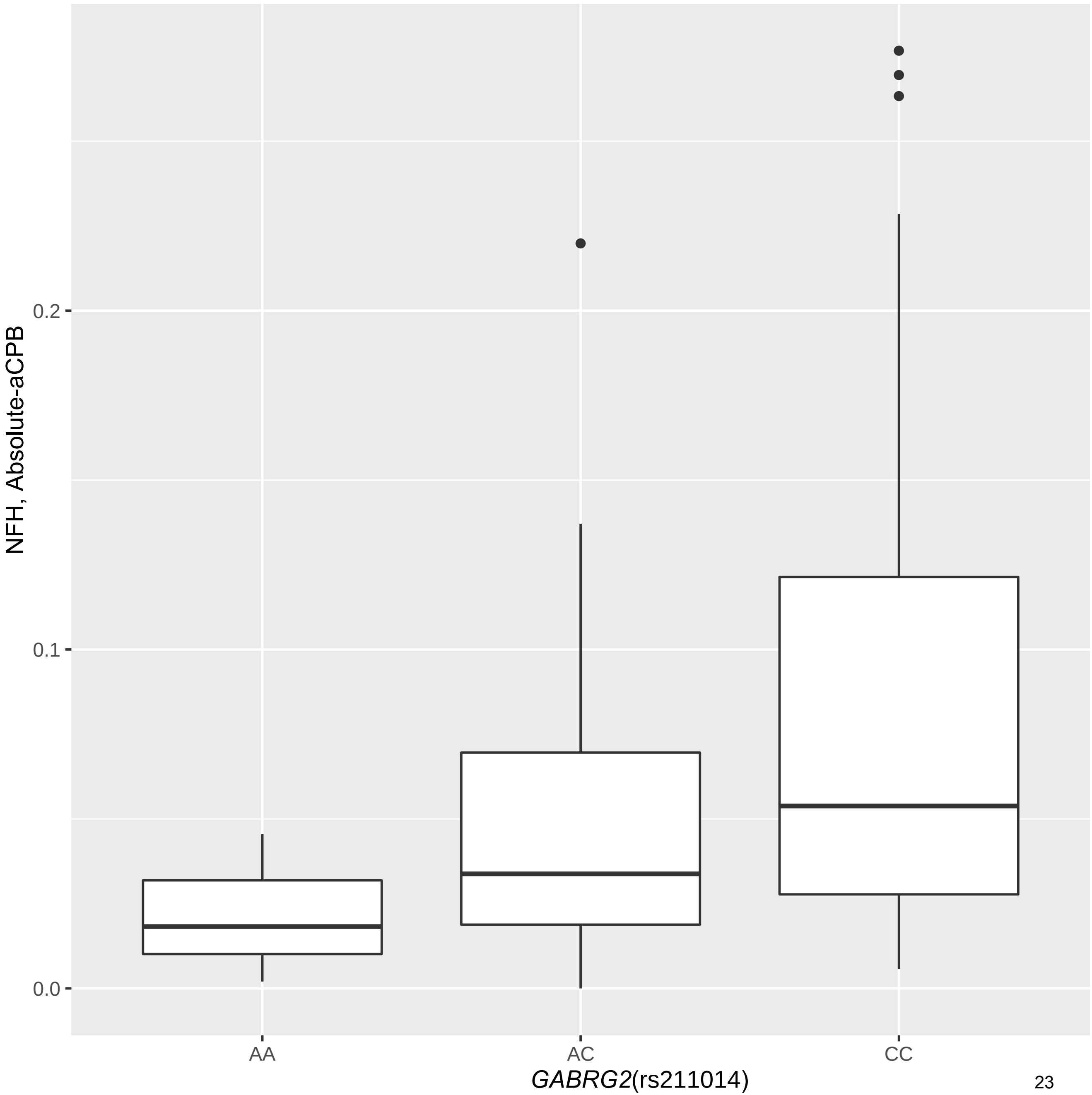
NFH, Absolute-aCPB
0.2
0.1
0.0
AA
AC
CC
GABRG2(rs211014)

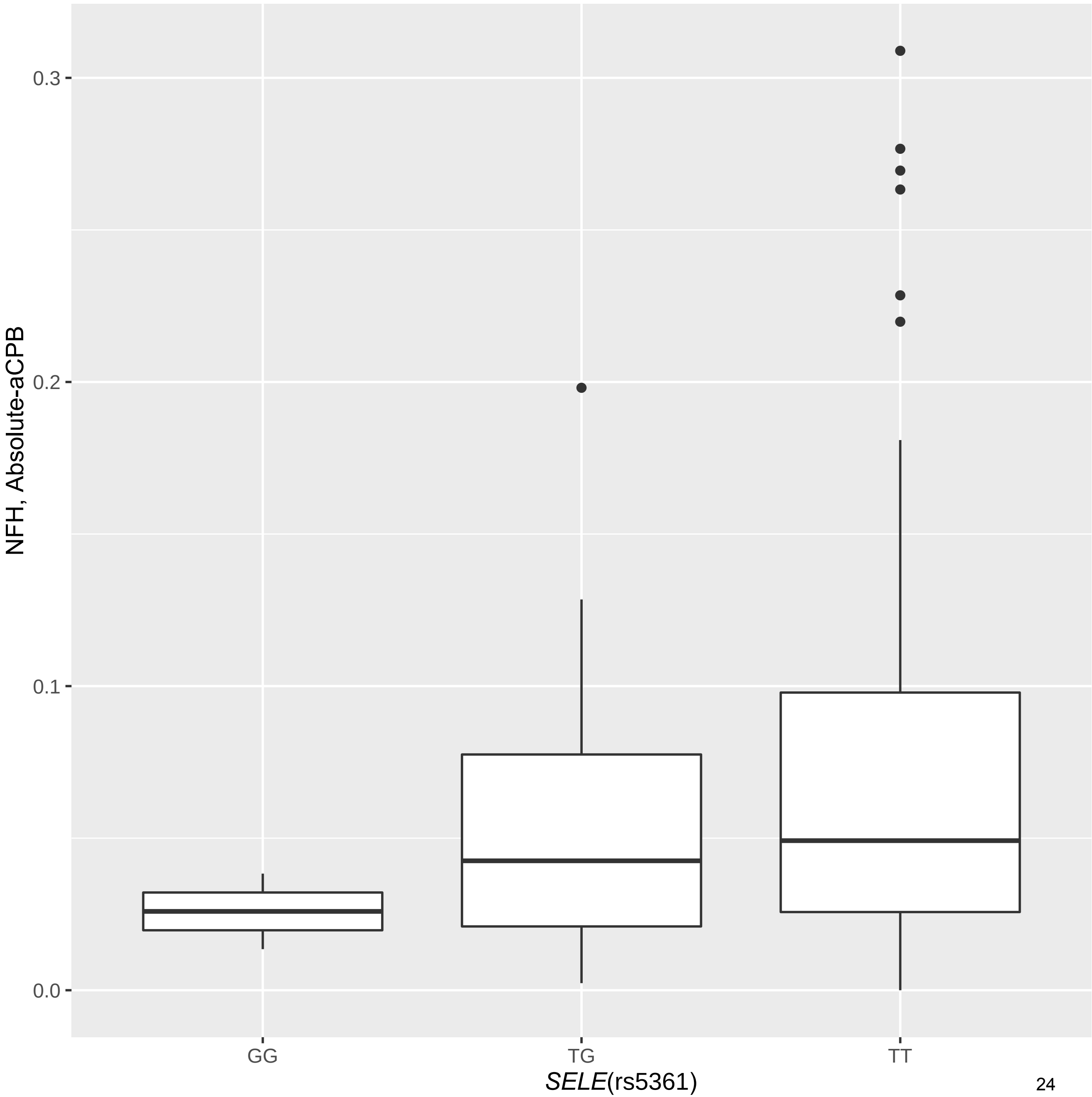

NFH, Absolute-aCPB
0.3
0.2
0.1
0.0
GG
TG
TT
SELE(rs5361)

NFH Absolute Values Associations-pCPB (NFH5)

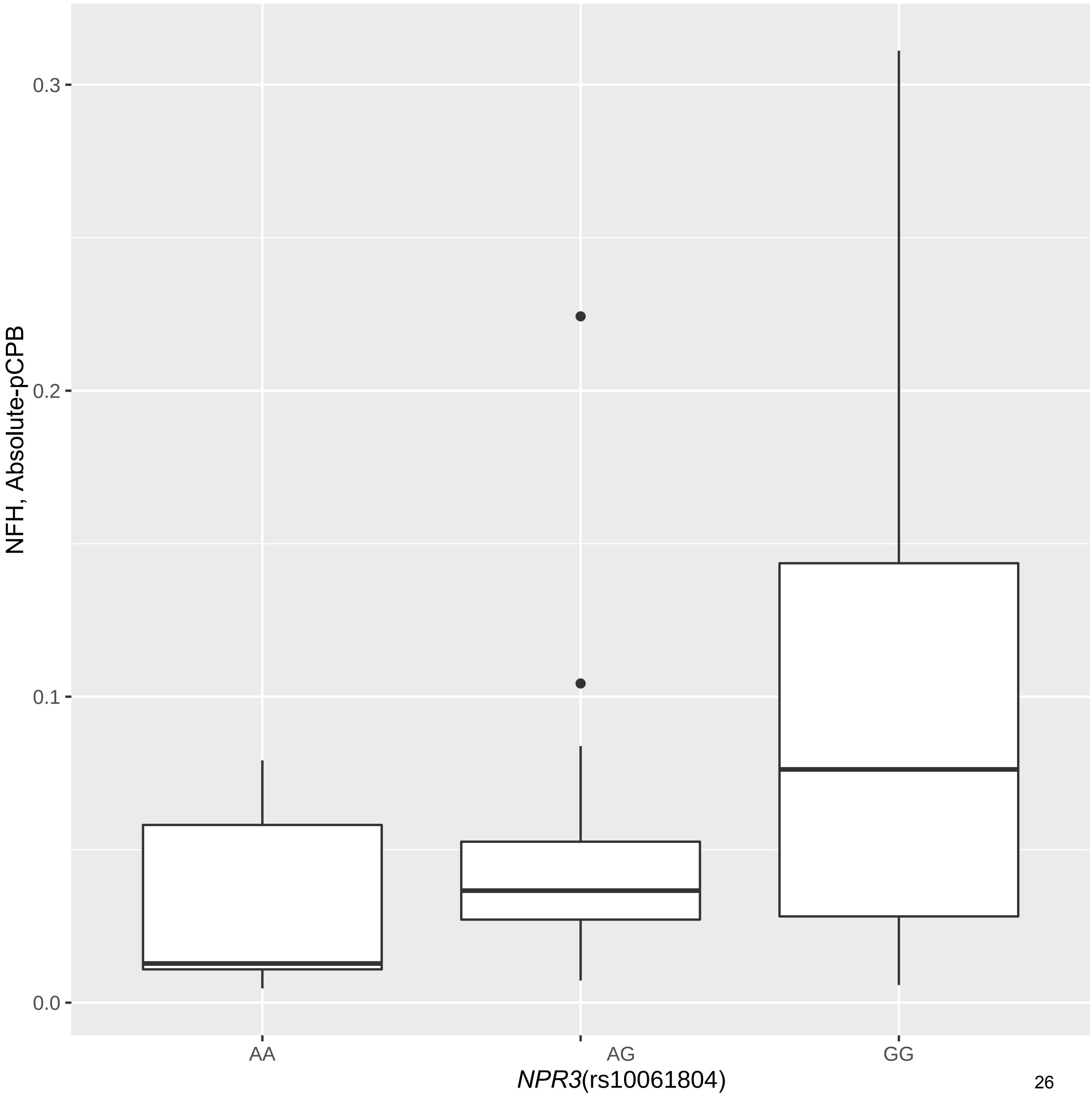
NFH, Absolute-pCPB
0.3
0.2
0.1
0.0
AA
AG
GG
NPR3(rs10061804)

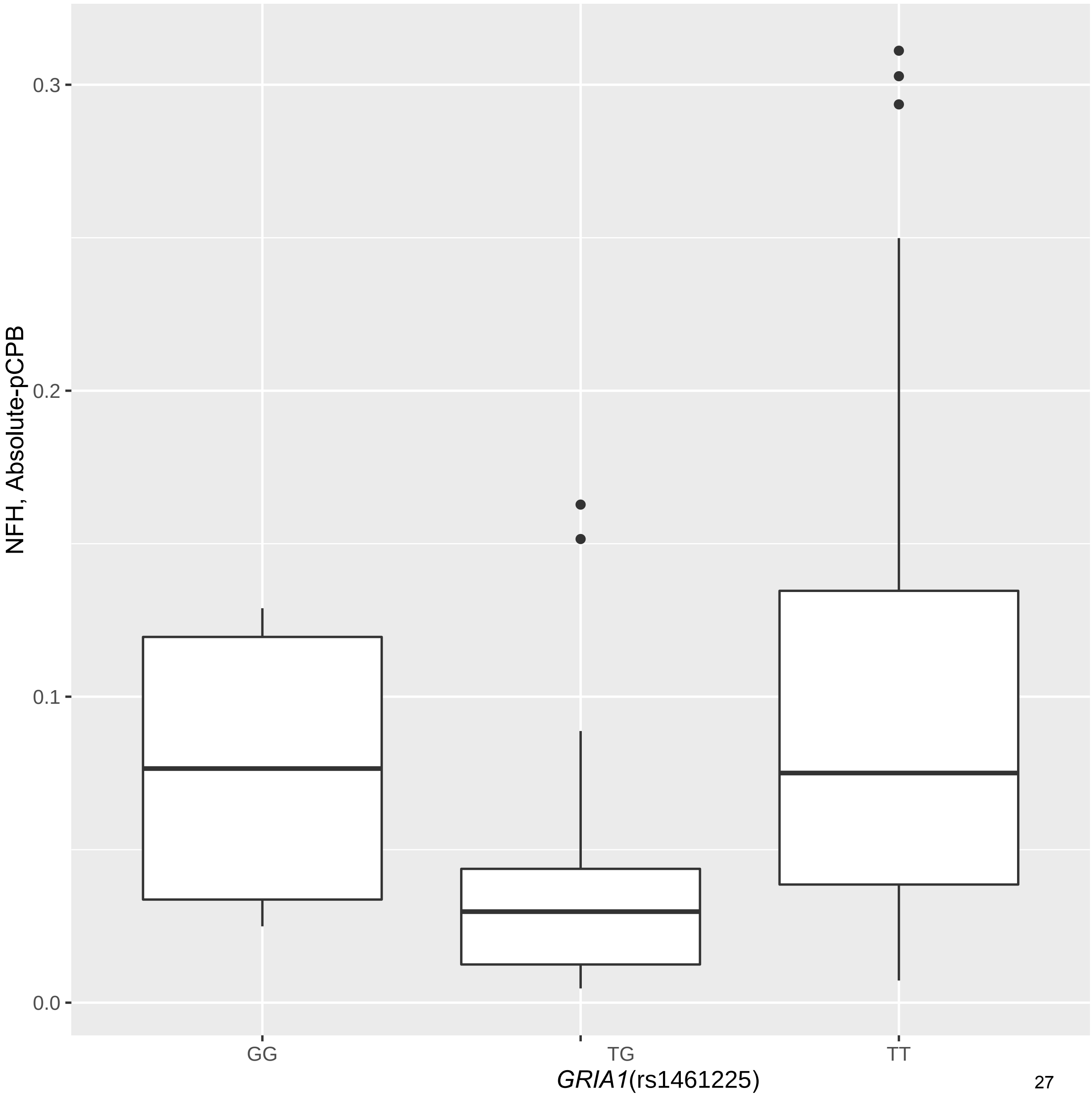

NFH, Absolute-pCPB
0.3
0.2
0.1
0.0
GG
TG
TT
GRIA1(rs1461225)

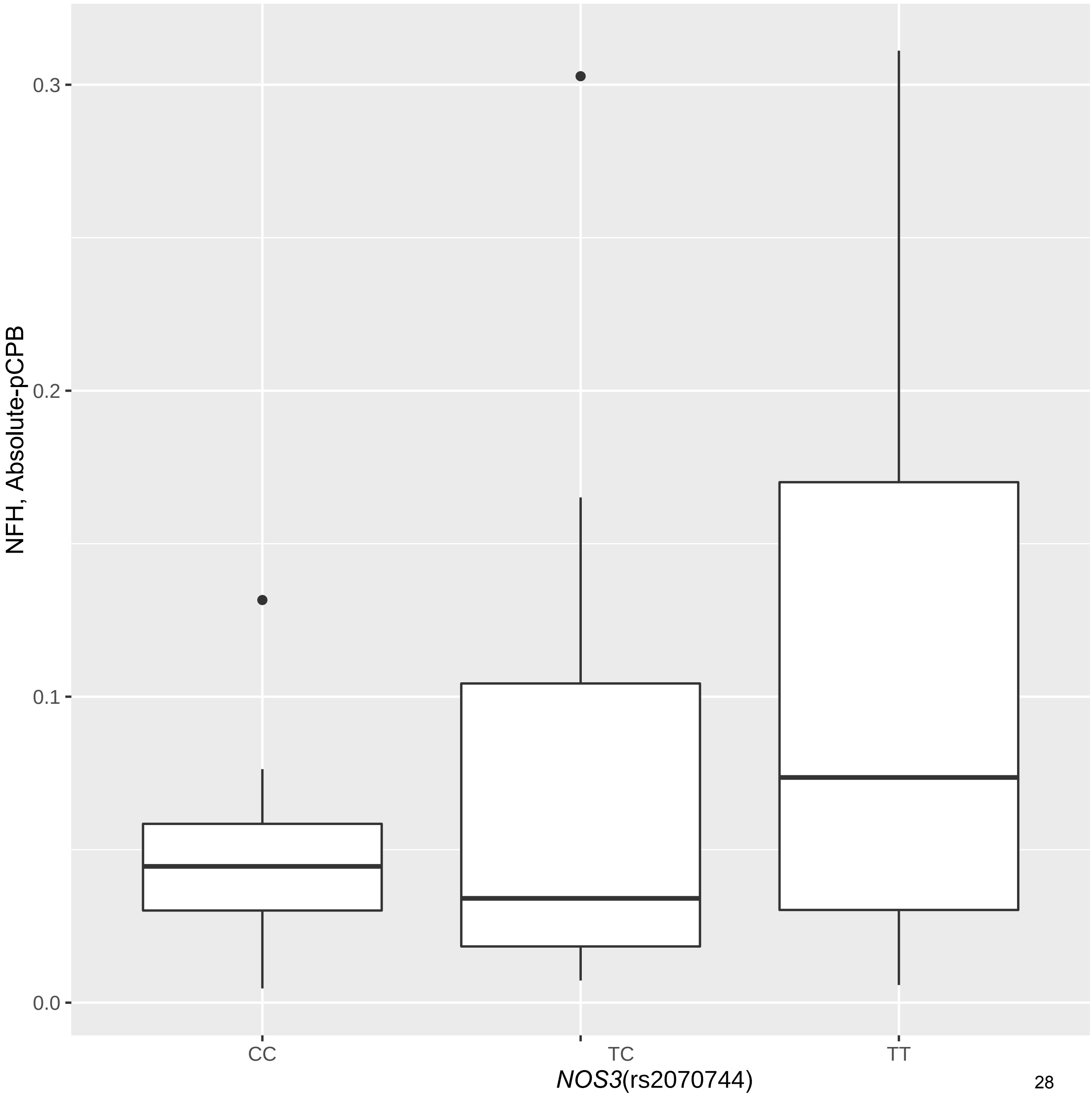
NFH, Absolute-pCPB
0.3
0.2
0.1
0.0
CC
TC
TT
NOS3(rs2070744)

NFH Absolute Values Associations-24H (NFH6)

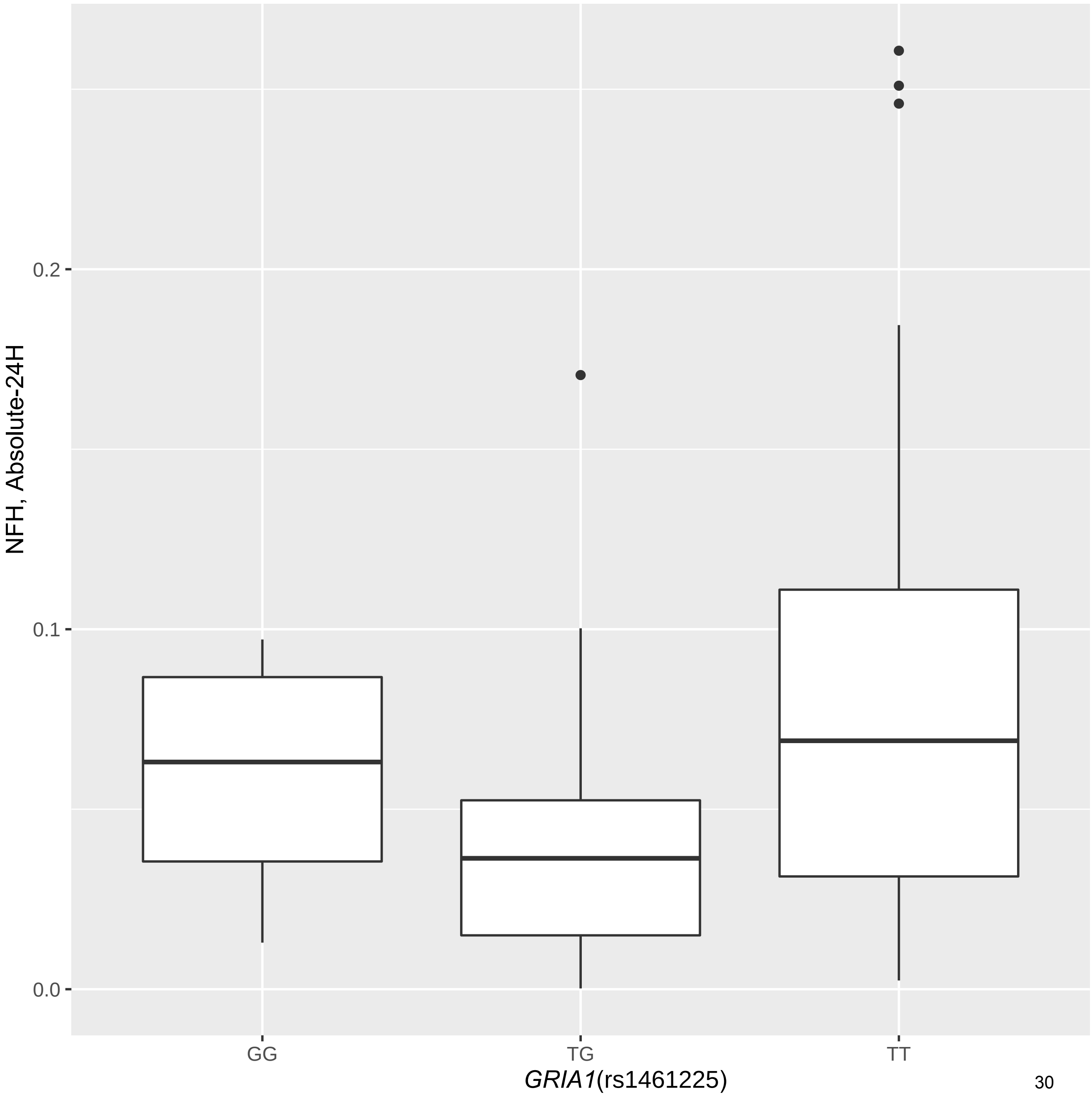

NFH, Absolute-24H
0.2
0.1
0.0
GG
TG
TT
GRIA1(rs1461225)

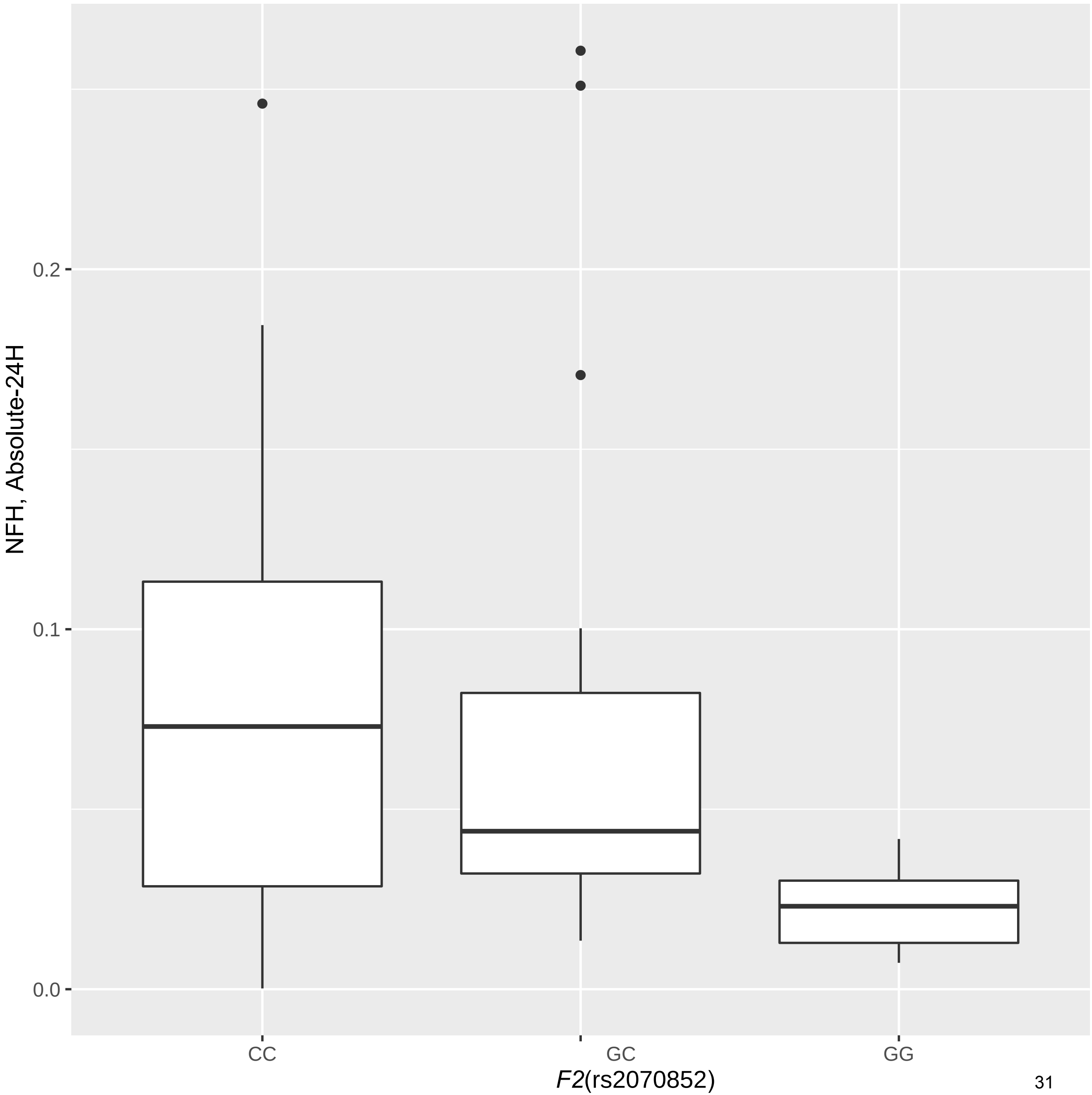

0.2
0.1
0.0
NFH, Absolute-24H
CC
GC
GG
F2(rs2070852)

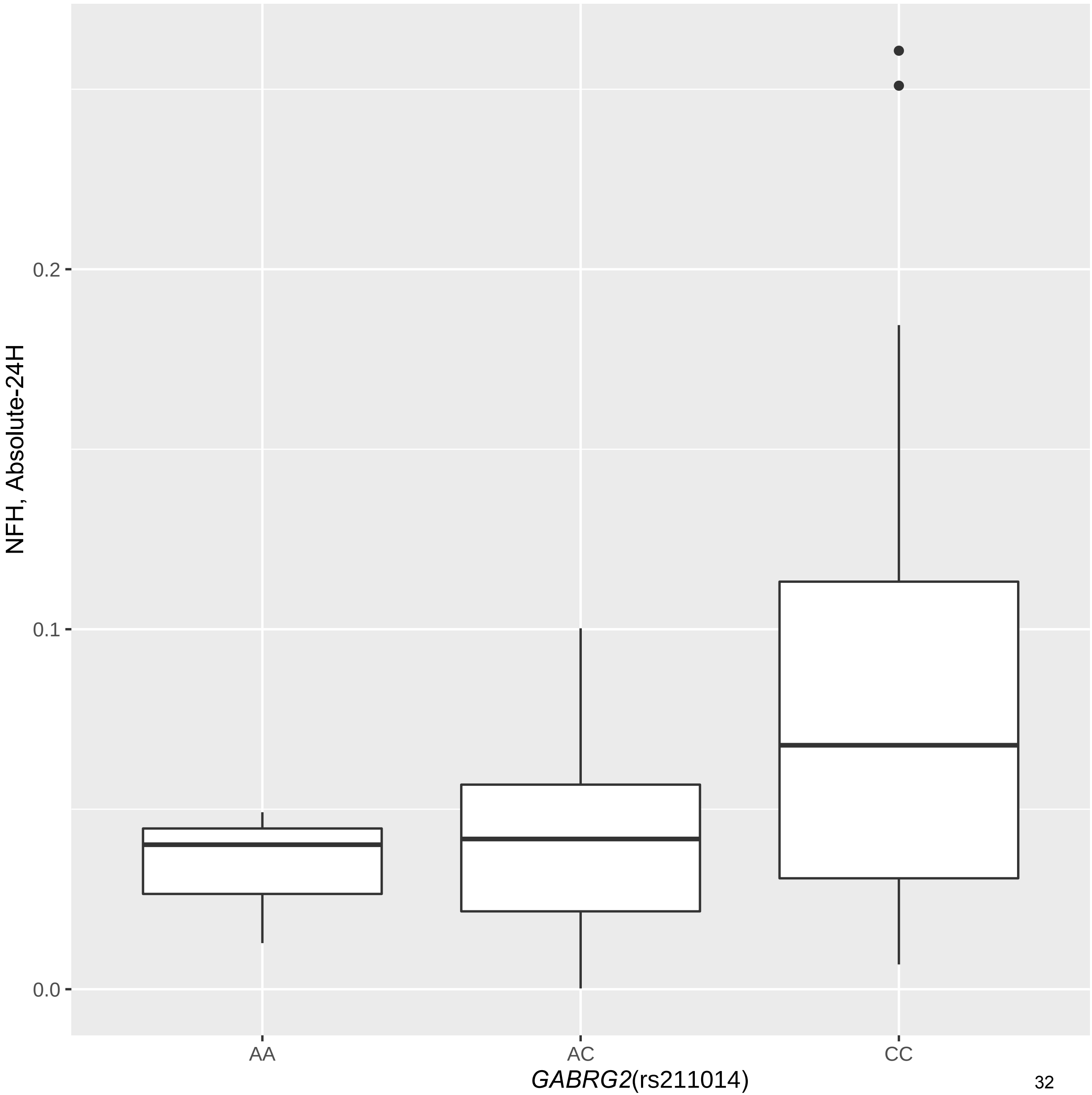

NFH, Absolute-24H
0.2
0.1
0.0
AA
AC
CC
GABRG2(rs211014)

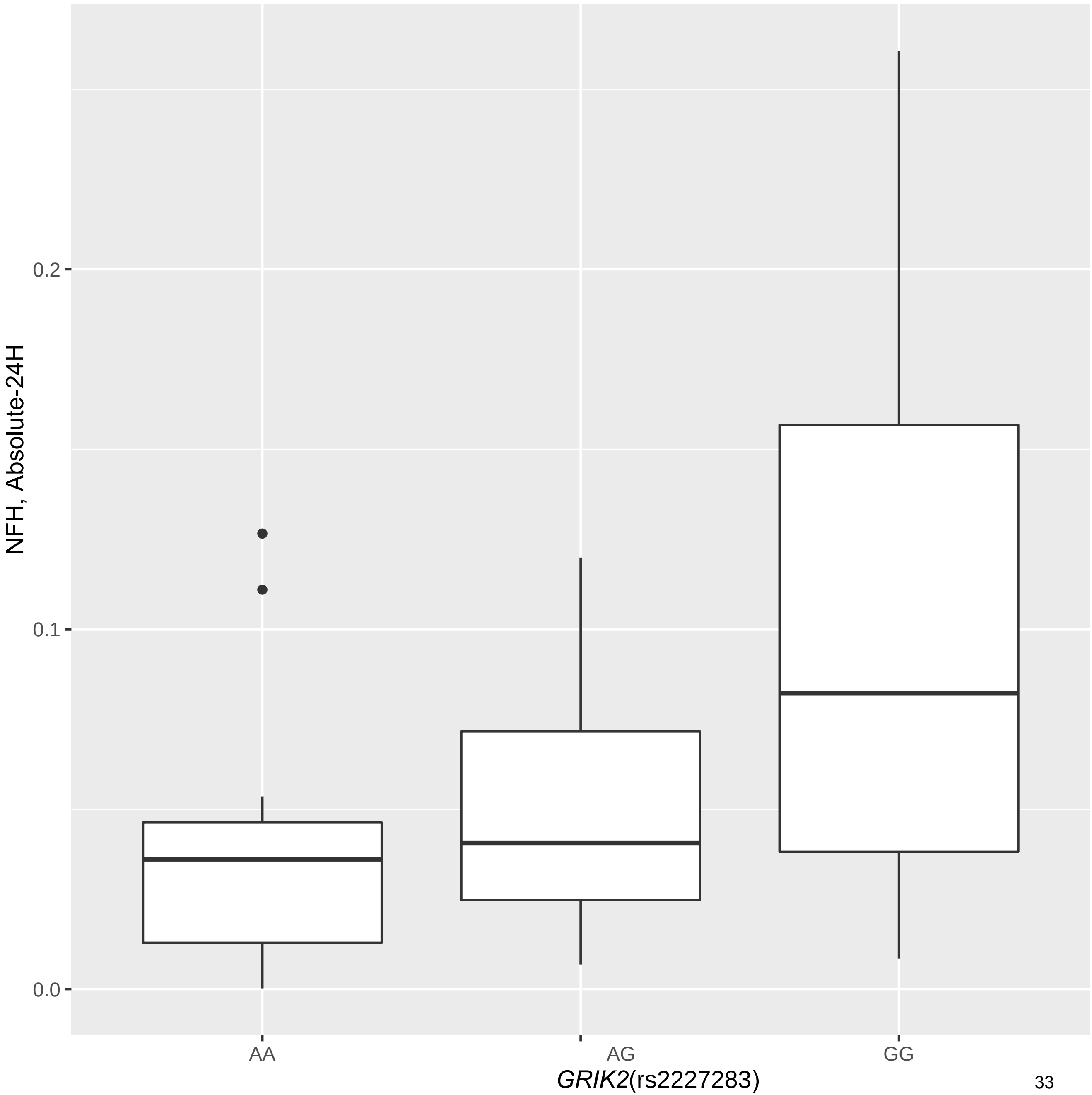
NFH, Absolute-24H
0.2
0.1
0.0
AA
AG
GG
GRIK2(rs2227283)

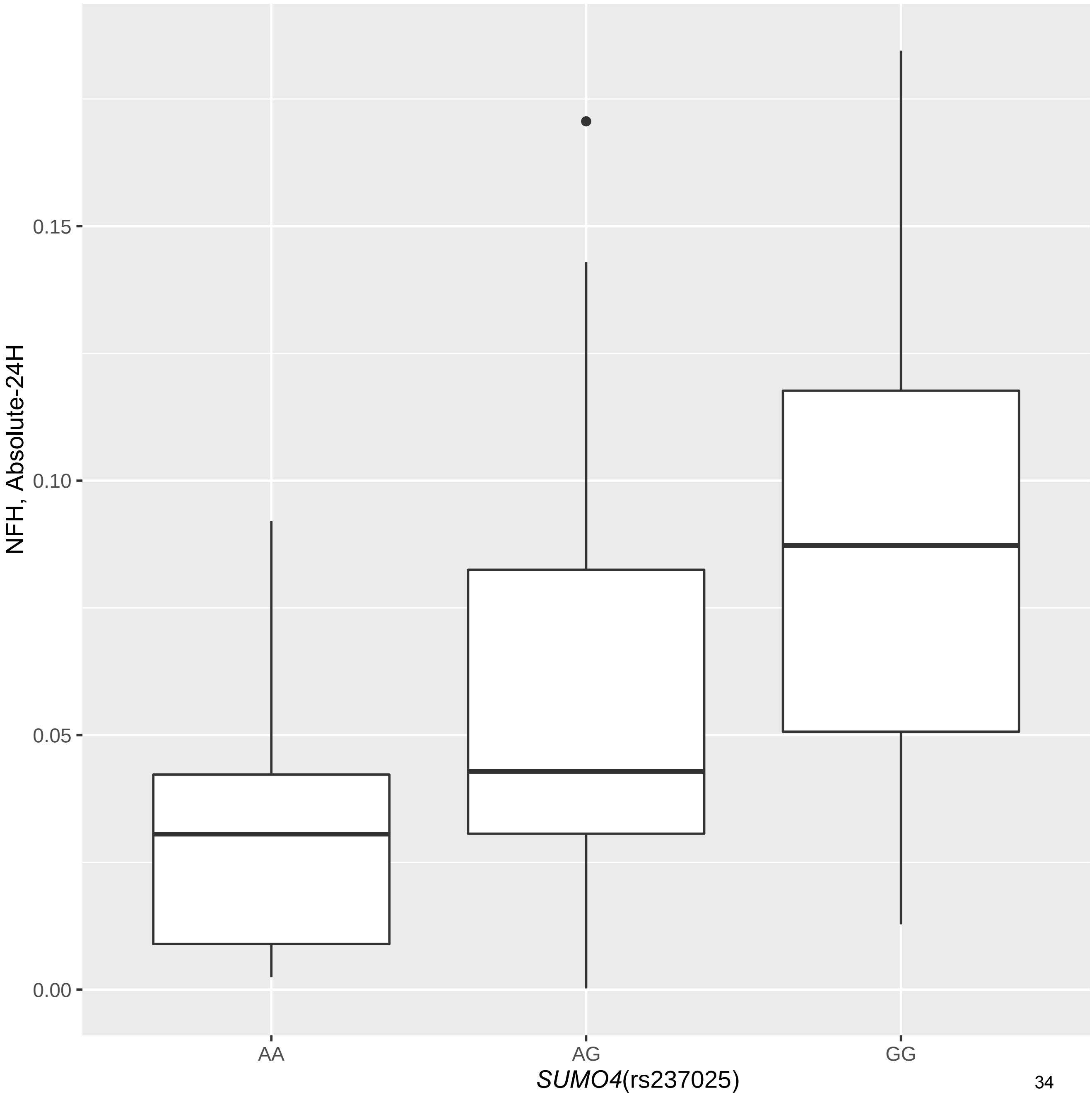

NFH, Absolute-24H
0.15
0.10
0.05
0.00
AA
AG
GG
SUMO4(rs237025)

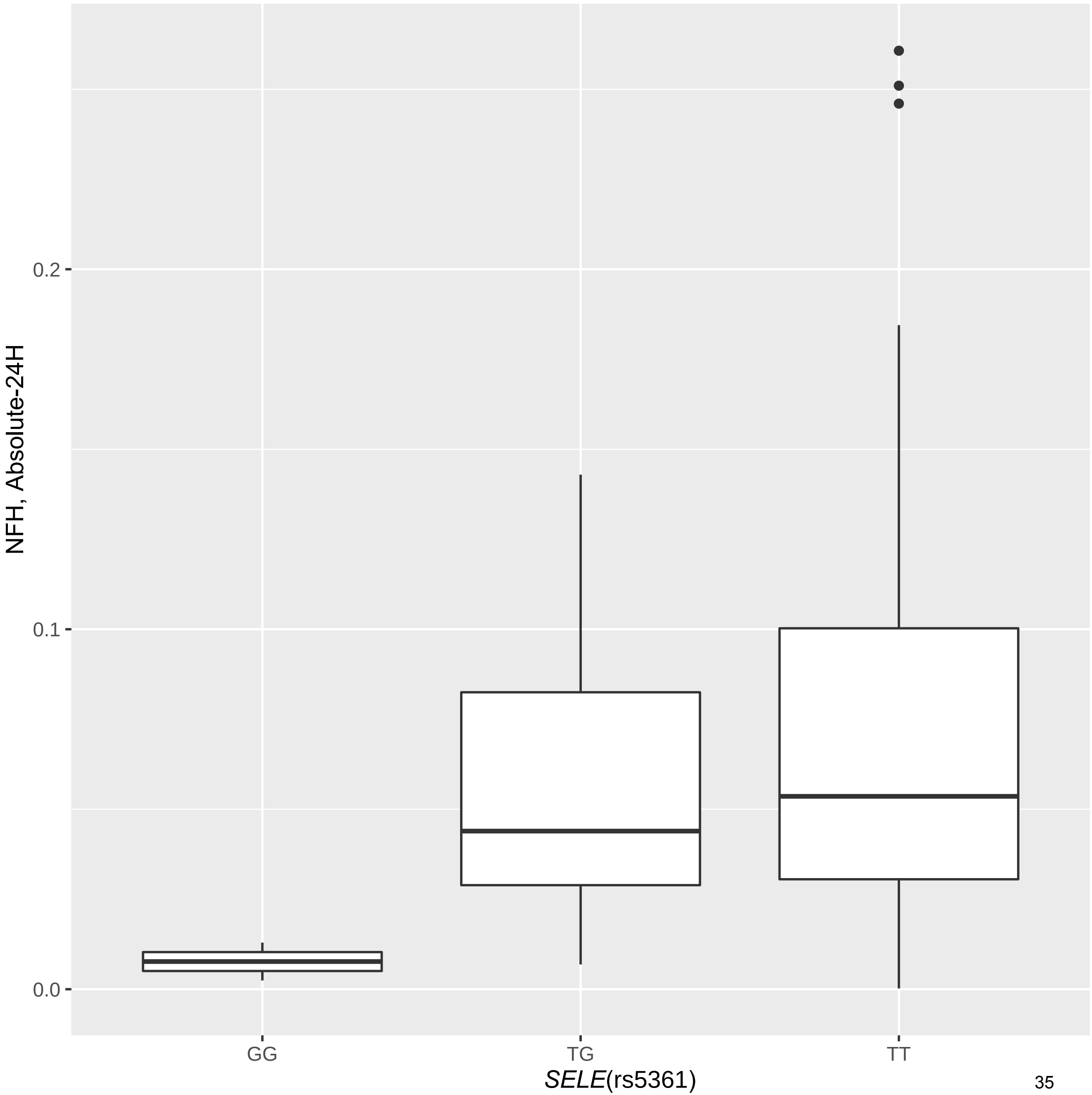
NFH, Absolute-24H
0.2
0.1
0.0
GG
TG
TT
SELE(rs5361)

S100β Absolute Values Associations-BL (S100B 1)

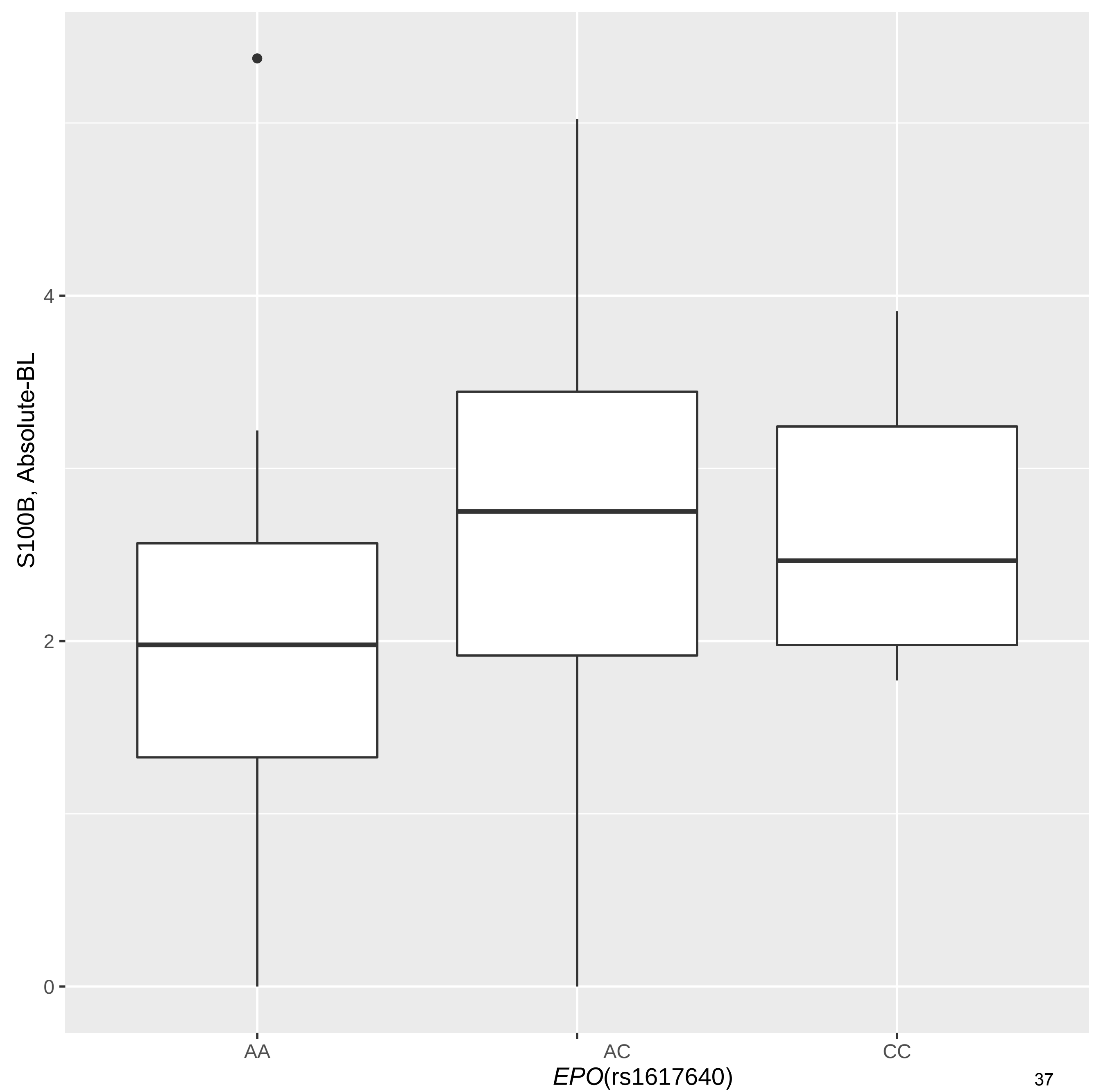

S100B, Absolute-BL
0
2
4
AA
AC
CC
EPO(rs1617640)

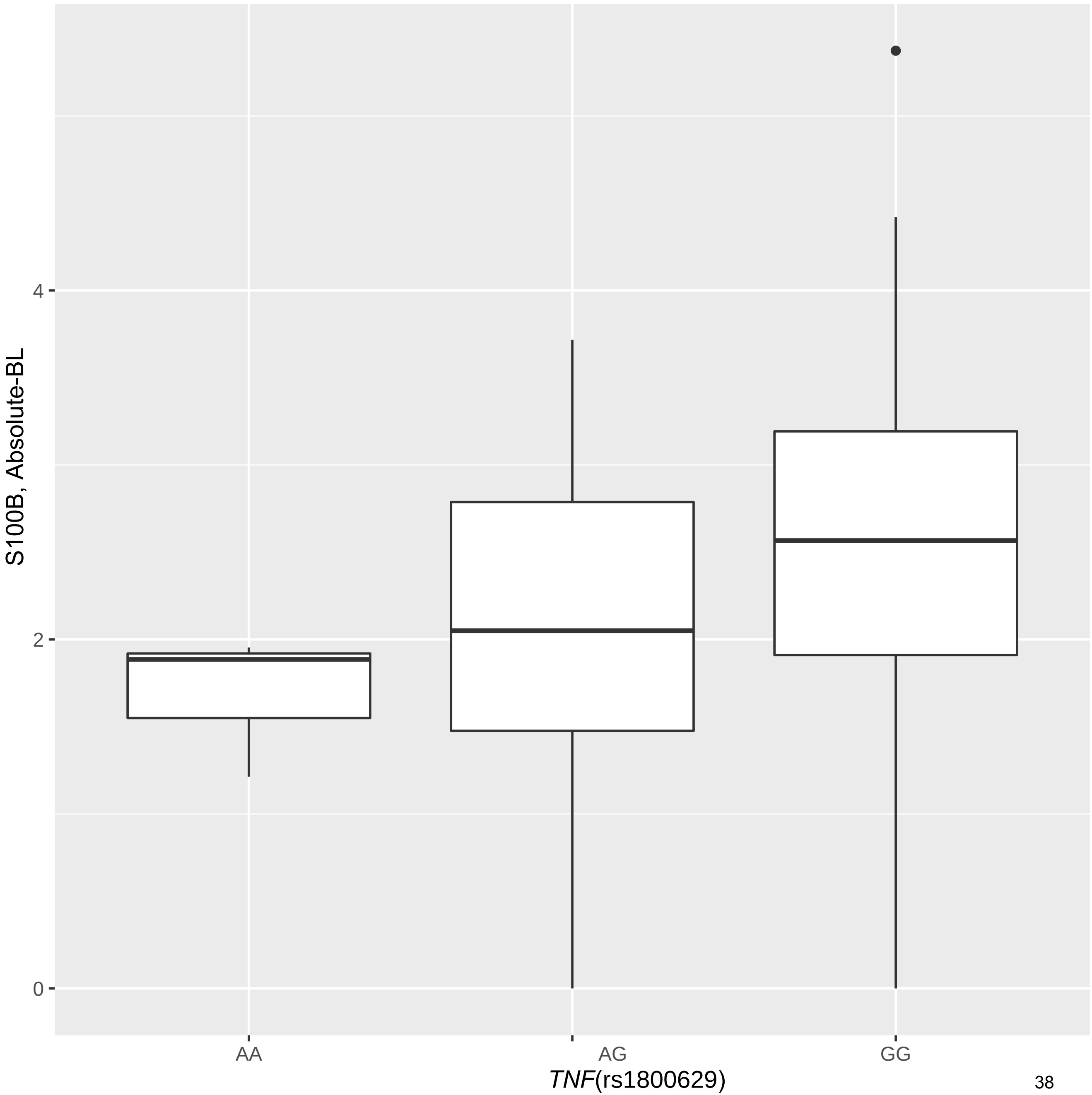

S100B, Absolute-BL
4
2
0
AA
AG
GG
TNF(rs1800629)

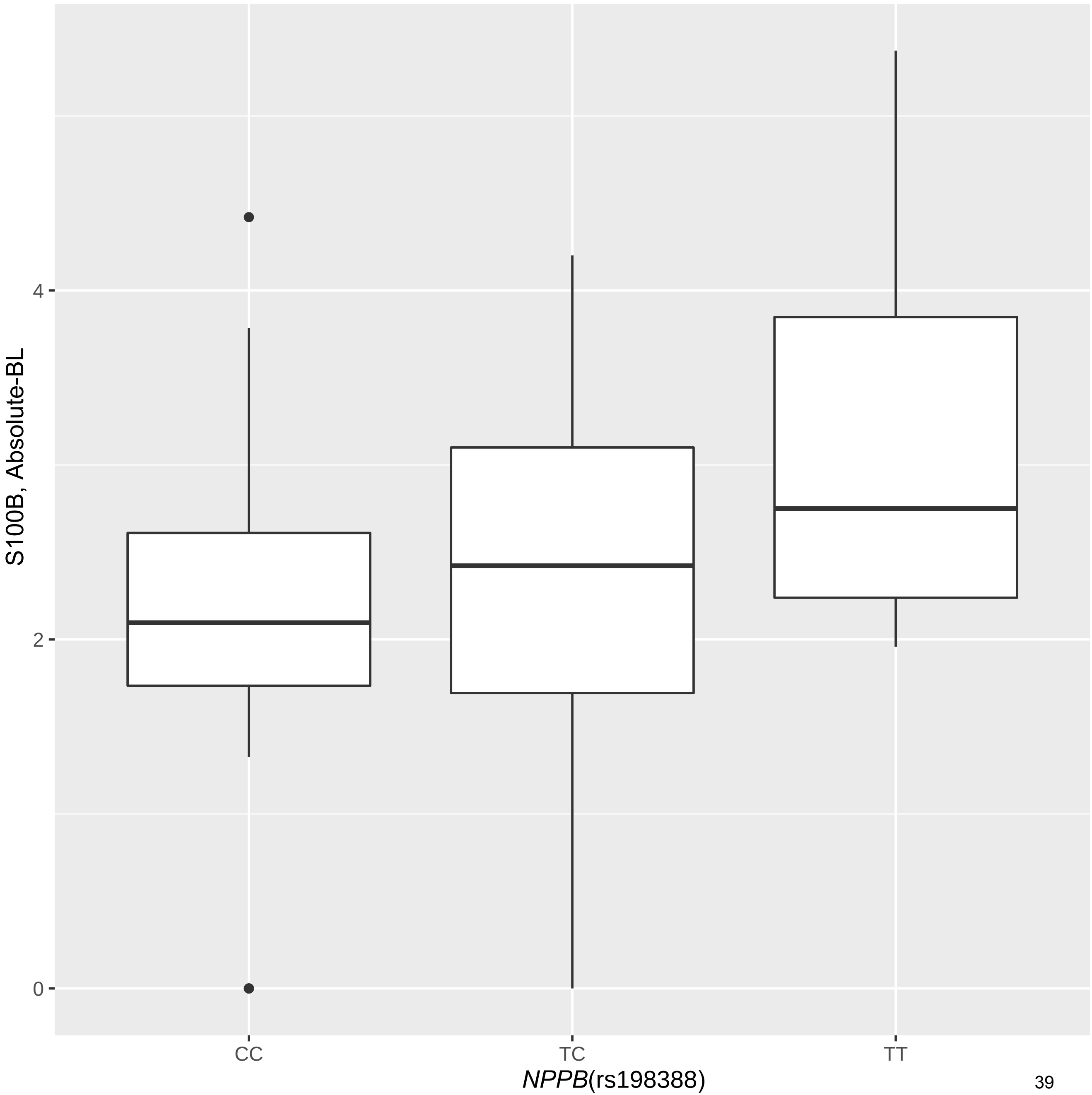

S100B, Absolute-BL
0
2
4
CC
TC
TT
NPPB(rs198388)

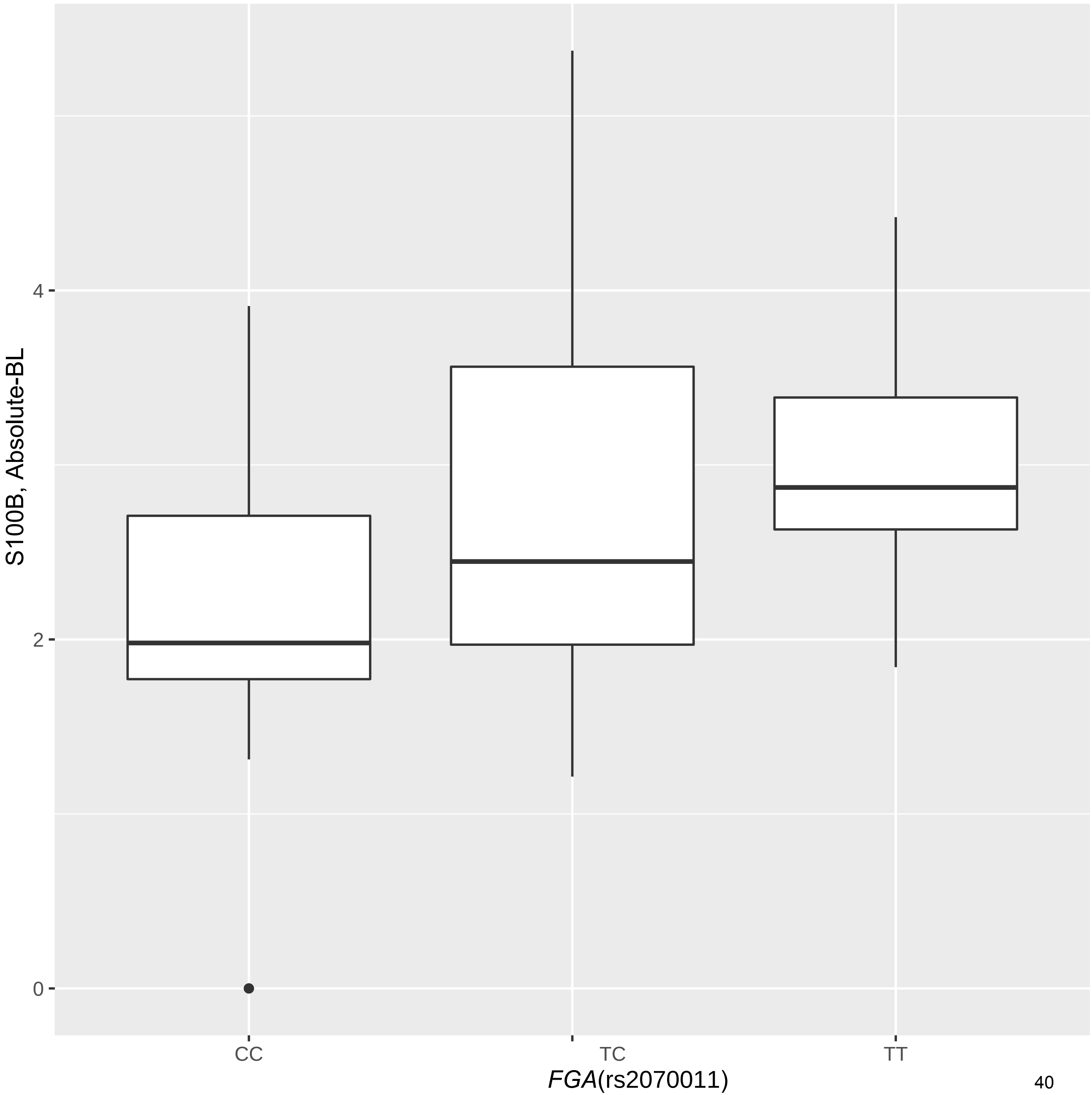
S100B, Absolute-BL
4
2
0
CC
TC
TT
FGA(rs2070011)

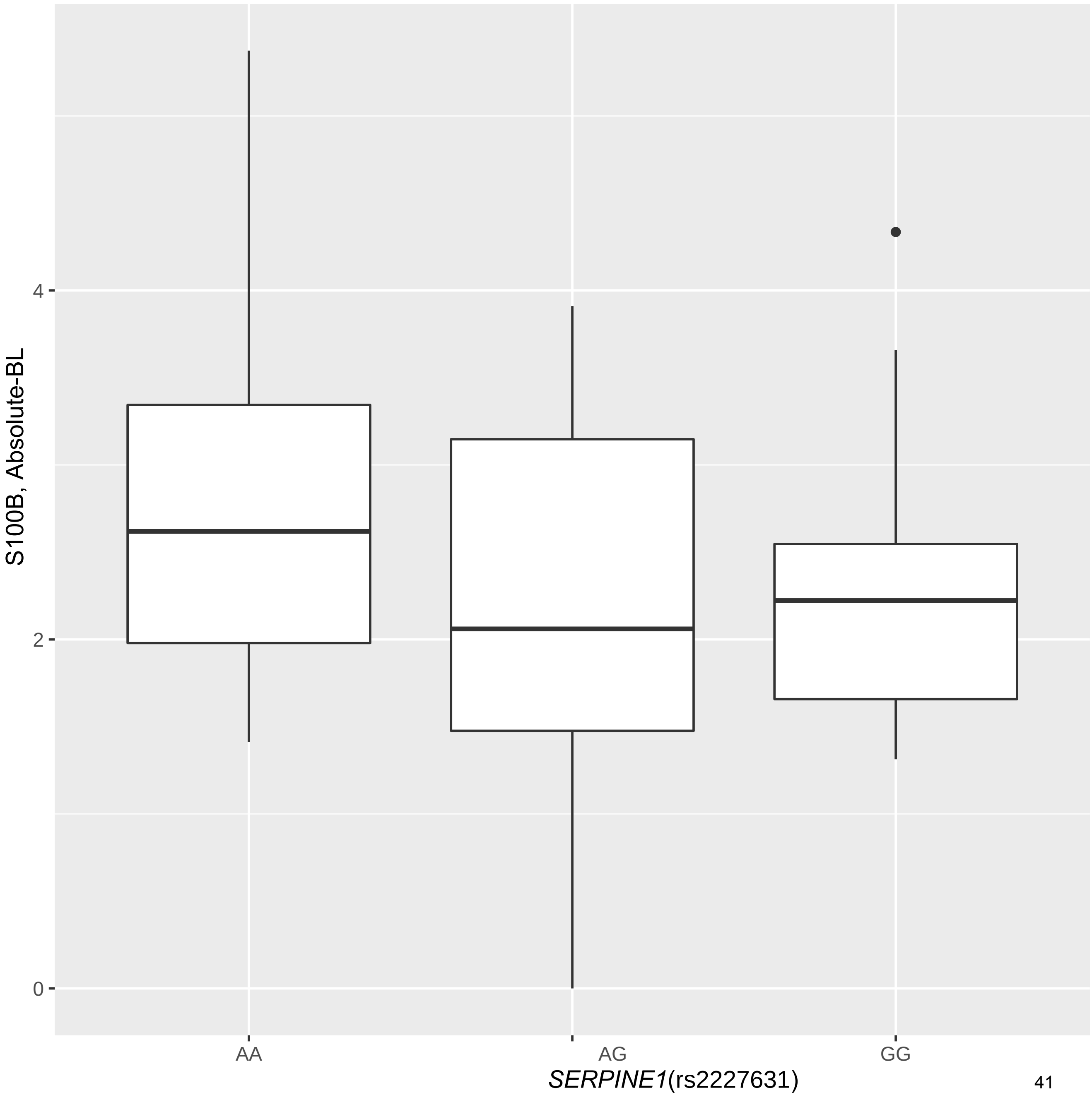
S100B, Absolute-BL
4
2
0
AA
AG
GG
SERPINE1(rs2227631)

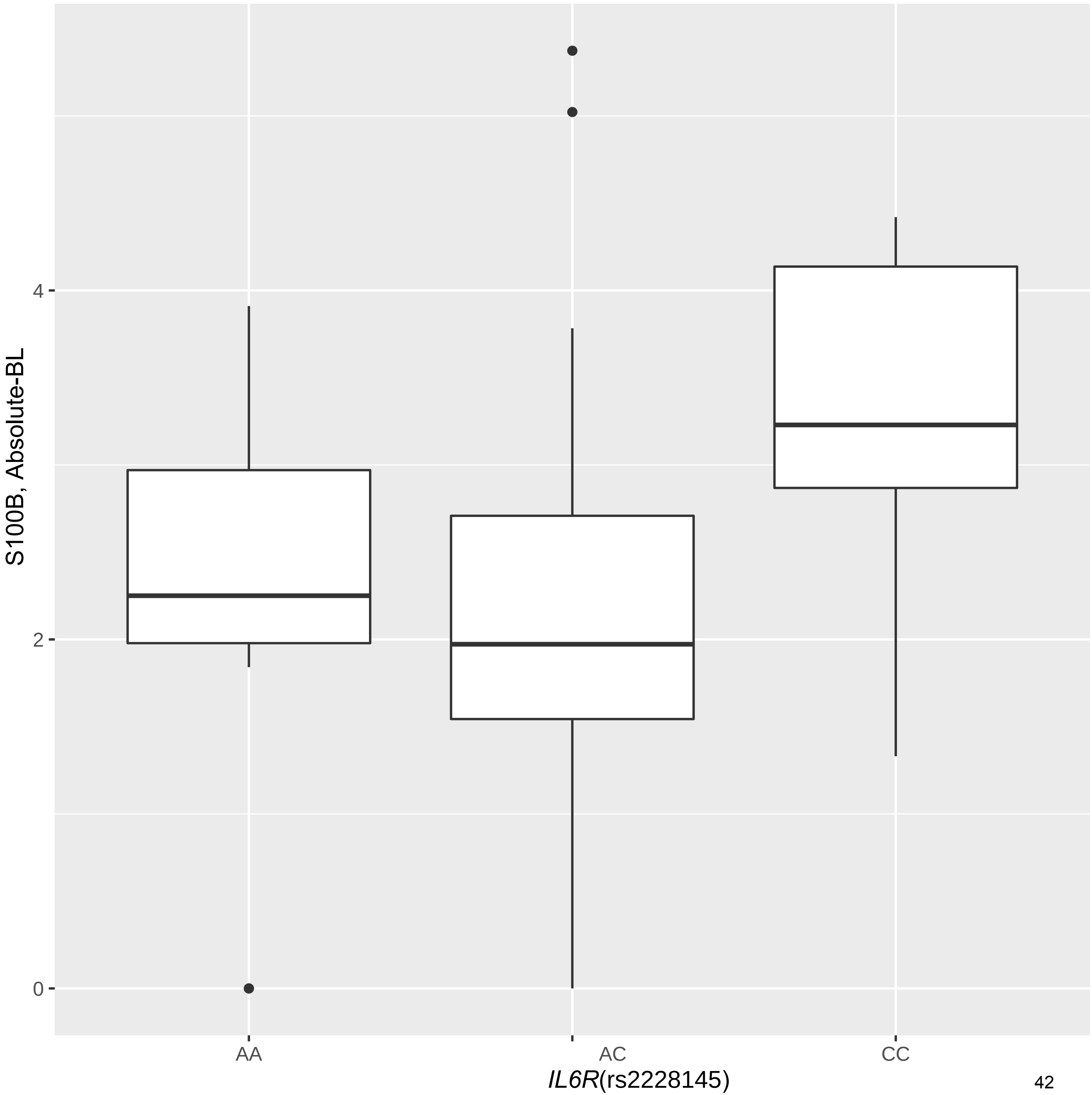

S100B, Absolute-BL
0
2
4
AA
AC
CC
IL6R(rs2228145)

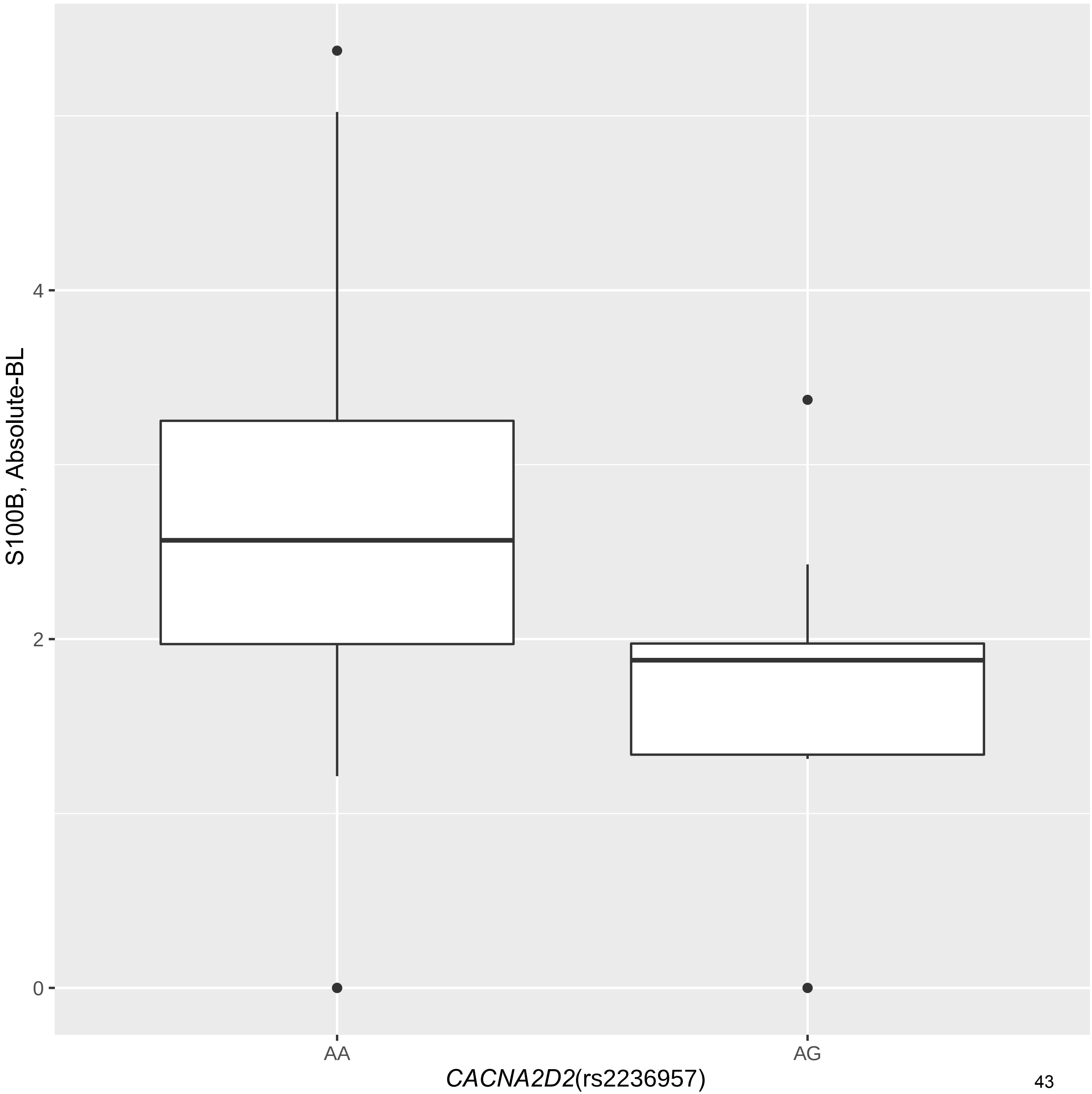

S100B, Absolute-BL
4
2
0
AA
AG
CACNA2D2(rs2236957)

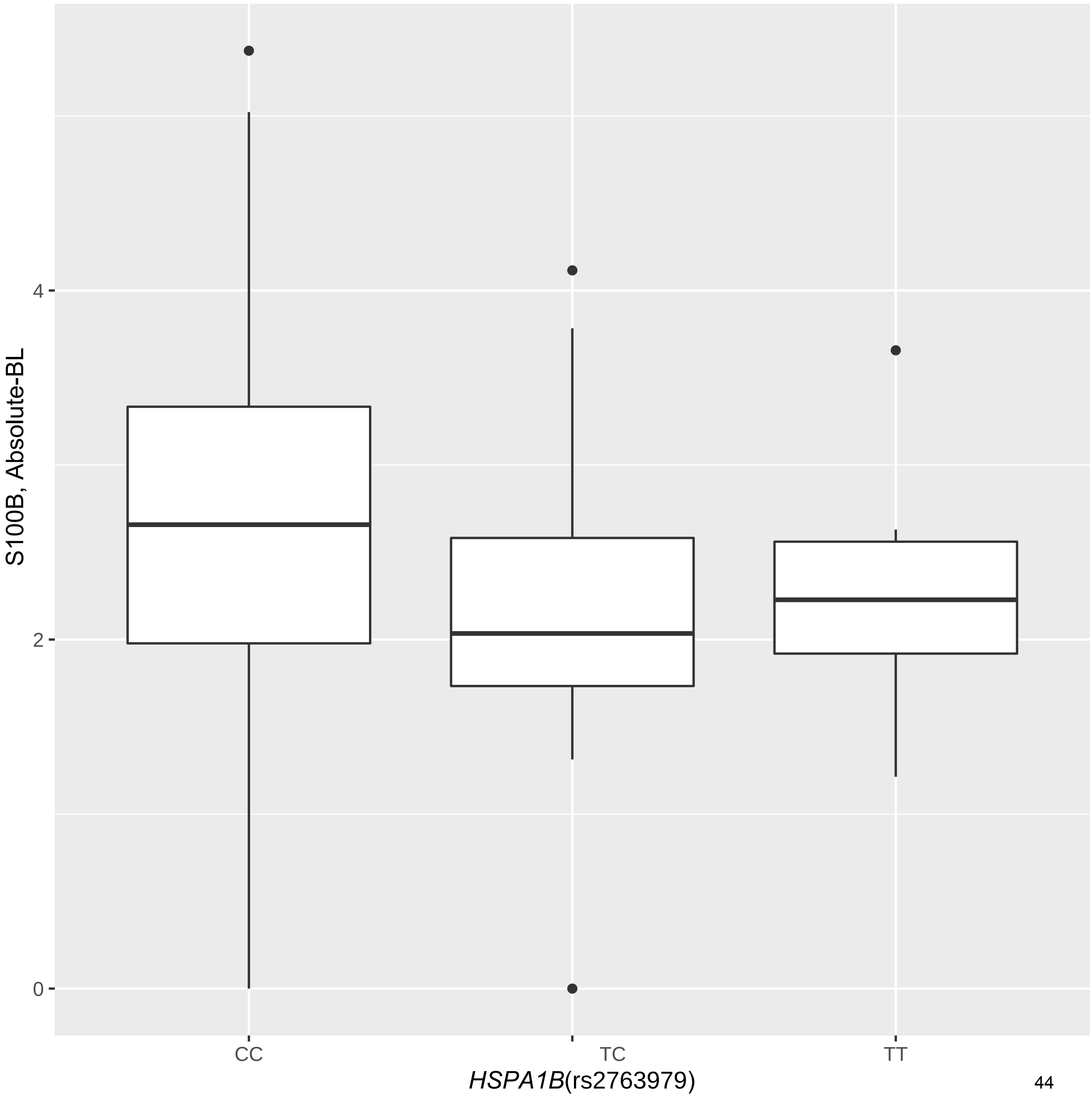
S100B, Absolute-BL
4
2
0
CC
TC
TT
HSPA1B(rs2763979)

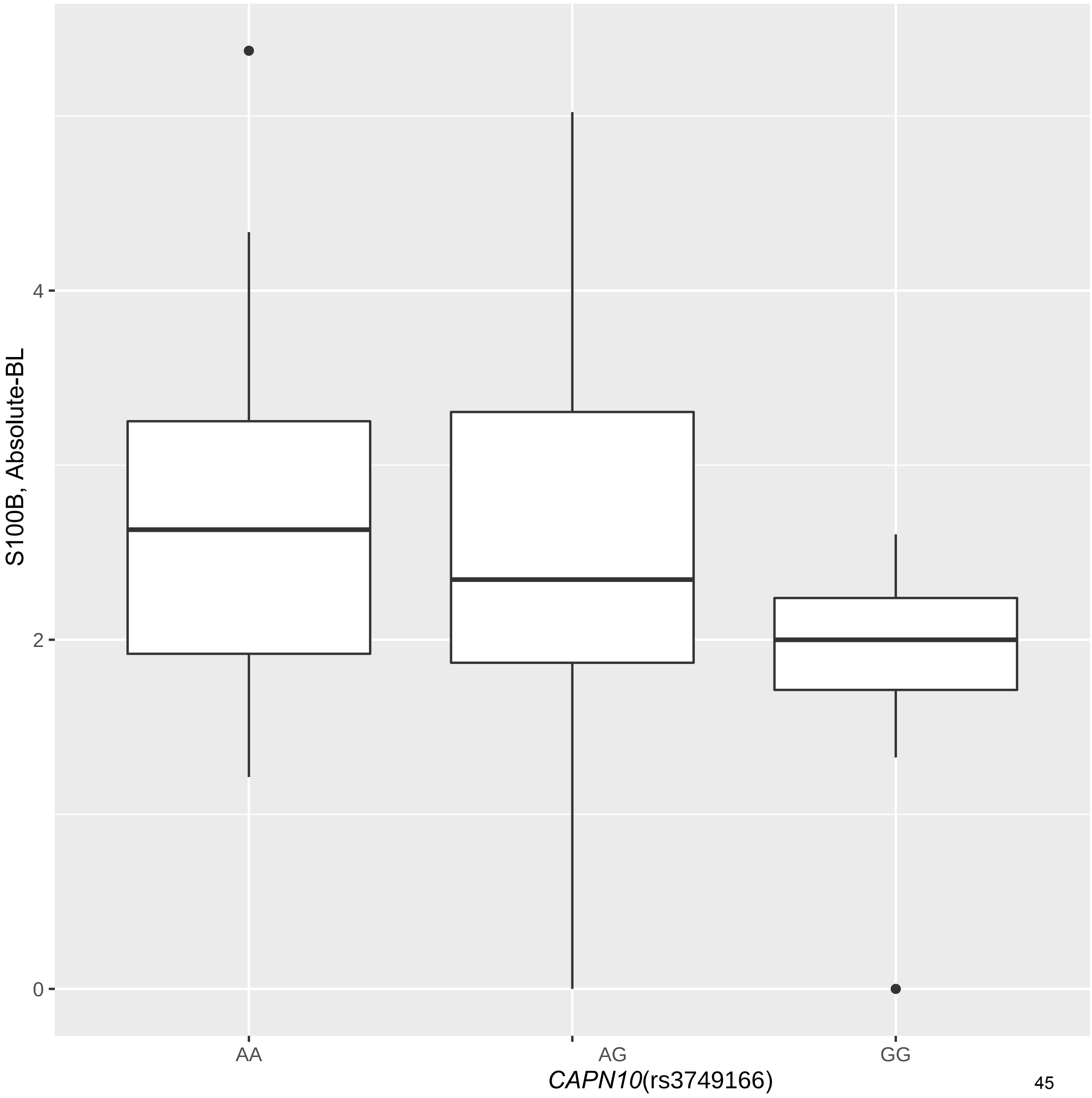
S100B, Absolute-BL
4
2
0
AA
AG
GG
CAPN10(rs3749166)

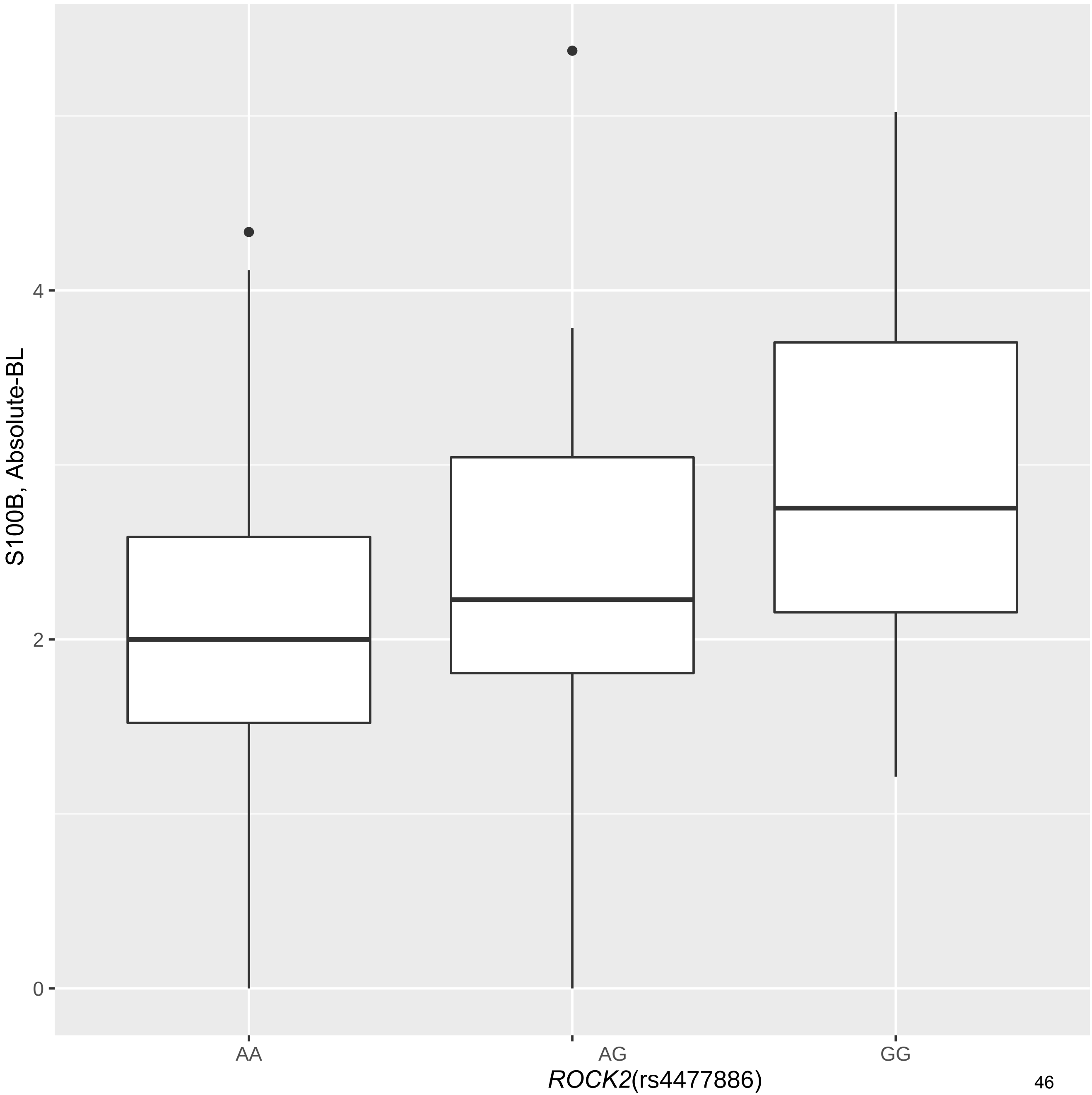
S100B, Absolute-BL
4
2
0
AA
AG
GG
ROCK2(rs4477886)

S100β Absolute Values Associations-aCPB (S100B 2)

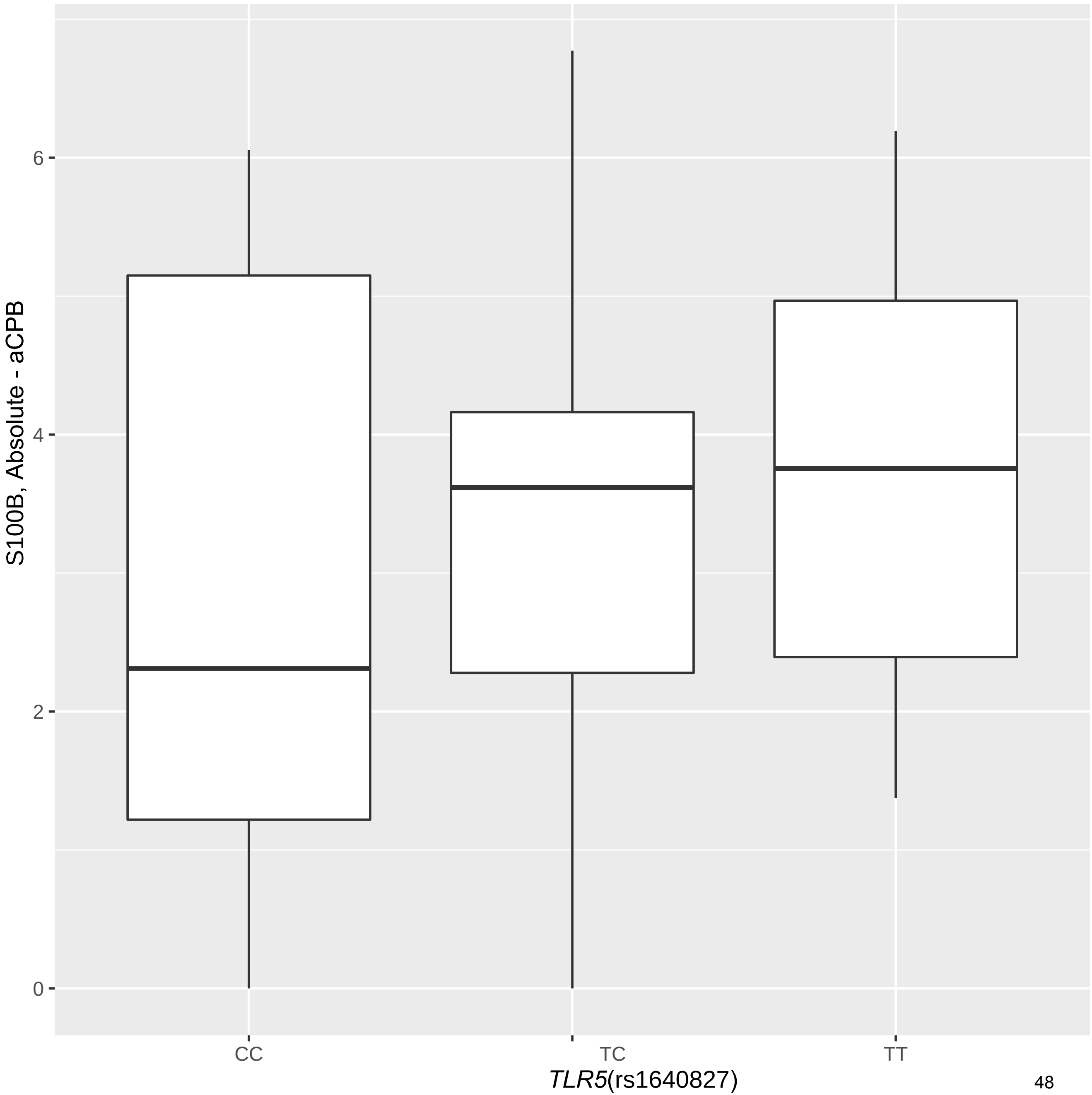
S100B, Absolute - aCPB
0
2
4
6
CC
TC
TT
TLR5(rs1640827)

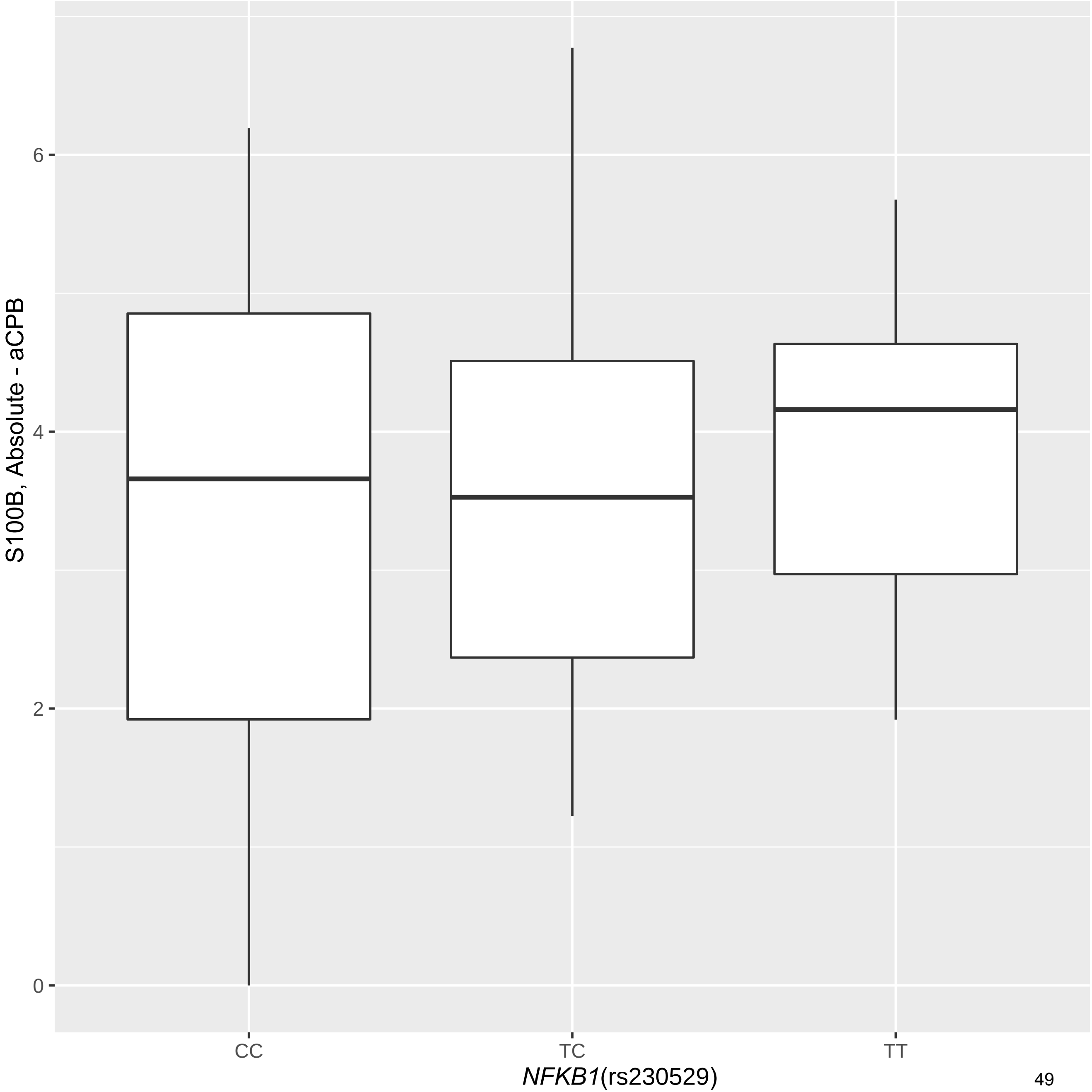
S100B, Absolute - aCPB
6
4
2
0
CC
TC
TT
NFKB1(rs230529)

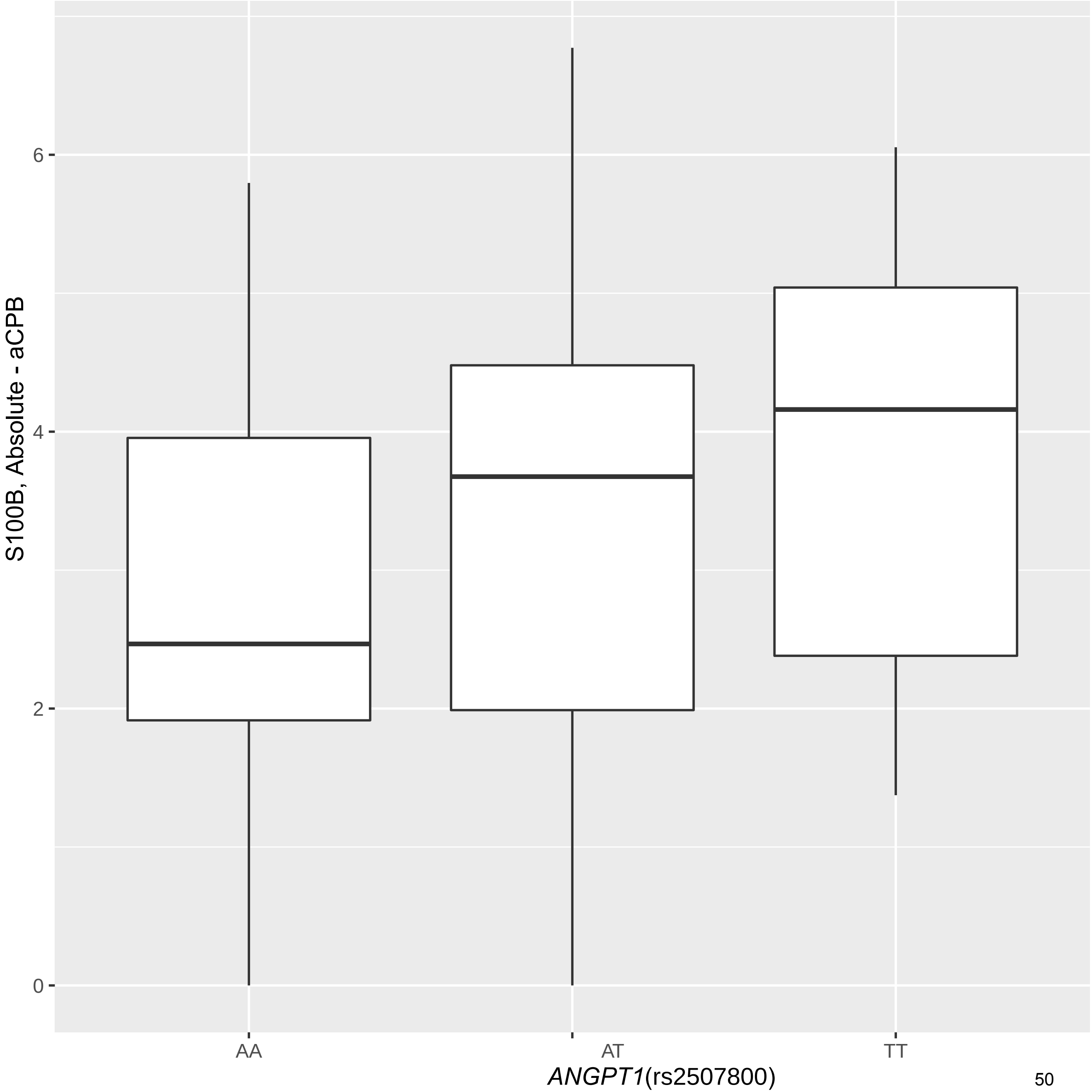
S100B, Absolute - aCPB
0
2
4
6
AA
AT
TT
ANGPT1(rs2507800)

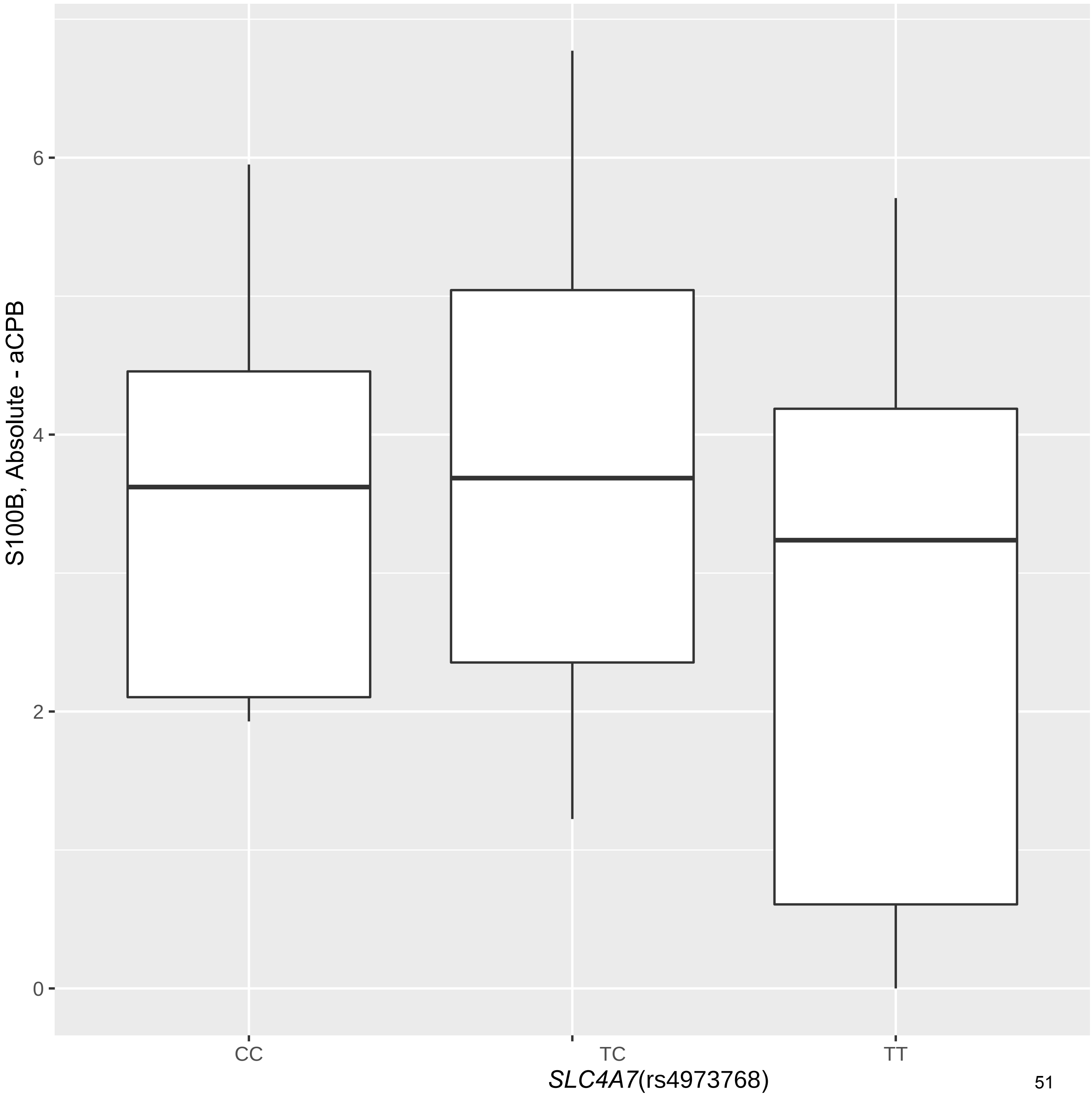
S100B, Absolute - aCPB
0
2
4
6
CC
TC
TT
SLC4A7(rs4973768)

S100β Absolute Values Associations-pCPB (S100B 5)

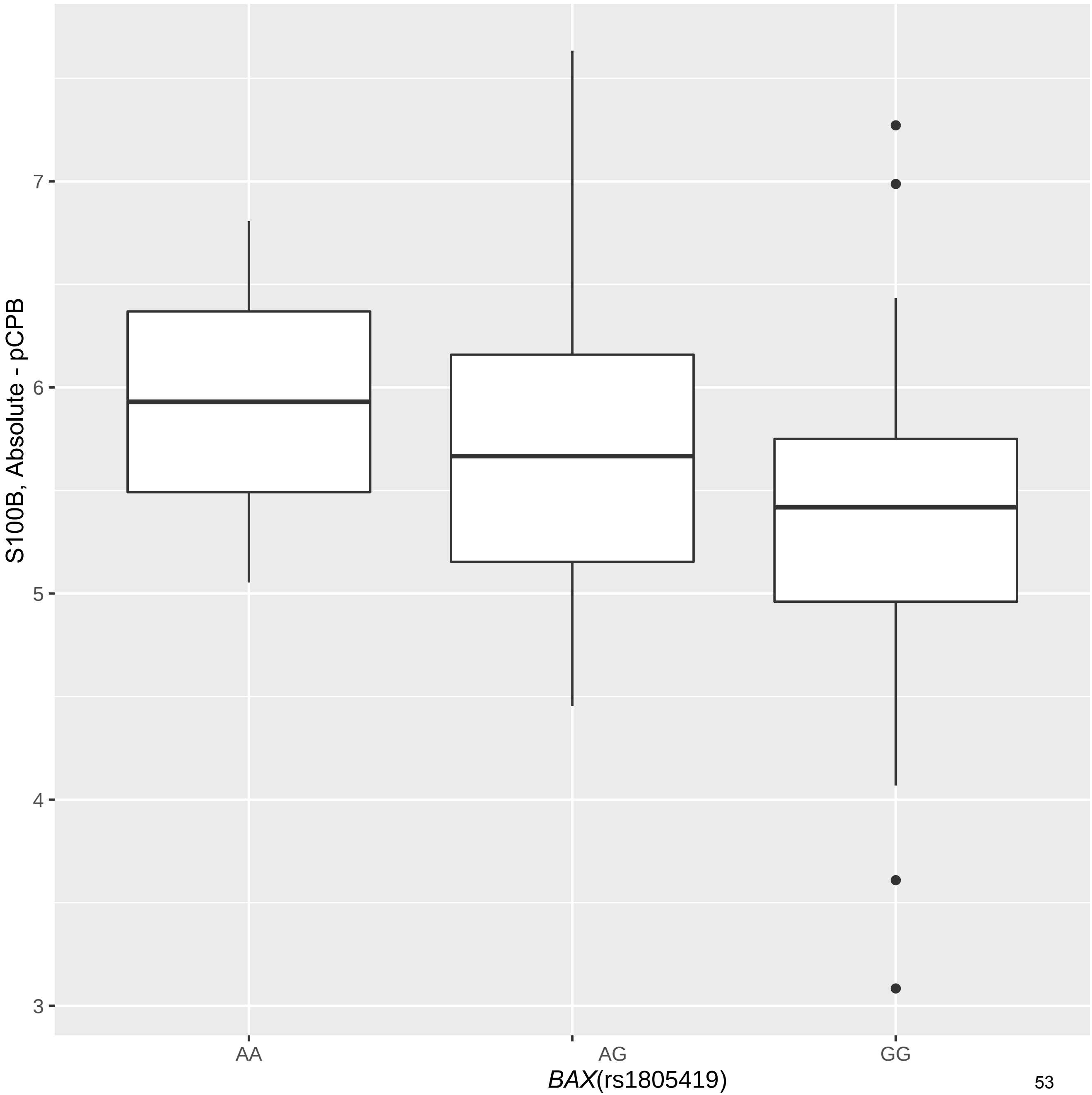
S100B, Absolute - pCPB
7
6
5
4
3
AA
AG
GG
BAX(rs1805419)

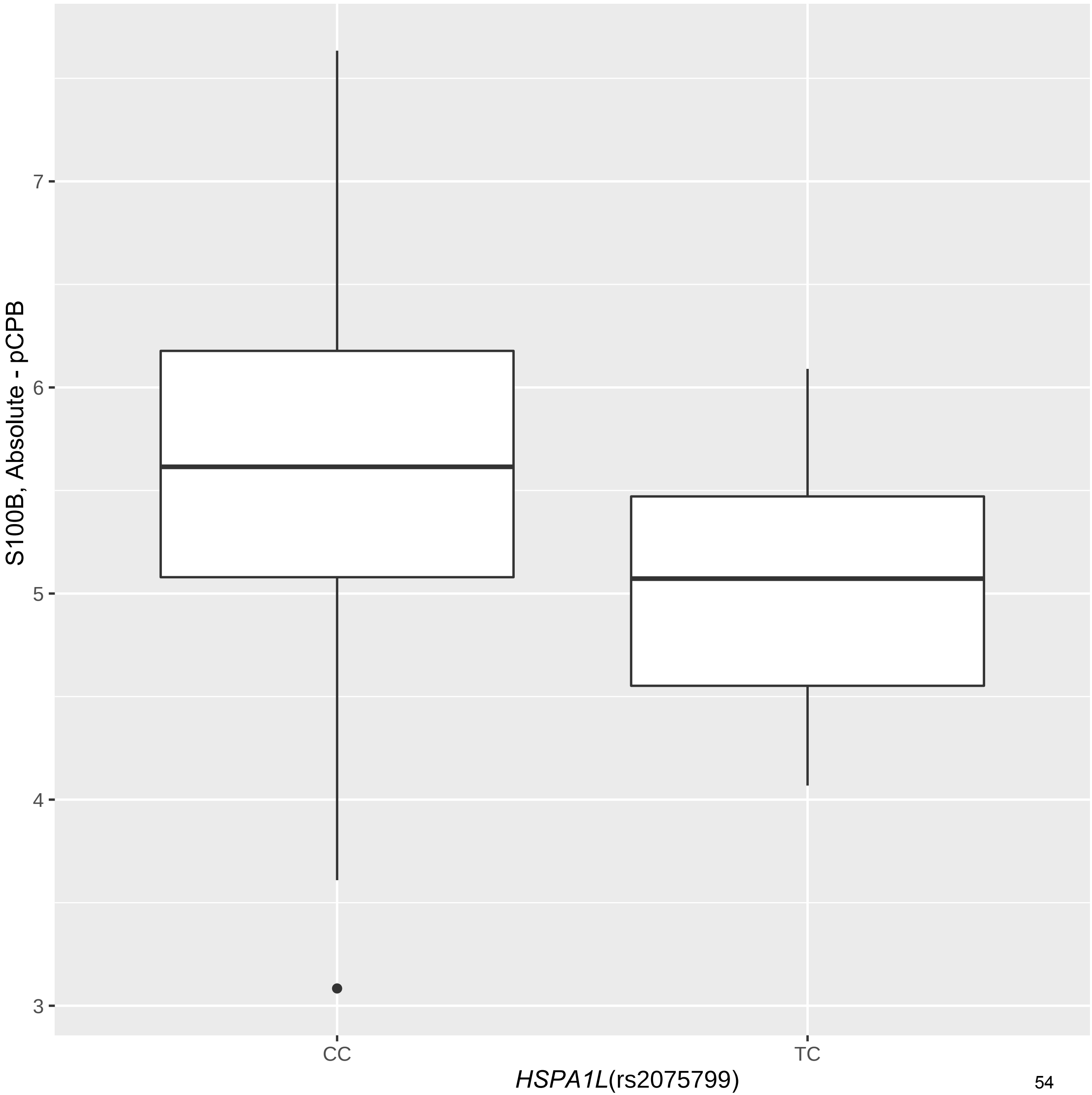

S100B, Absolute - pCPB
7
6
5
4
3
CC
TC
HSPA1L(rs2075799)

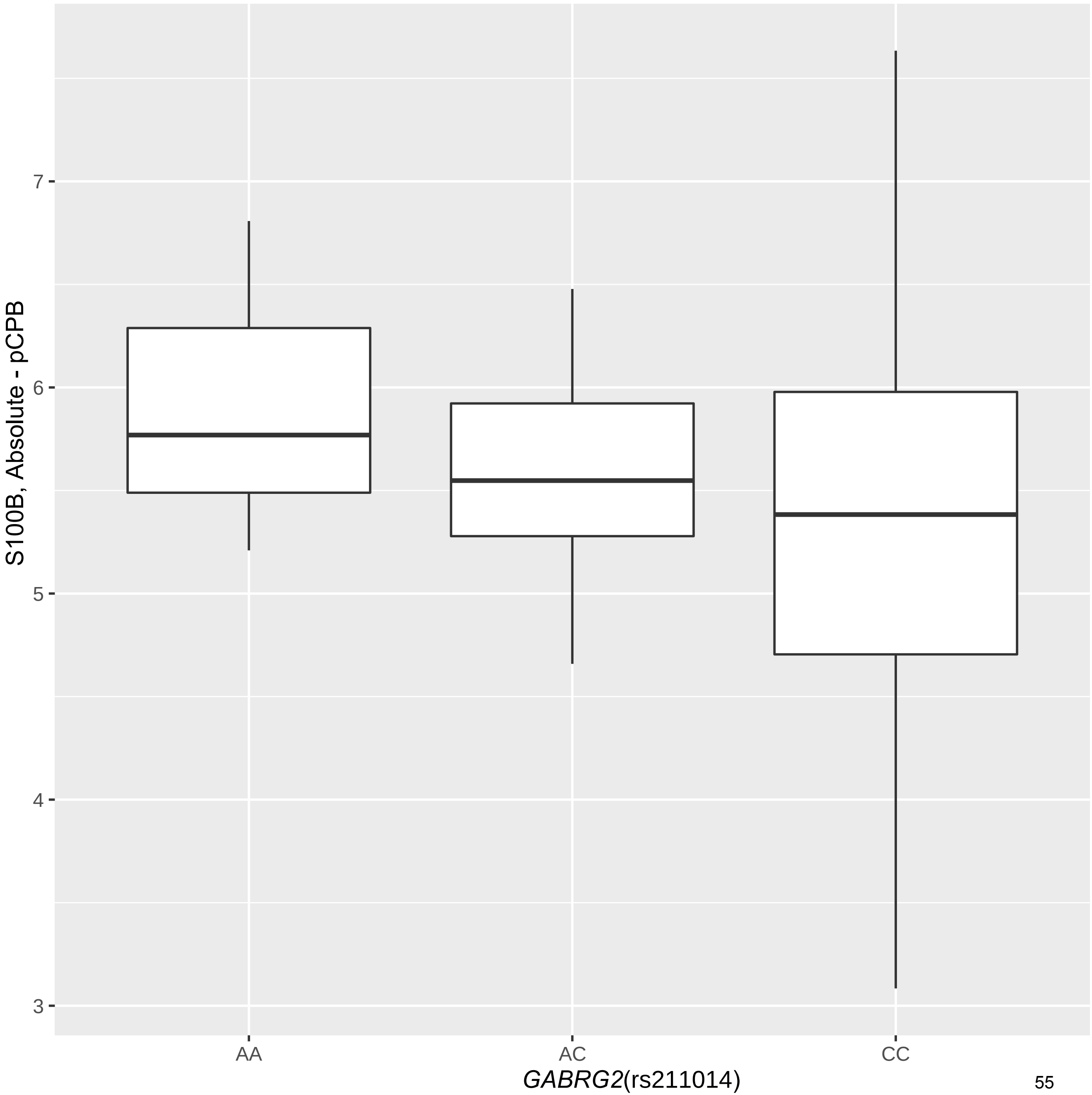

S100B, Absolute - pCPB
7
6
5
4
3
AA
AC
CC
GABRG2(rs211014)

S100β Absolute Values Associations-24H (S100B 6)

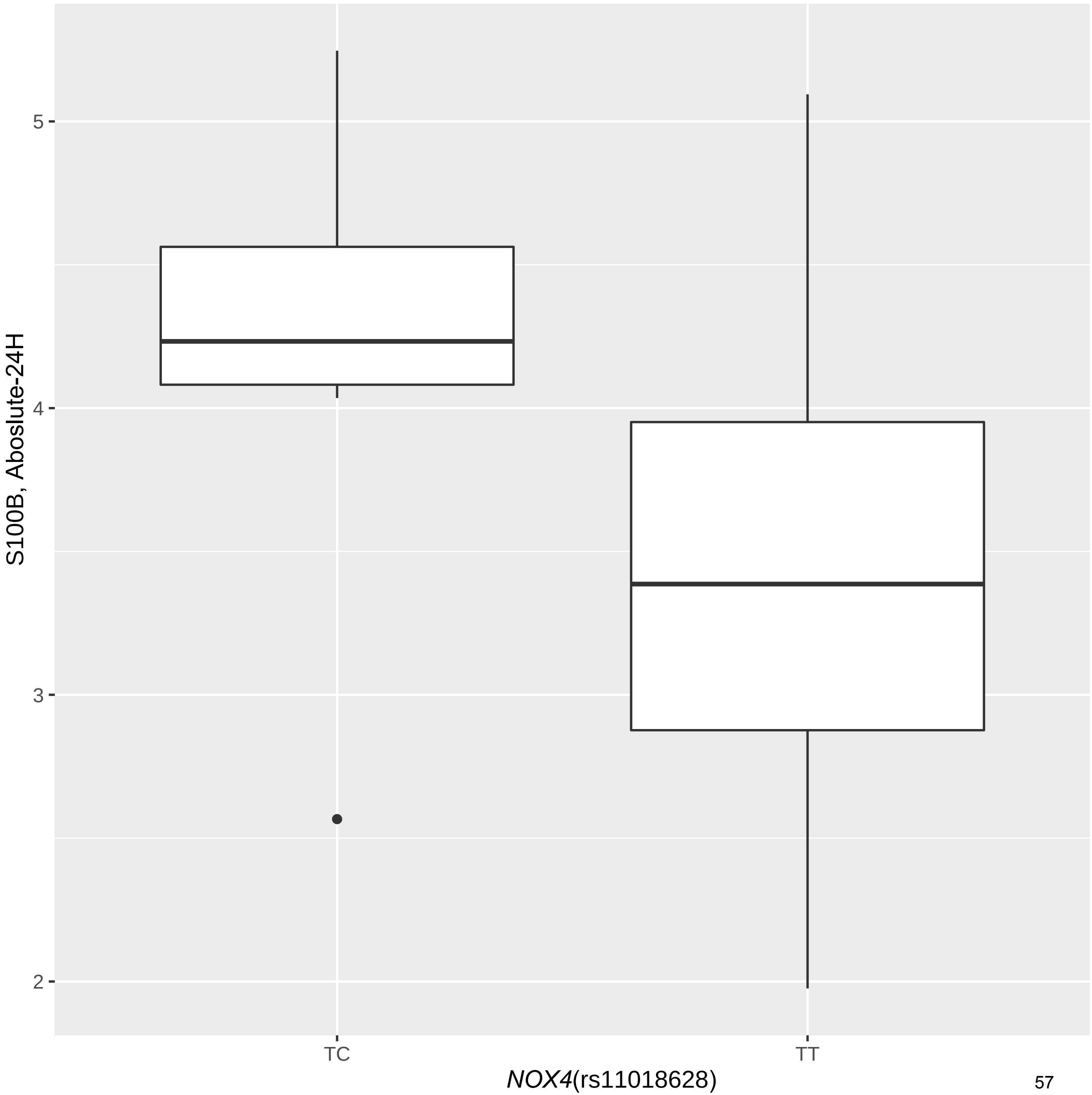

S100B, Aboslute-24H
5
4
3
2
TC
TT
NOX4(rs11018628)

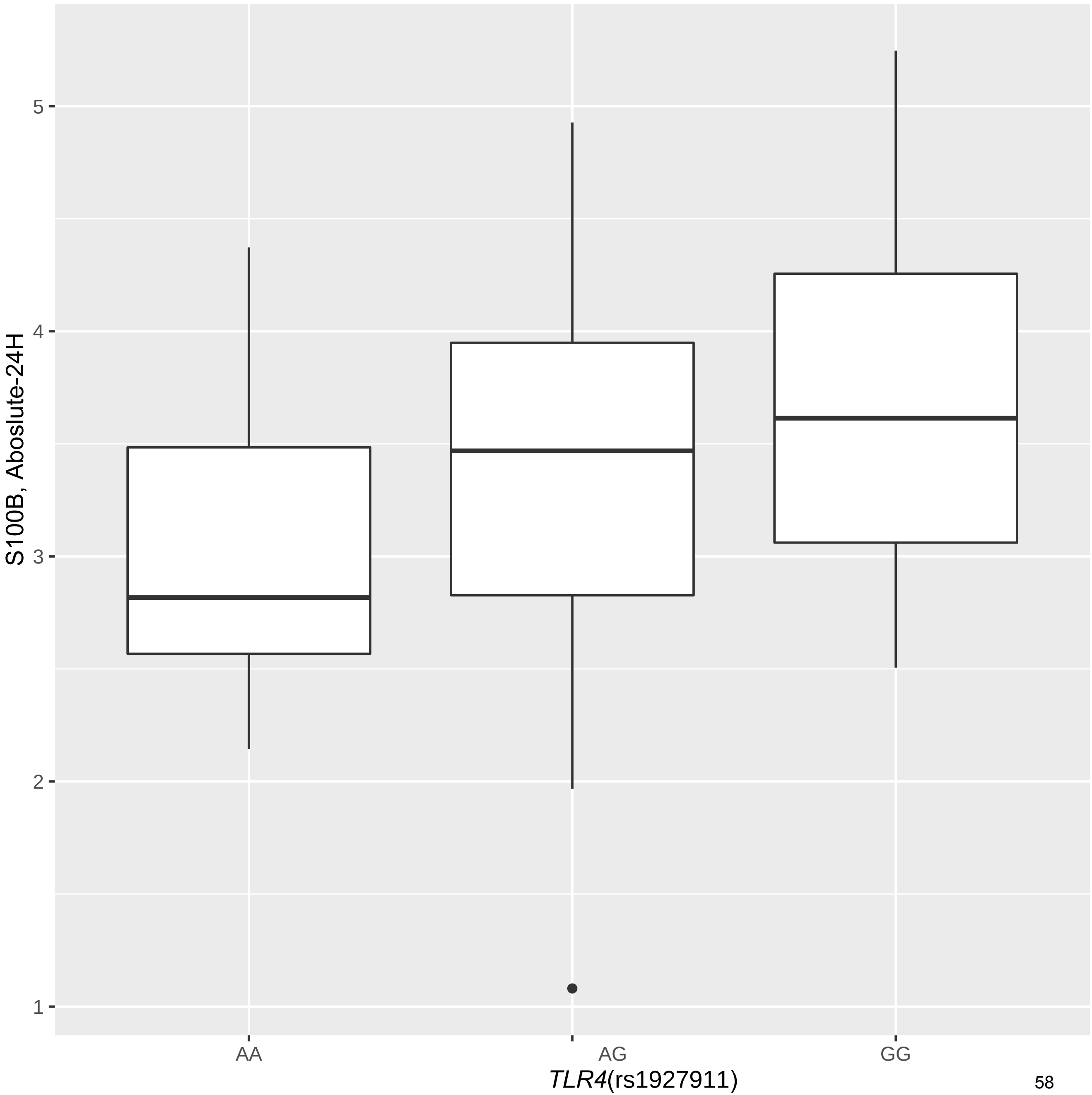
S100B, Aboslute-24H
5
4
3
2
1
AA
AG
GG
TLR4(rs1927911)

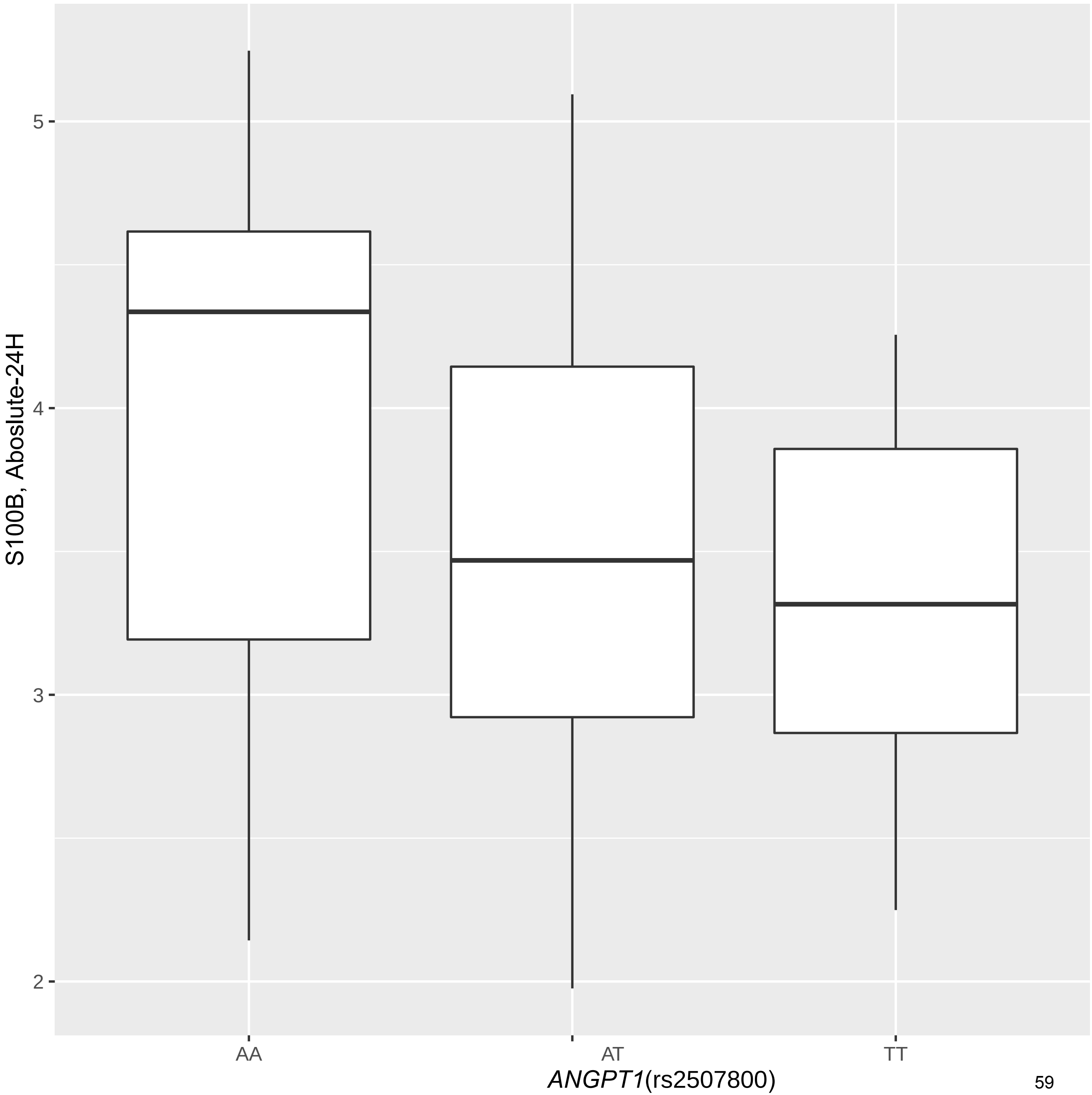

S100B, Aboslute-24H
5
4
3
2
AA
AT
TT
ANGPT1(rs2507800)

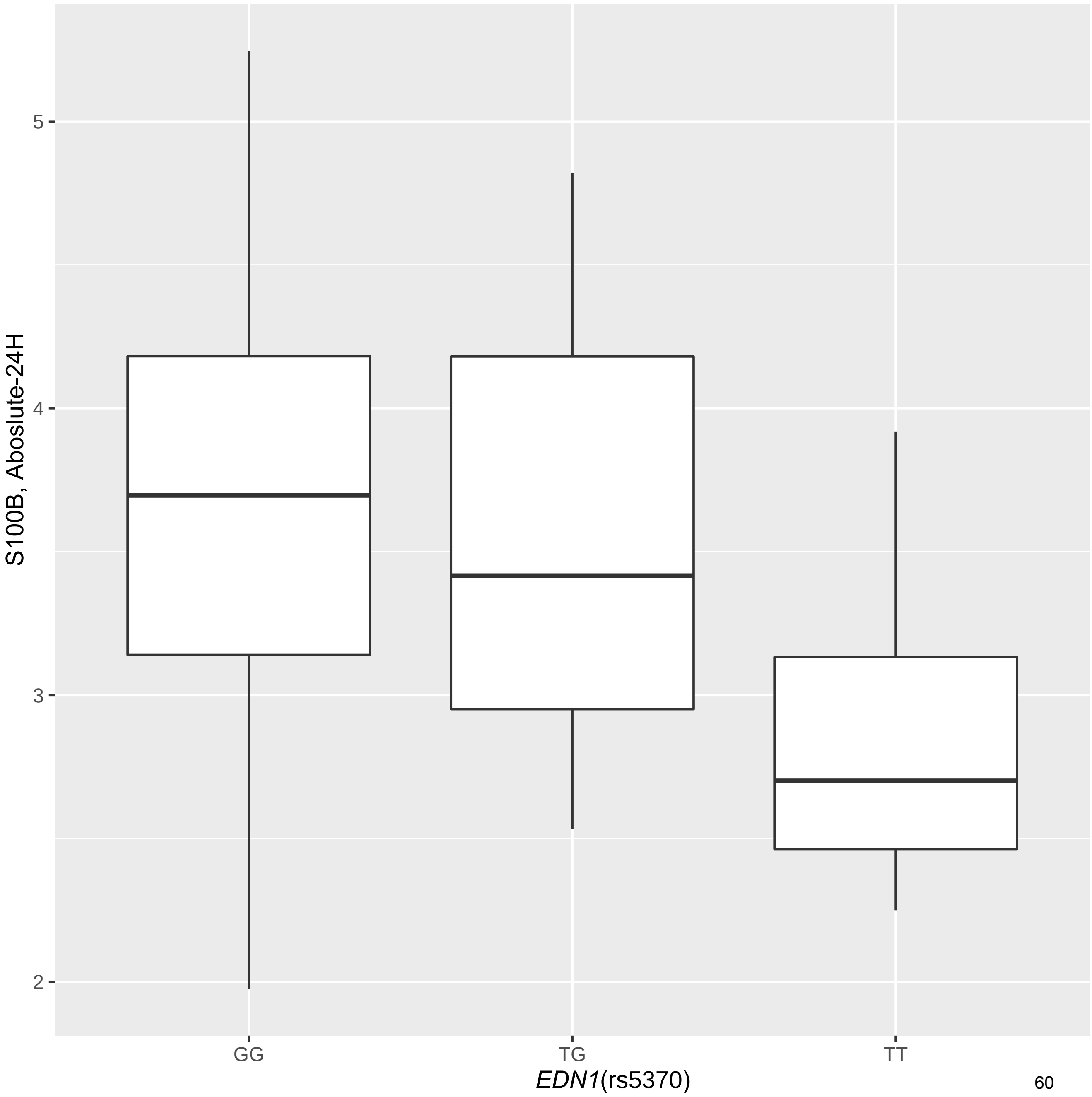
S100B, Aboslute-24H
5
4
3
2
GG
TG
TT
EDN1(rs5370)

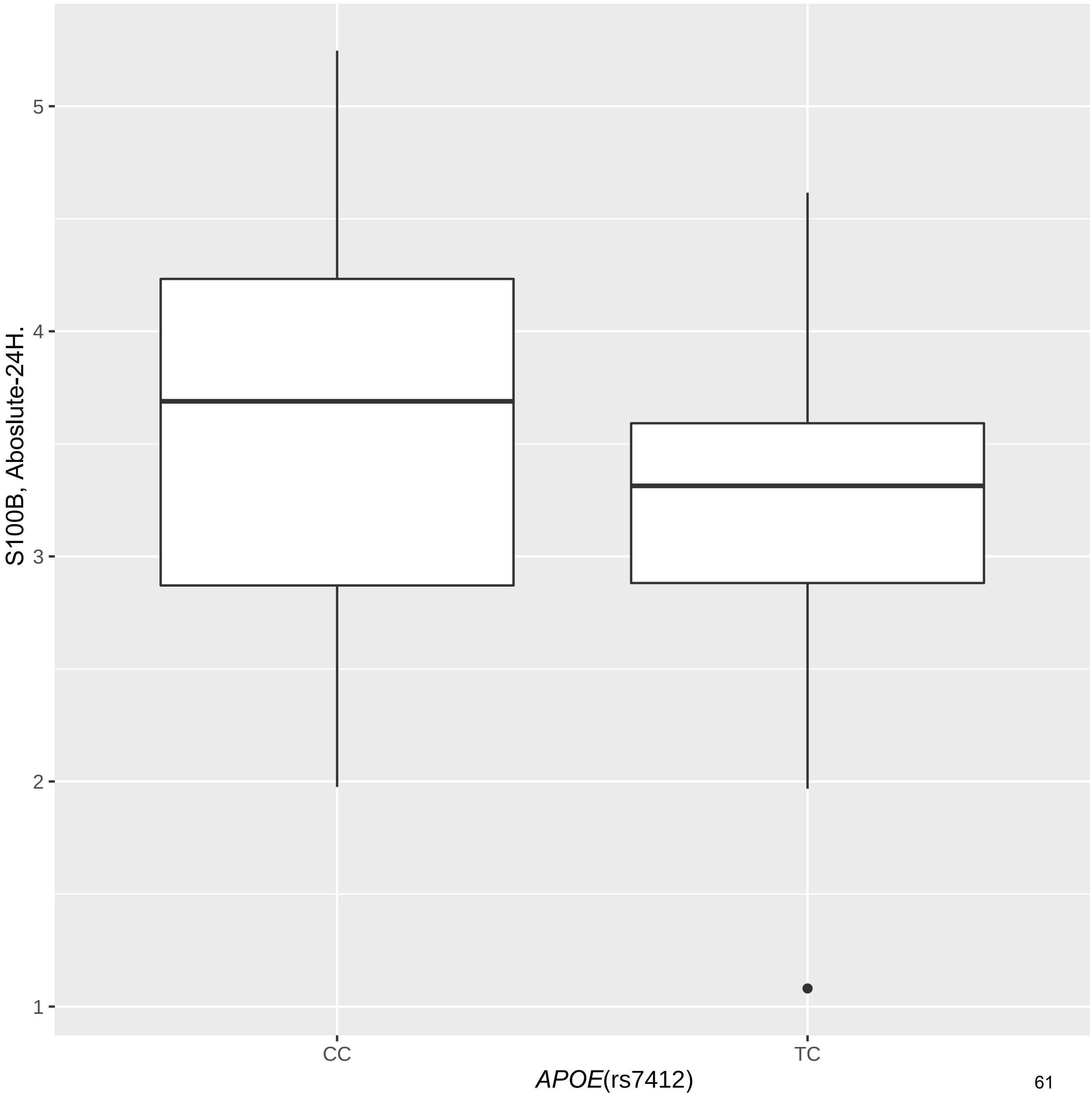
S100B, Aboslute-24H.
5
4
3
2
1
CC
TC
APOE(rs7412)

Change in NFH Values-BL vs aCPB (chNFH12)

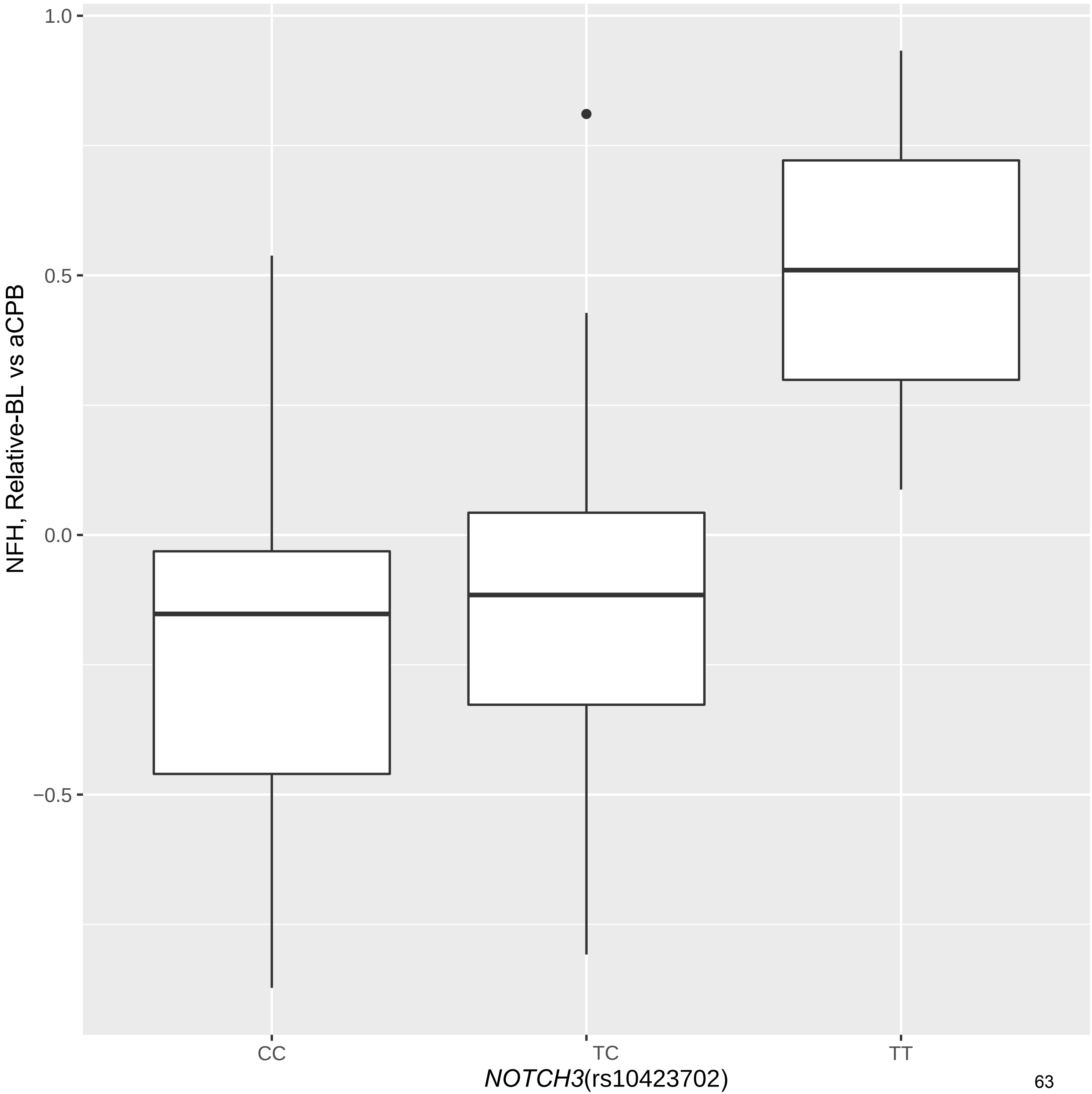
1.0
0.5
0.0
−0.5
NFH, Relative-BL vs aCPB
CC
TC
TT
NOTCH3(rs10423702)

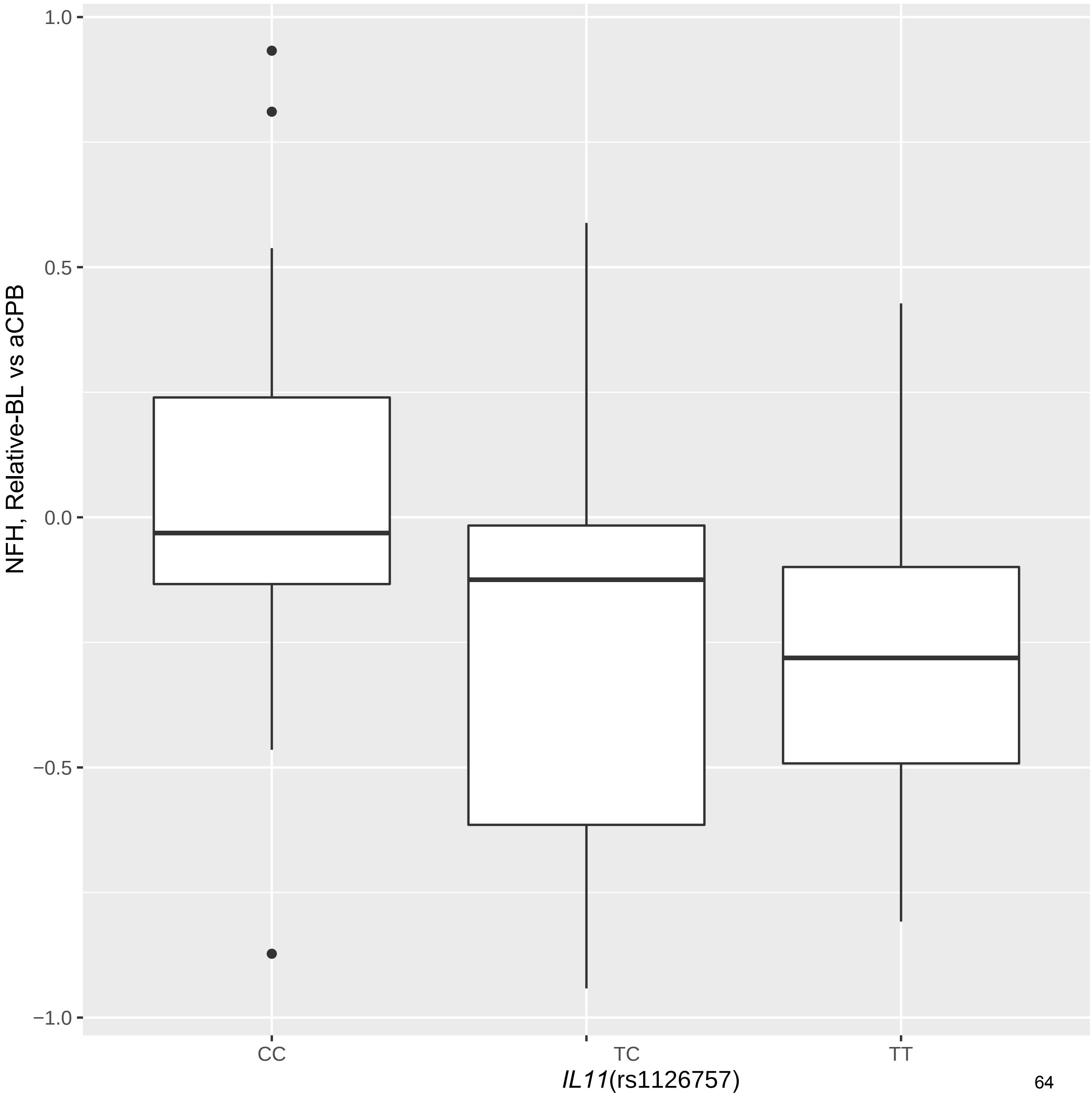
1.0
0.5
0.0
−0.5
−1.0
NFH, Relative-BL vs aCPB
CC
TC
TT
IL11(rs1126757)

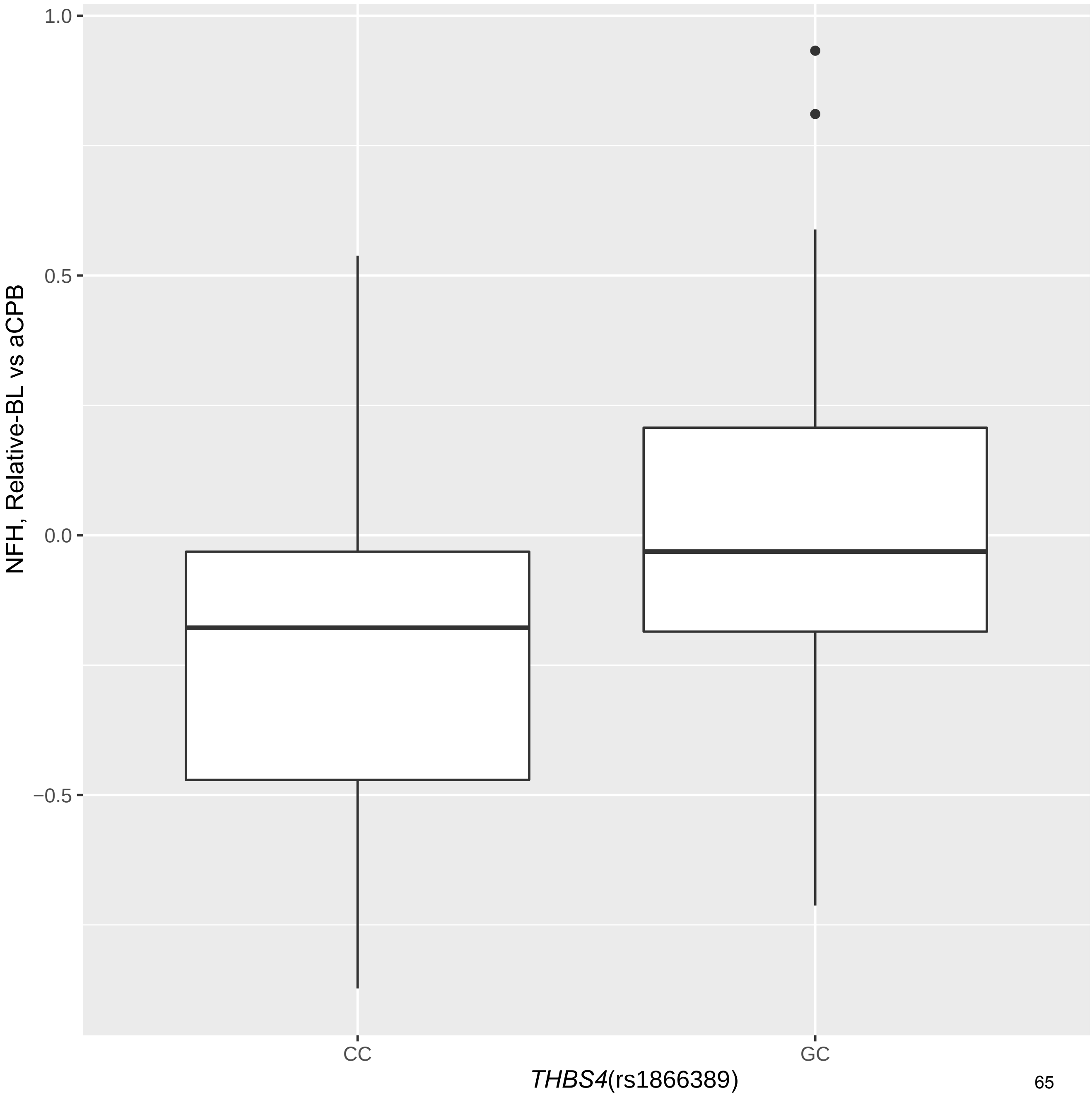
1.0
0.5
0.0
−0.5
NFH, Relative-BL vs aCPB
CC
GC
THBS4(rs1866389)

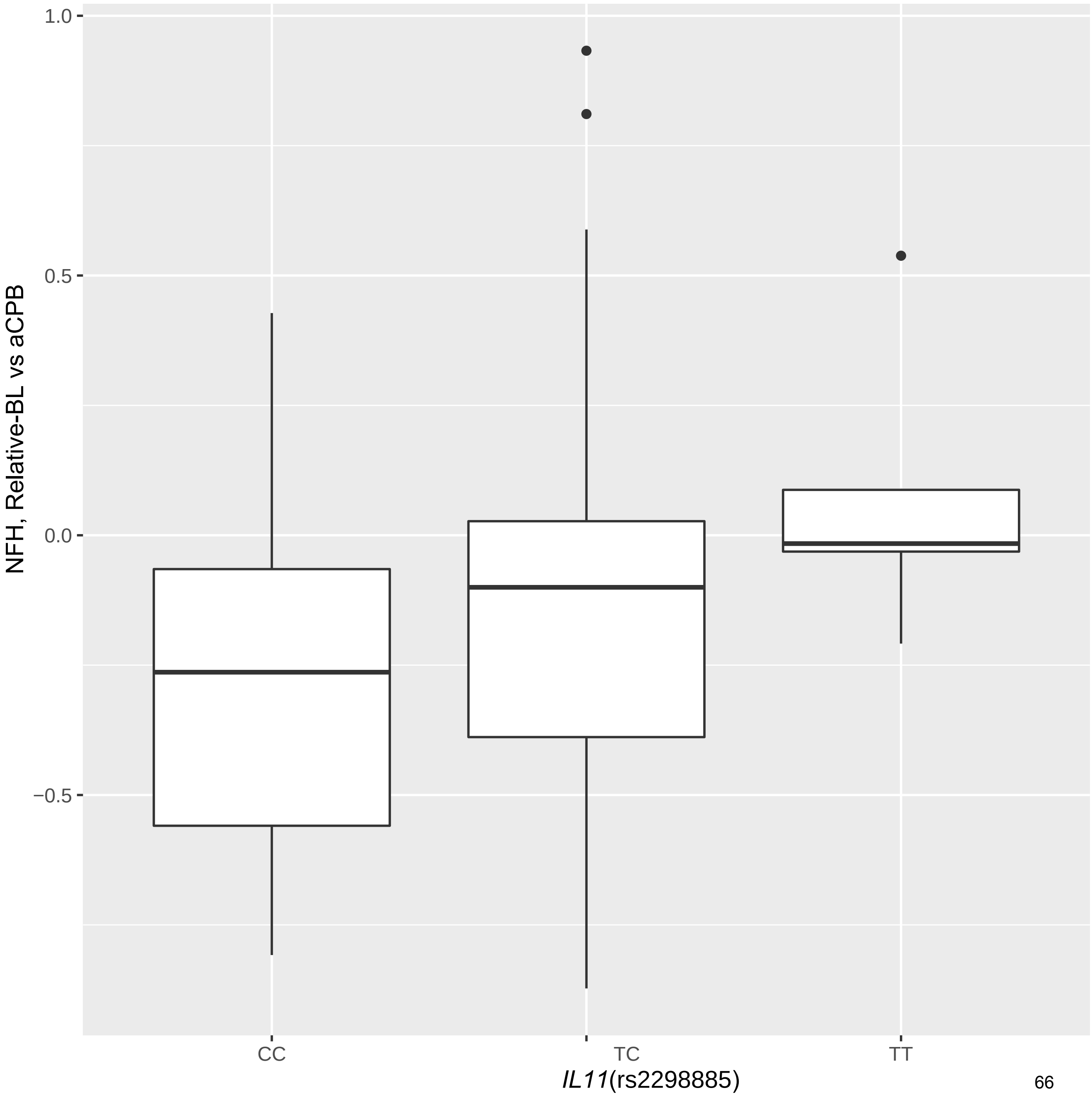
1.0
0.5
0.0
−0.5
NFH, Relative-BL vs aCPB
CC
TC
TT
IL11(rs2298885)

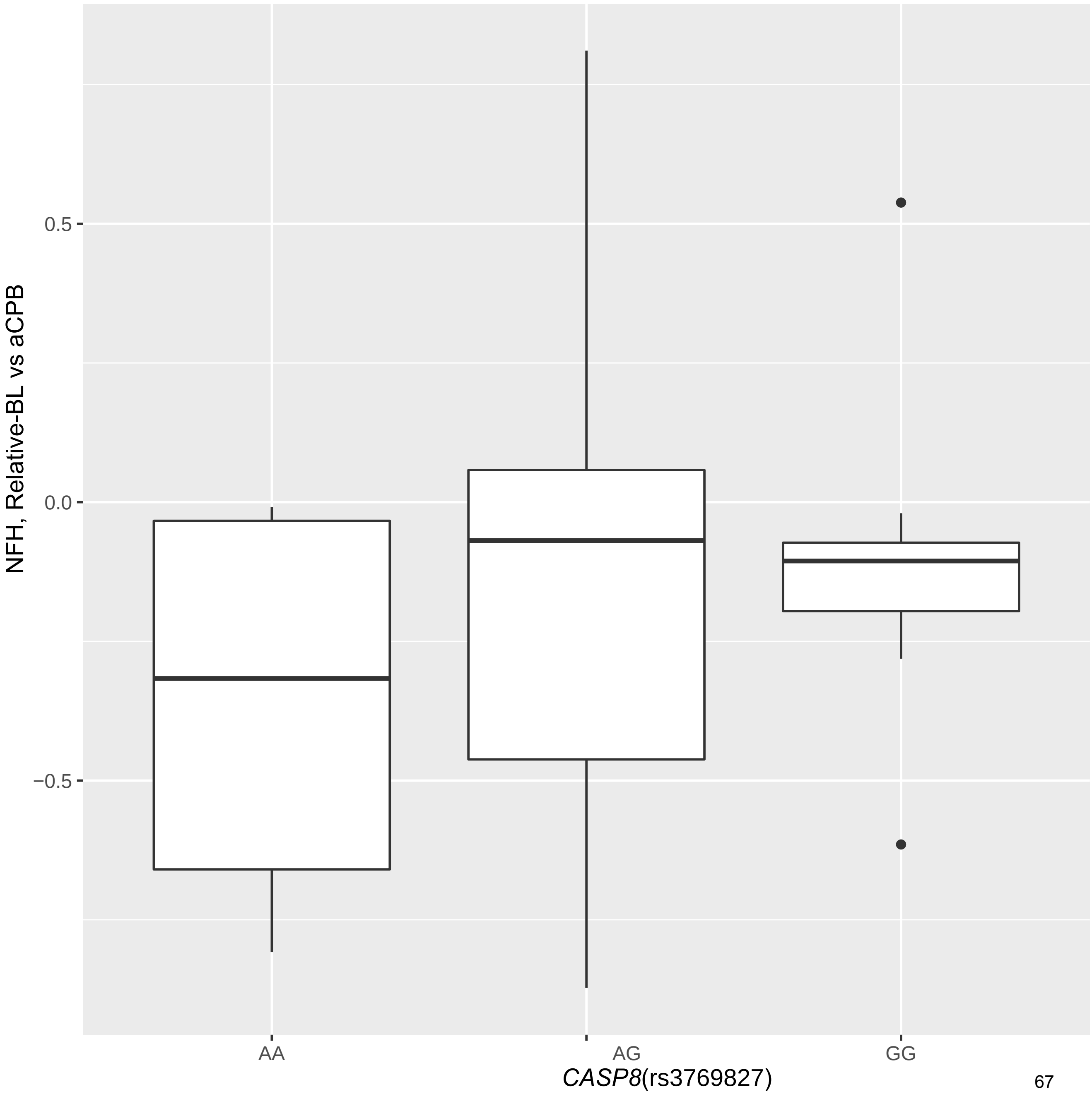
NFH, Relative-BL vs aCPB
0.5
0.0
−0.5
AA
AG
GG
CASP8(rs3769827)

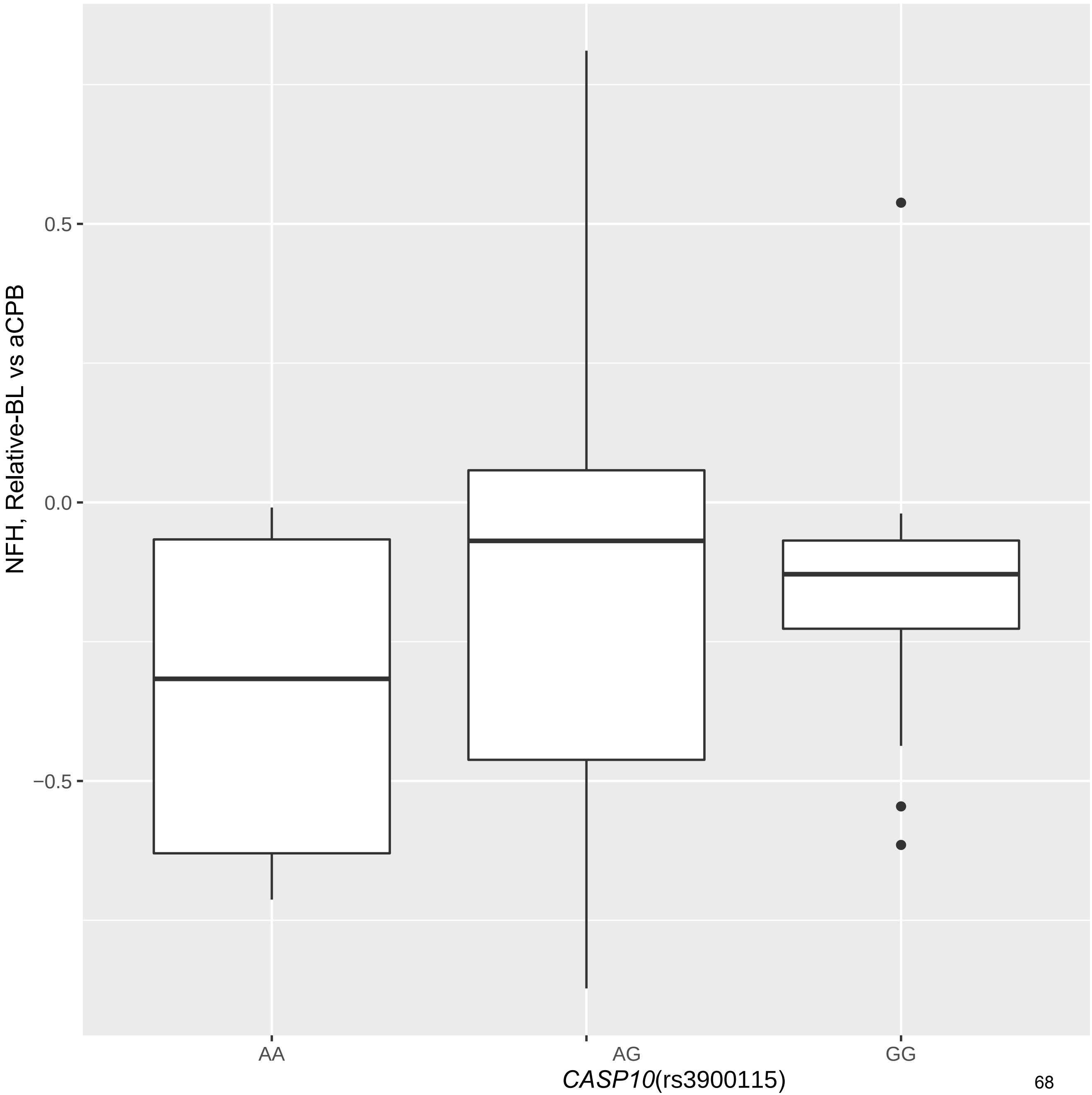
NFH, Relative-BL vs aCPB
0.5
0.0
−0.5
AA
AG
GG
CASP10(rs3900115)

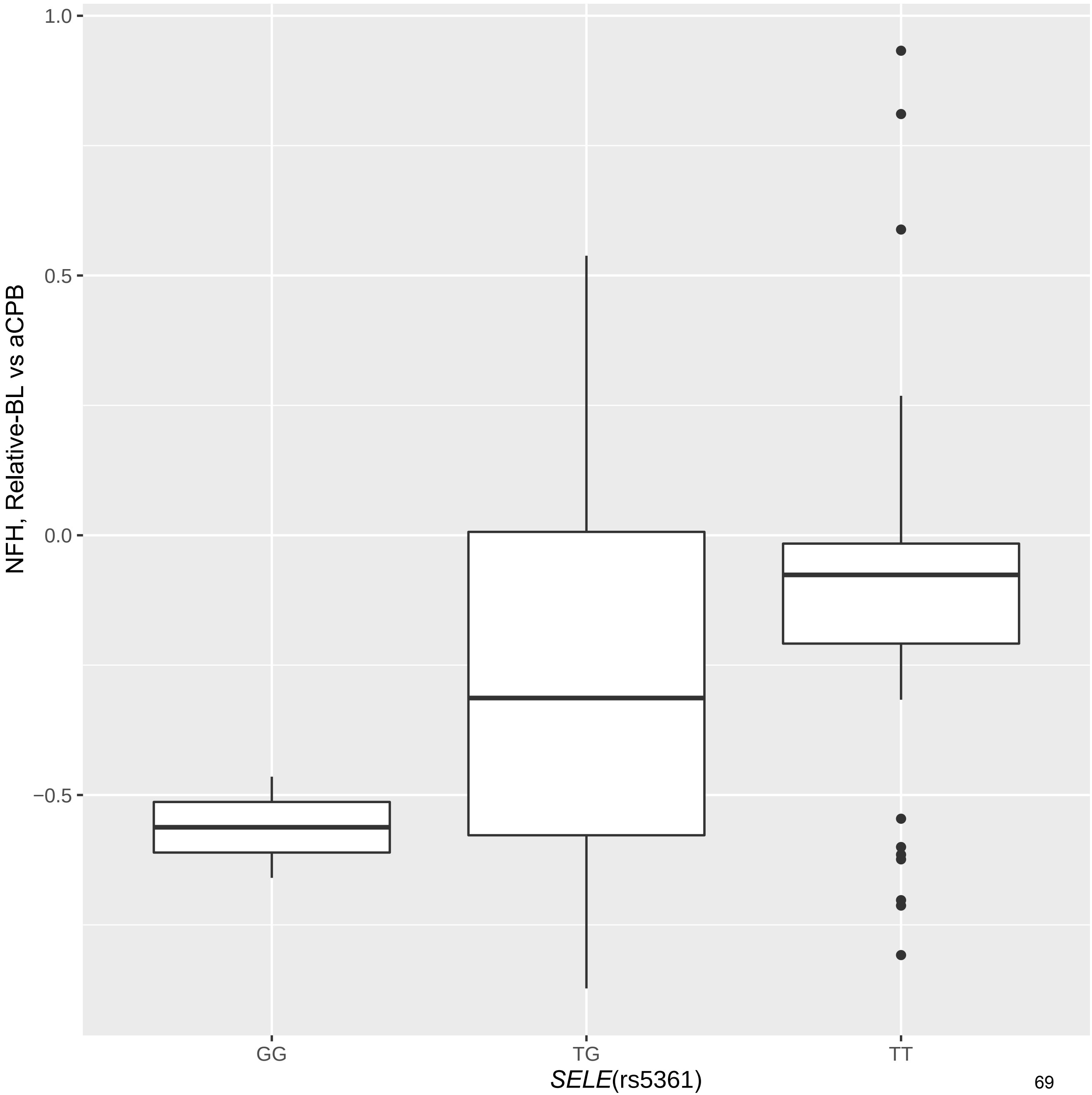

1.0
0.5
0.0
−0.5
NFH, Relative-BL vs aCPB
GG
TG
TT
SELE(rs5361)

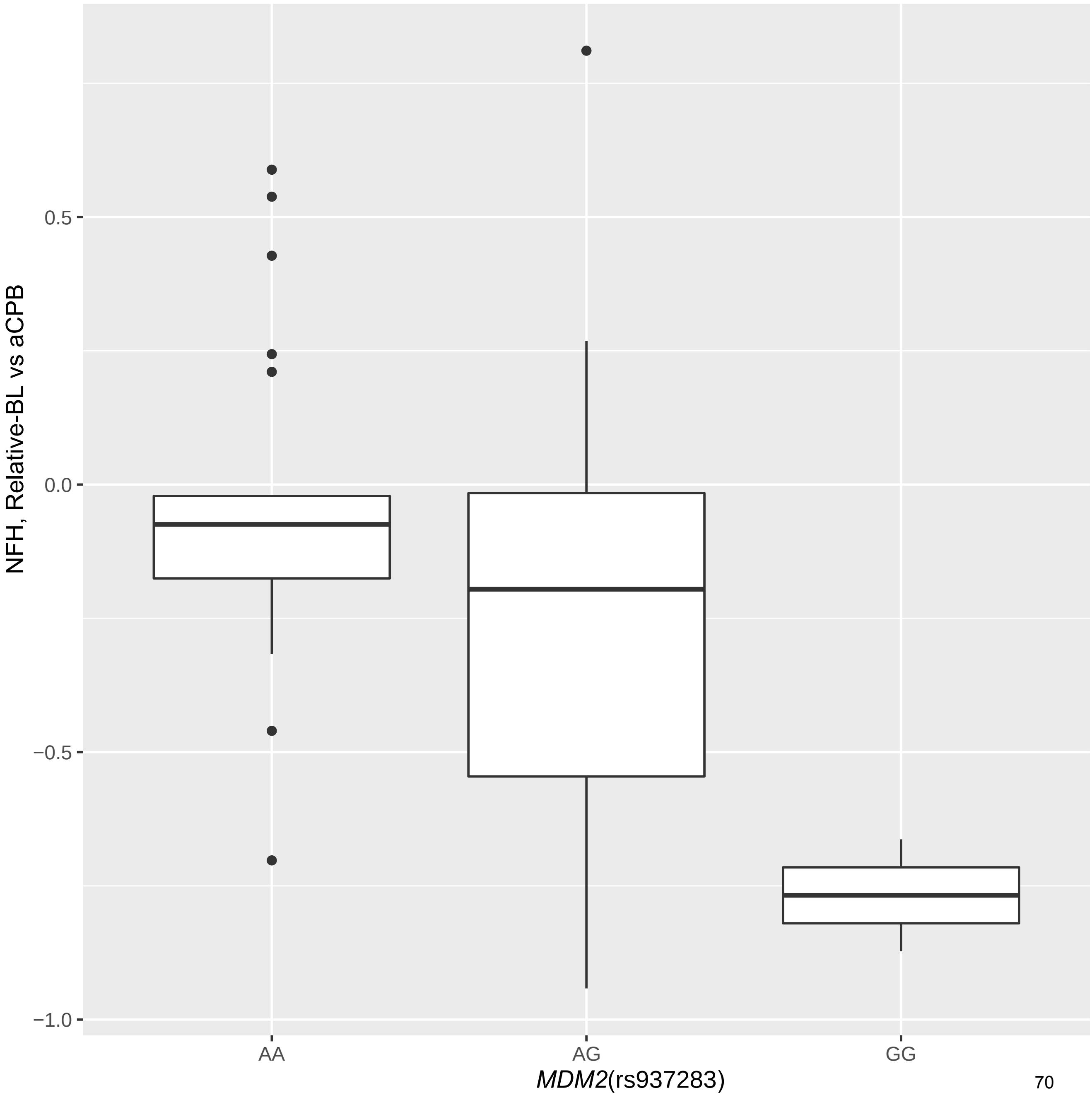

NFH, Relative-BL vs aCPB
0.5
0.0
−0.5
−1.0
AA
AG
GG
MDM2(rs937283)

Change in NFH Values-BL vs pCPB (chNFH 15)

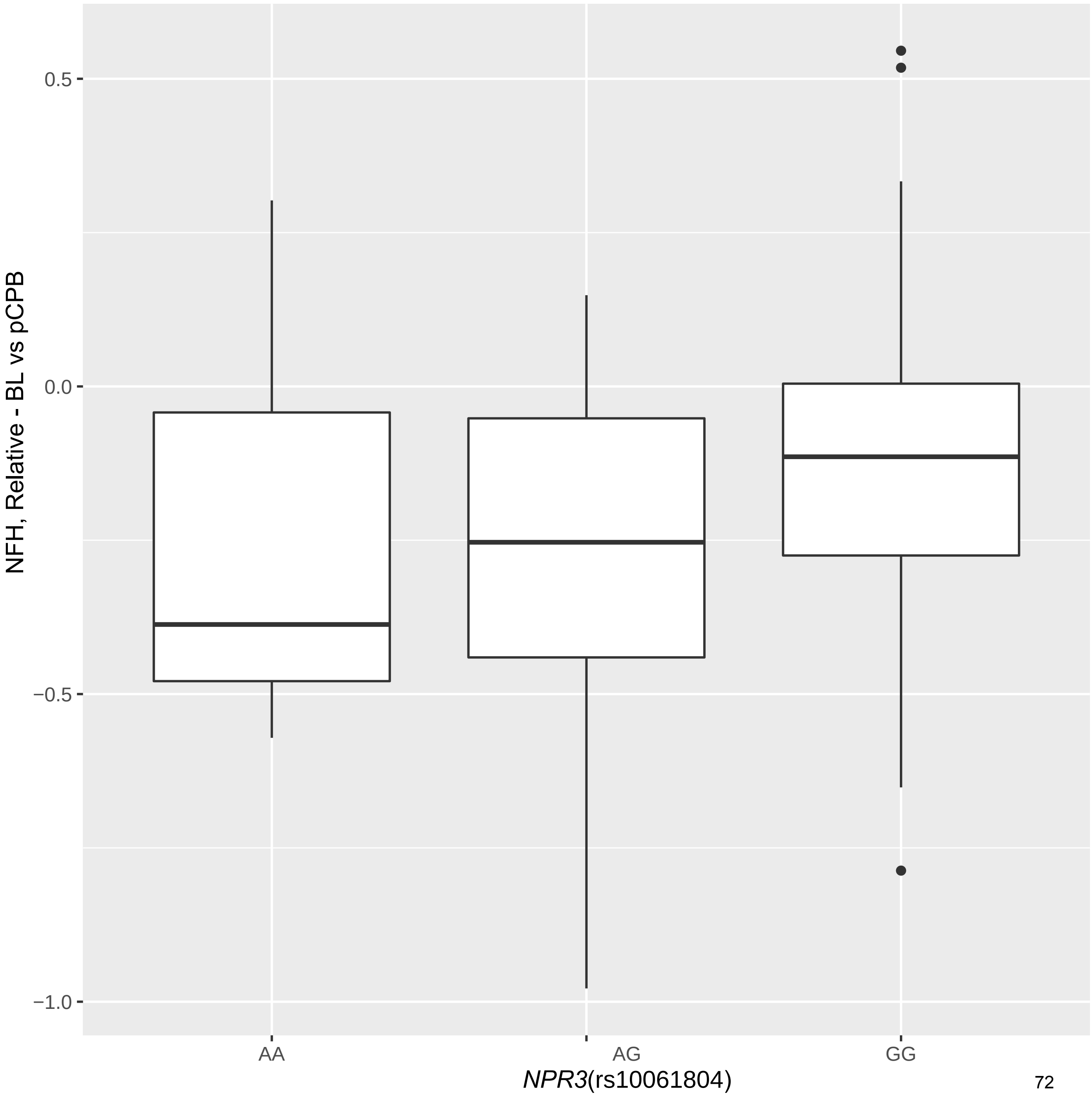

NFH, Relative - BL vs pCPB
0.5
0.0
−0.5
−1.0
AA
AG
GG
NPR3(rs10061804)

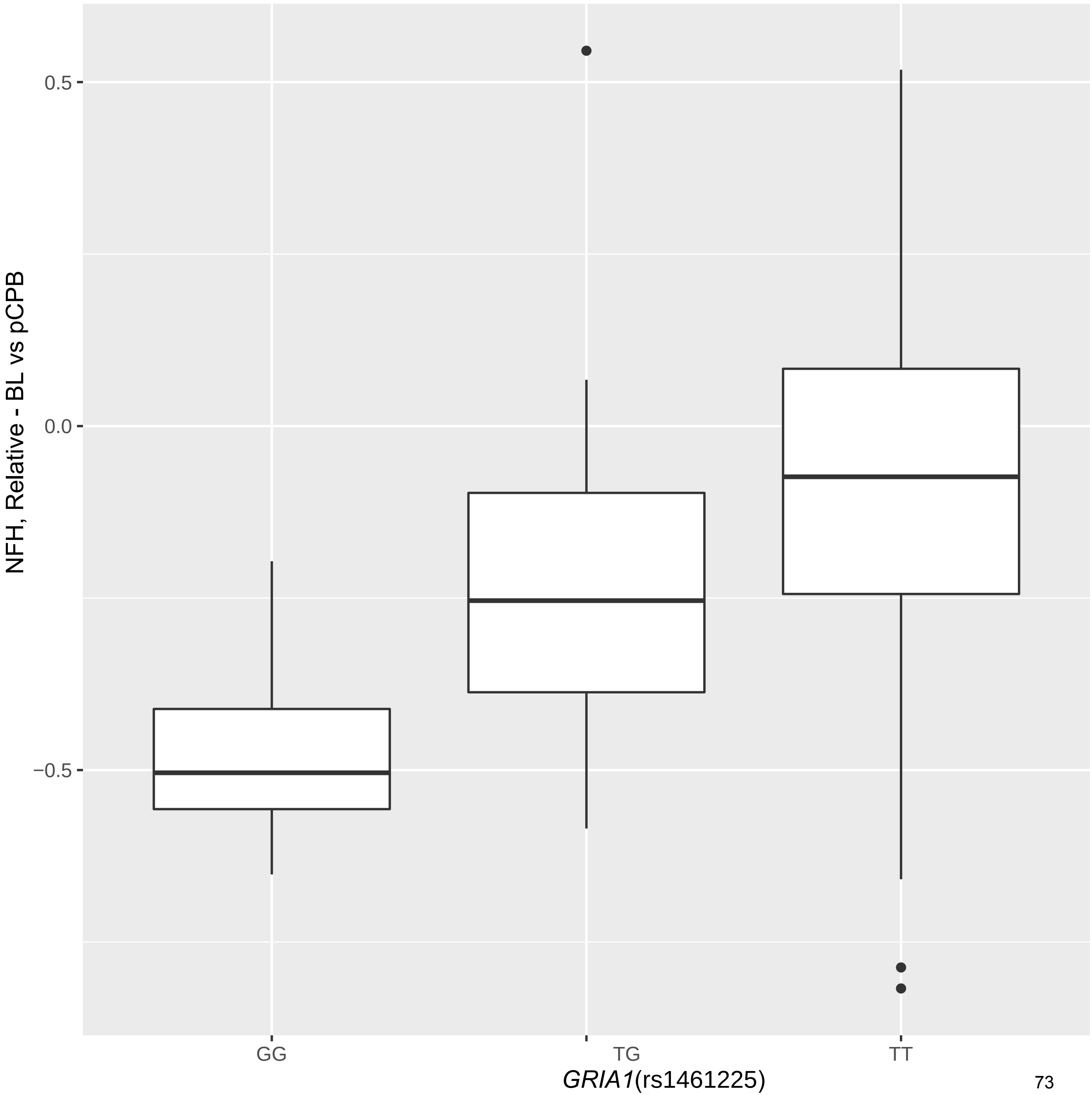

0.5
0.0
−0.5
NFH, Relative - BL vs pCPB
GG
TG
TT
GRIA1(rs1461225)

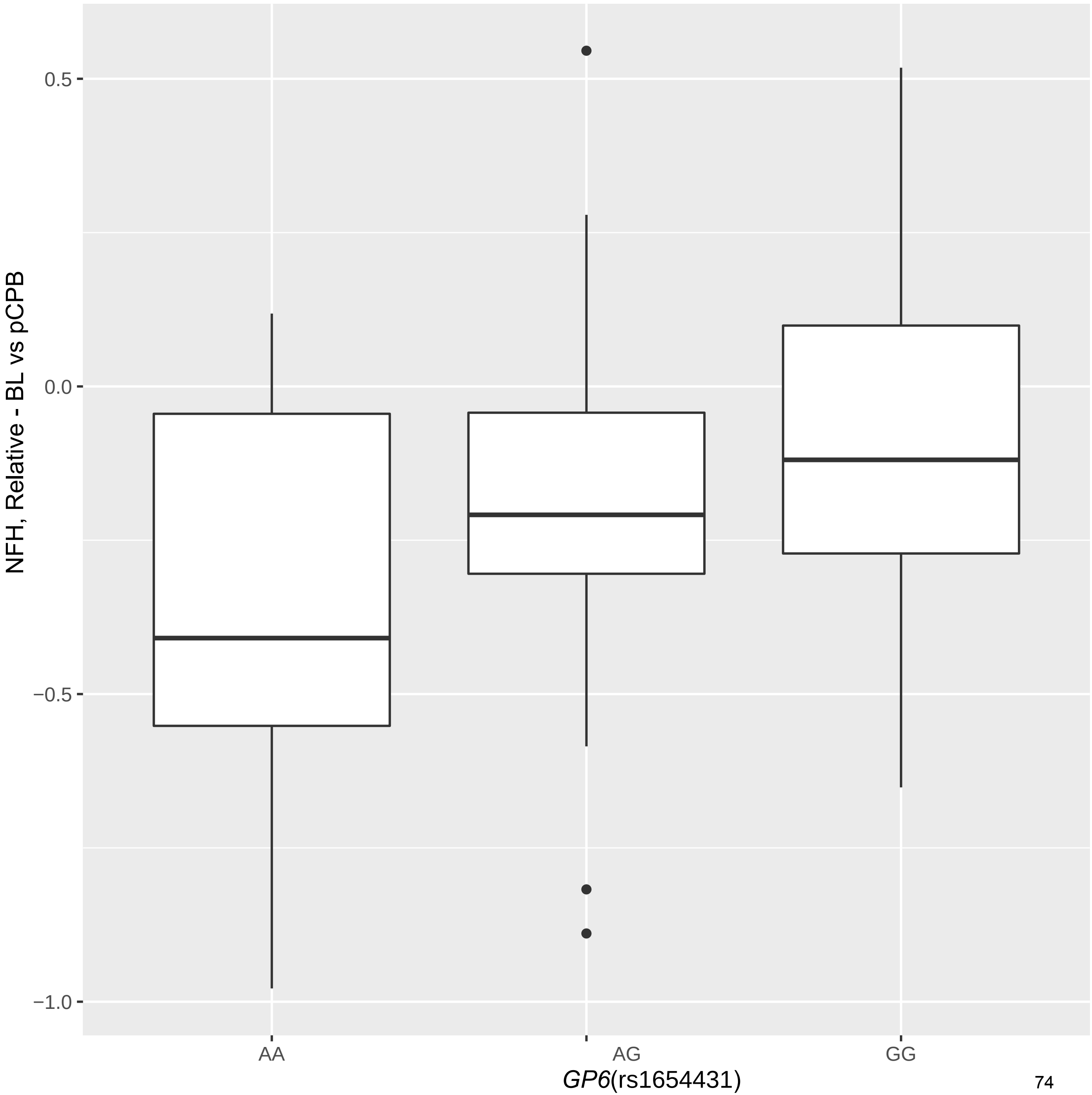
NFH, Relative - BL vs pCPB
0.5
0.0
−0.5
−1.0
AA
AG
GG
GP6(rs1654431)

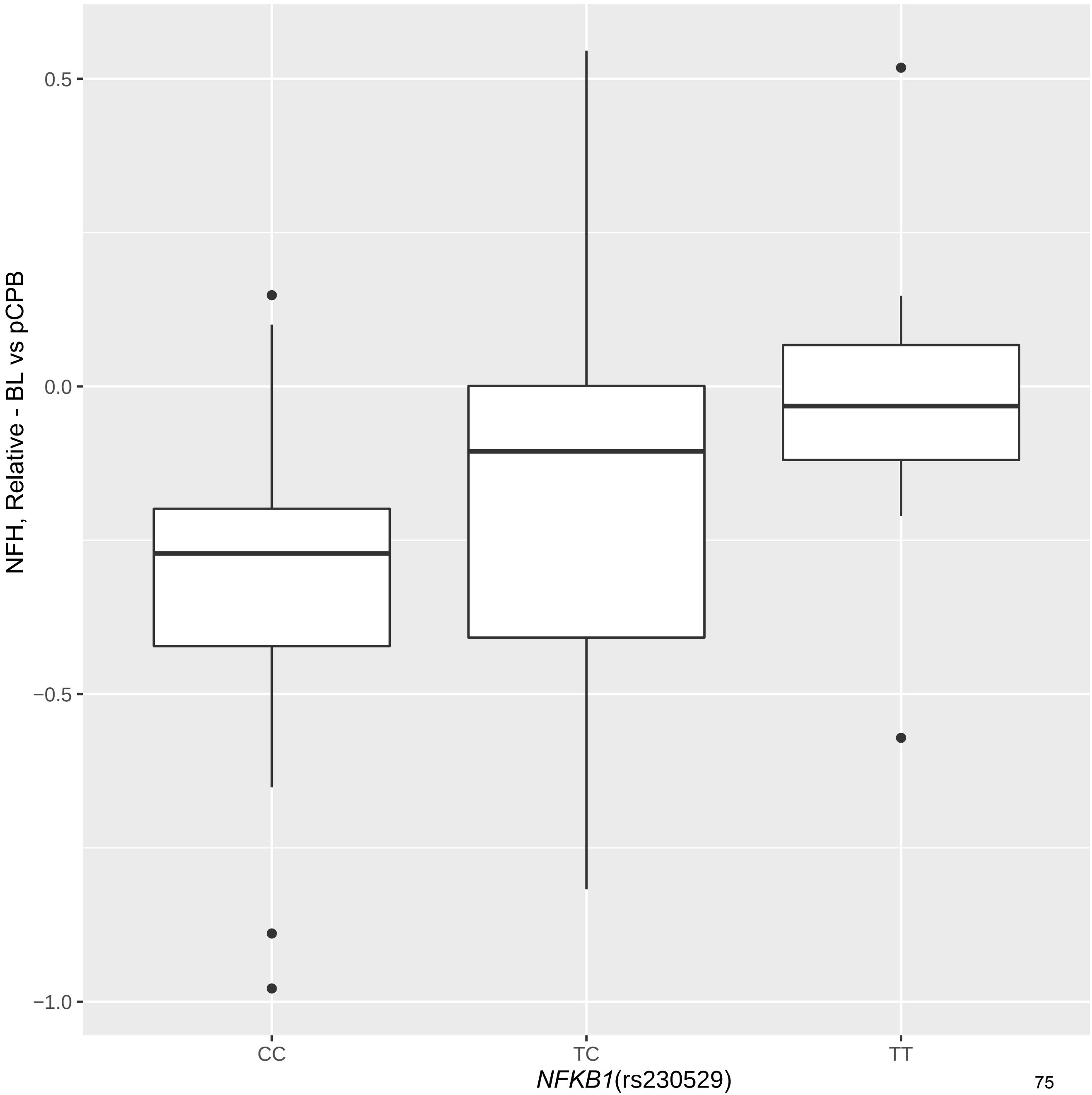
NFH, Relative - BL vs pCPB
0.5
0.0
−0.5
−1.0
CC
TC
TT
NFKB1(rs230529)

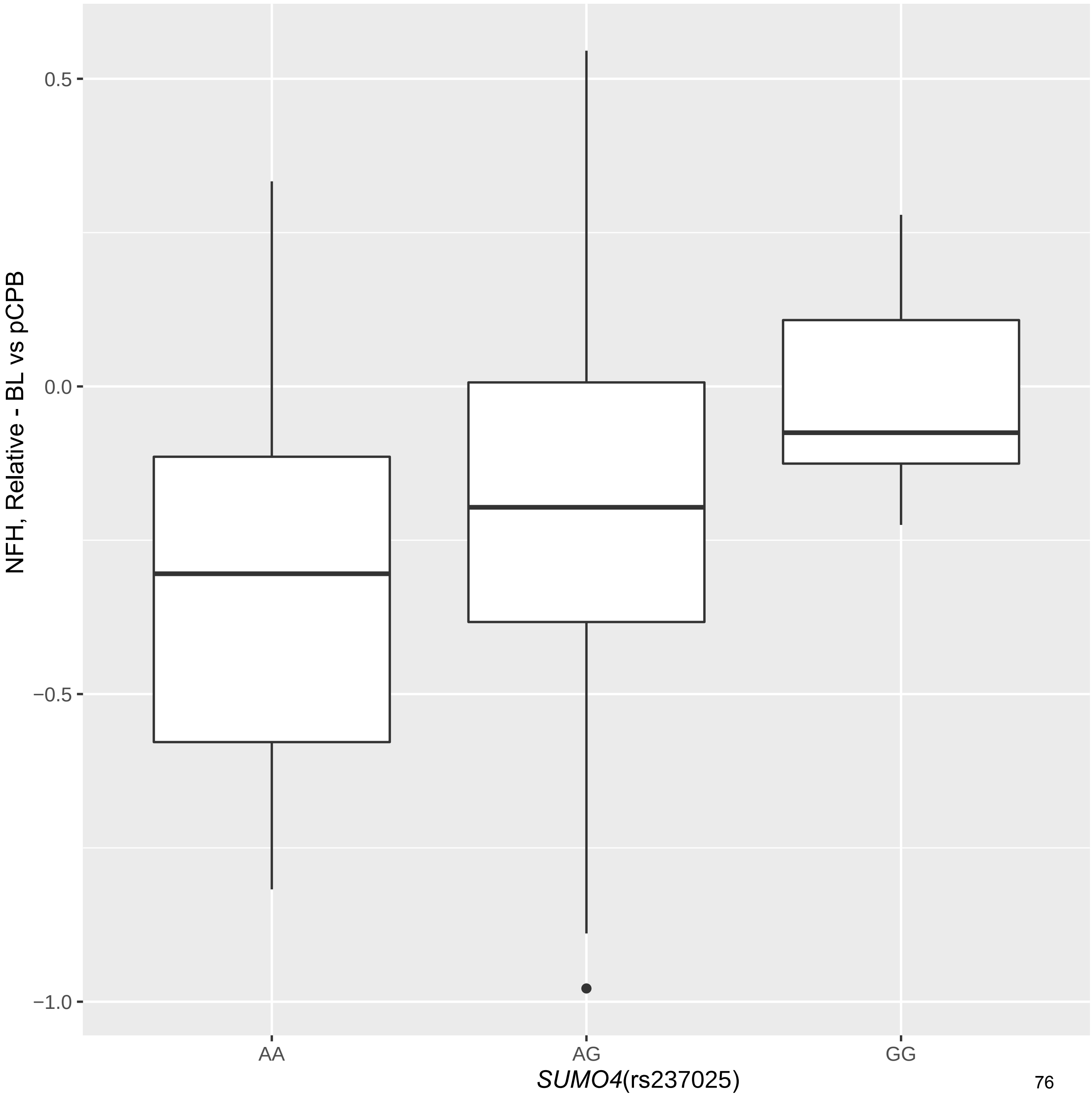

NFH, Relative - BL vs pCPB
0.5
0.0
−0.5
−1.0
AA
AG
GG
SUMO4(rs237025)

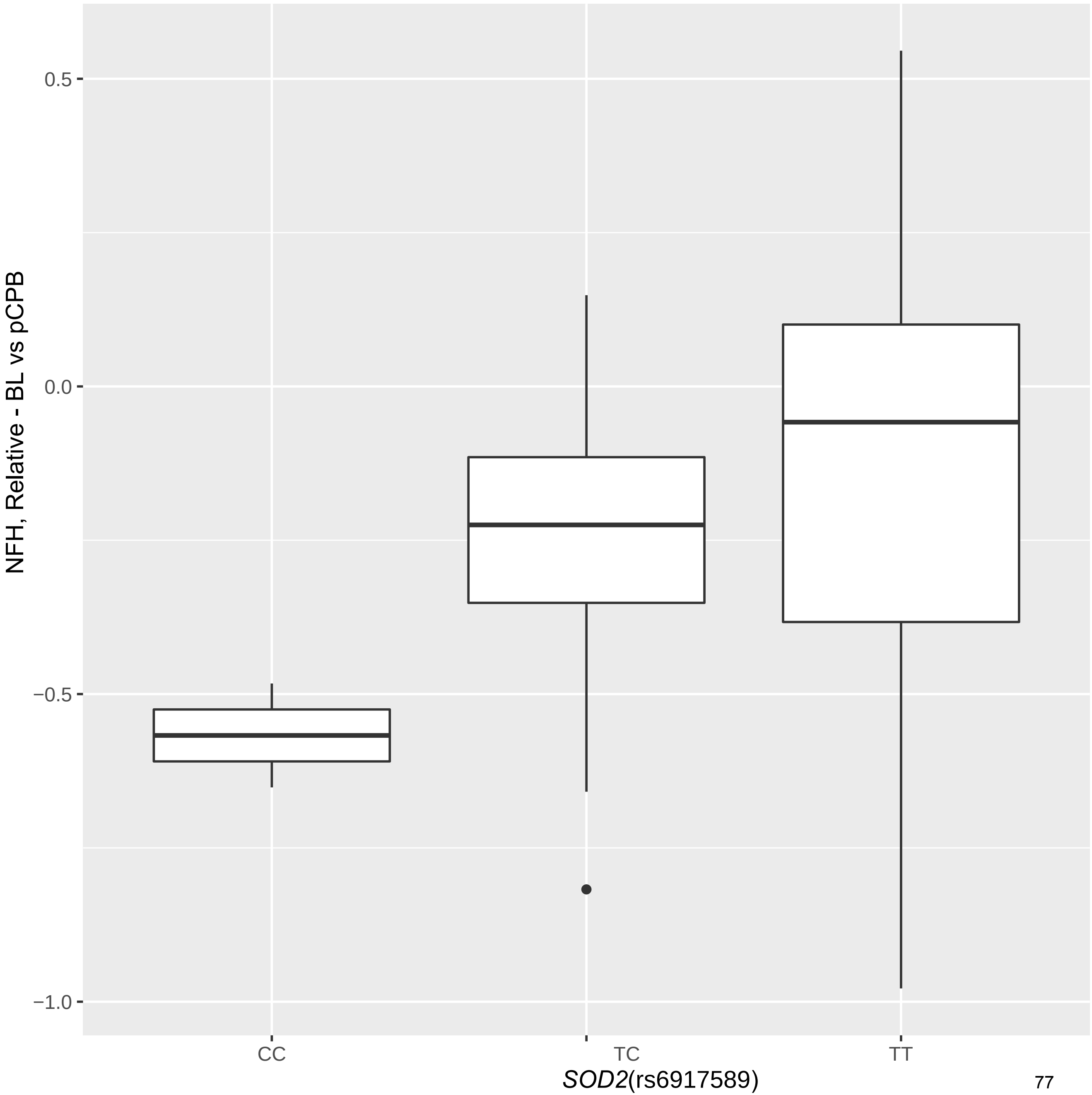

0.5
0.0
−0.5
−1.0
NFH, Relative - BL vs pCPB
CC
TC
TT
SOD2(rs6917589)

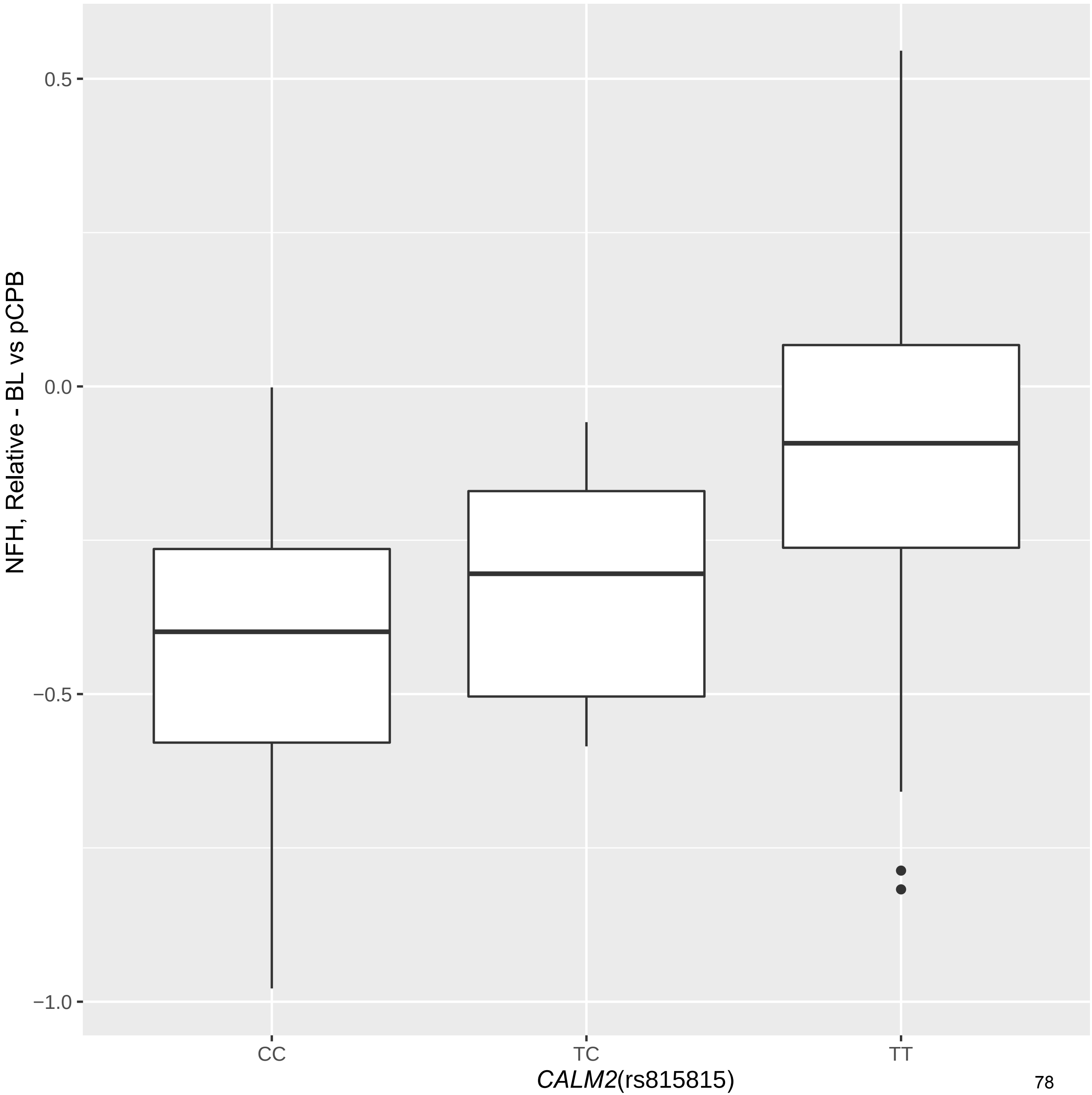
NFH, Relative - BL vs pCPB
0.5
0.0
−0.5
−1.0
CC
TC
TT
CALM2(rs815815)

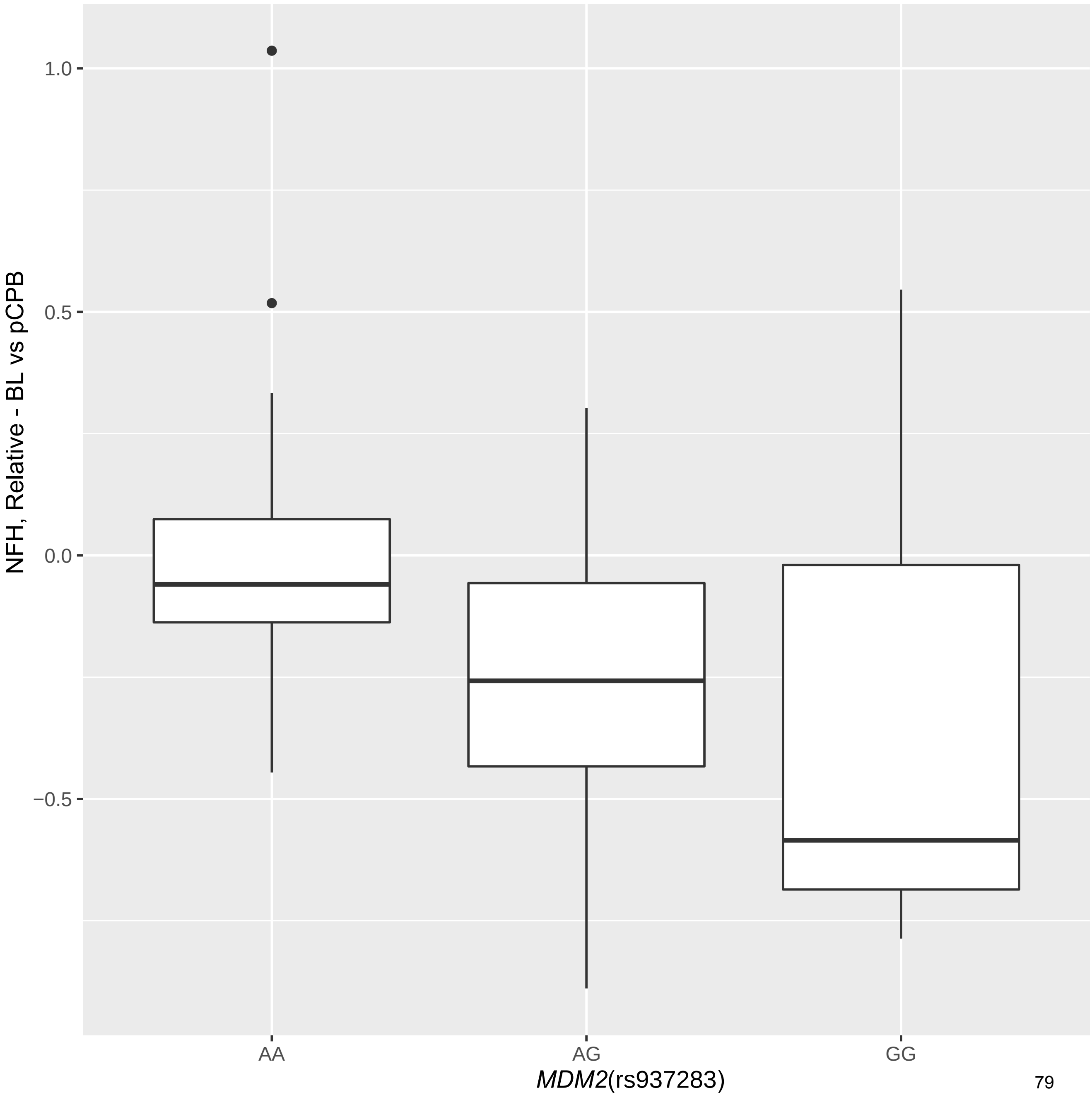

NFH, Relative - BL vs pCPB
1.0
0.5
0.0
−0.5
AA
AG
GG
MDM2(rs937283)

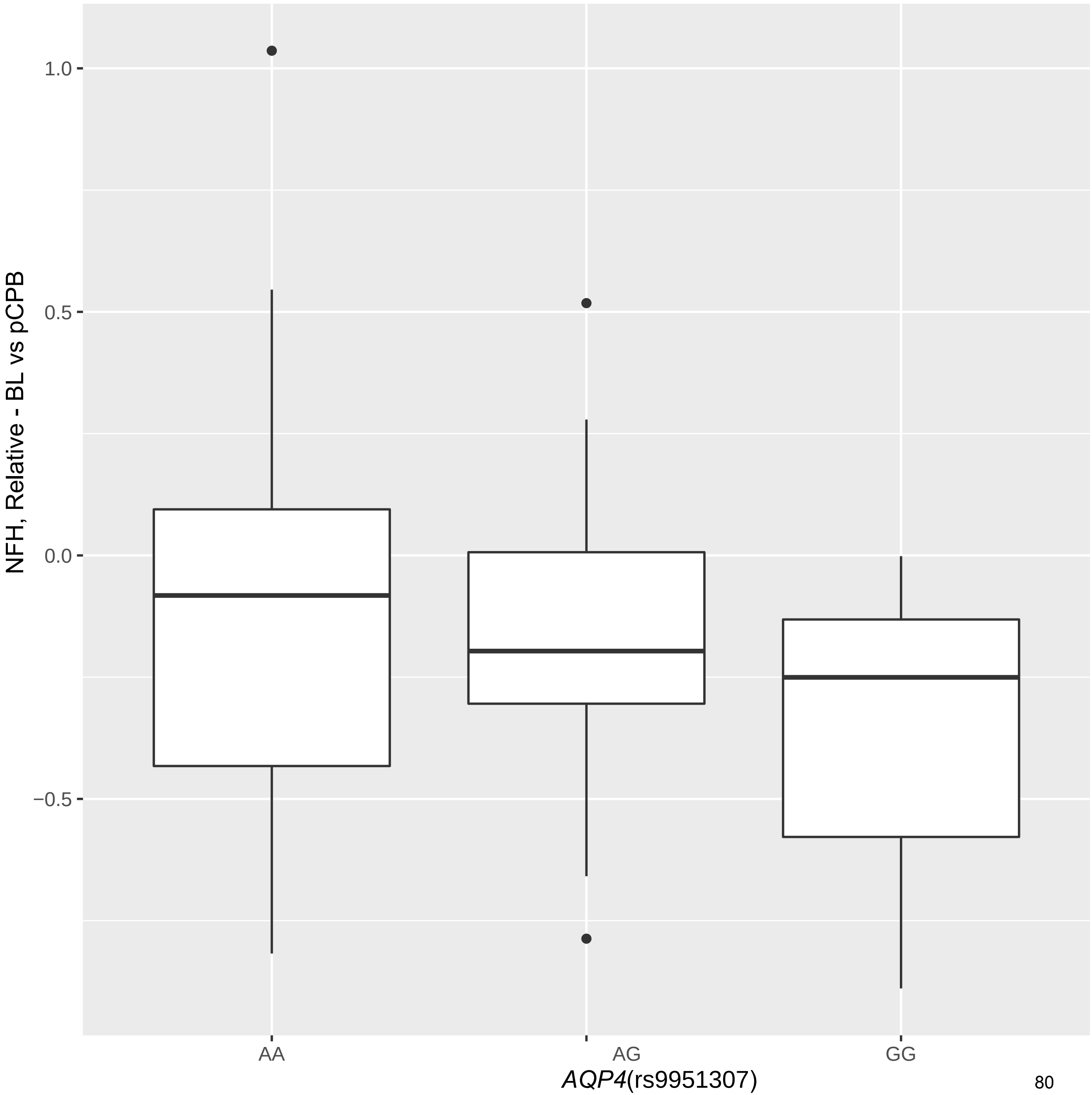
1.0
0.5
0.0
−0.5
NFH, Relative - BL vs pCPB
AA
AG
GG
AQP4(rs9951307)

Change in NFH Values-BL vs 24H (chNFH 16)

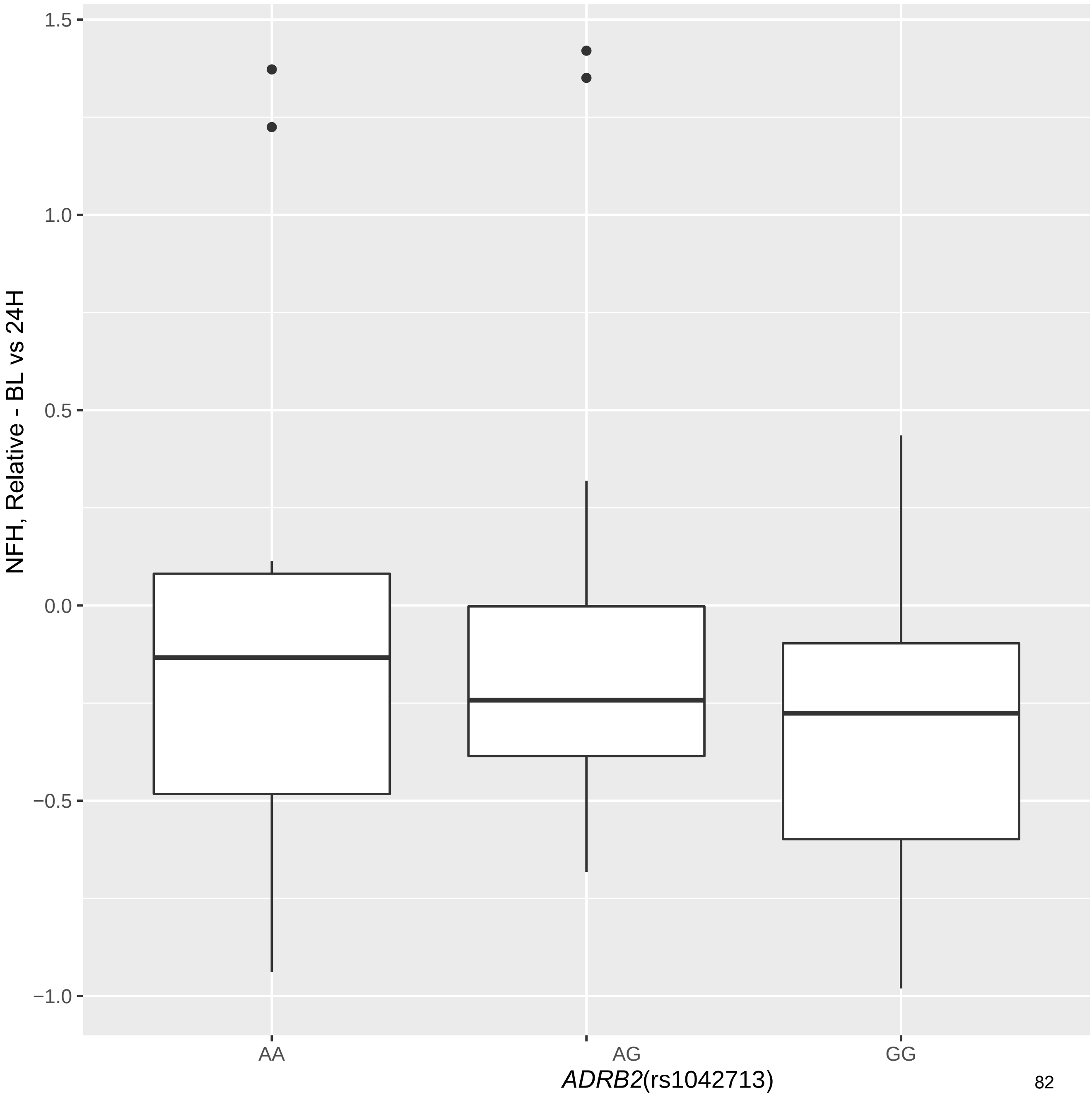
1.5
1.0
0.5
0.0
−0.5
−1.0
NFH, Relative - BL vs 24H
AA
AG
GG
ADRB2(rs1042713)

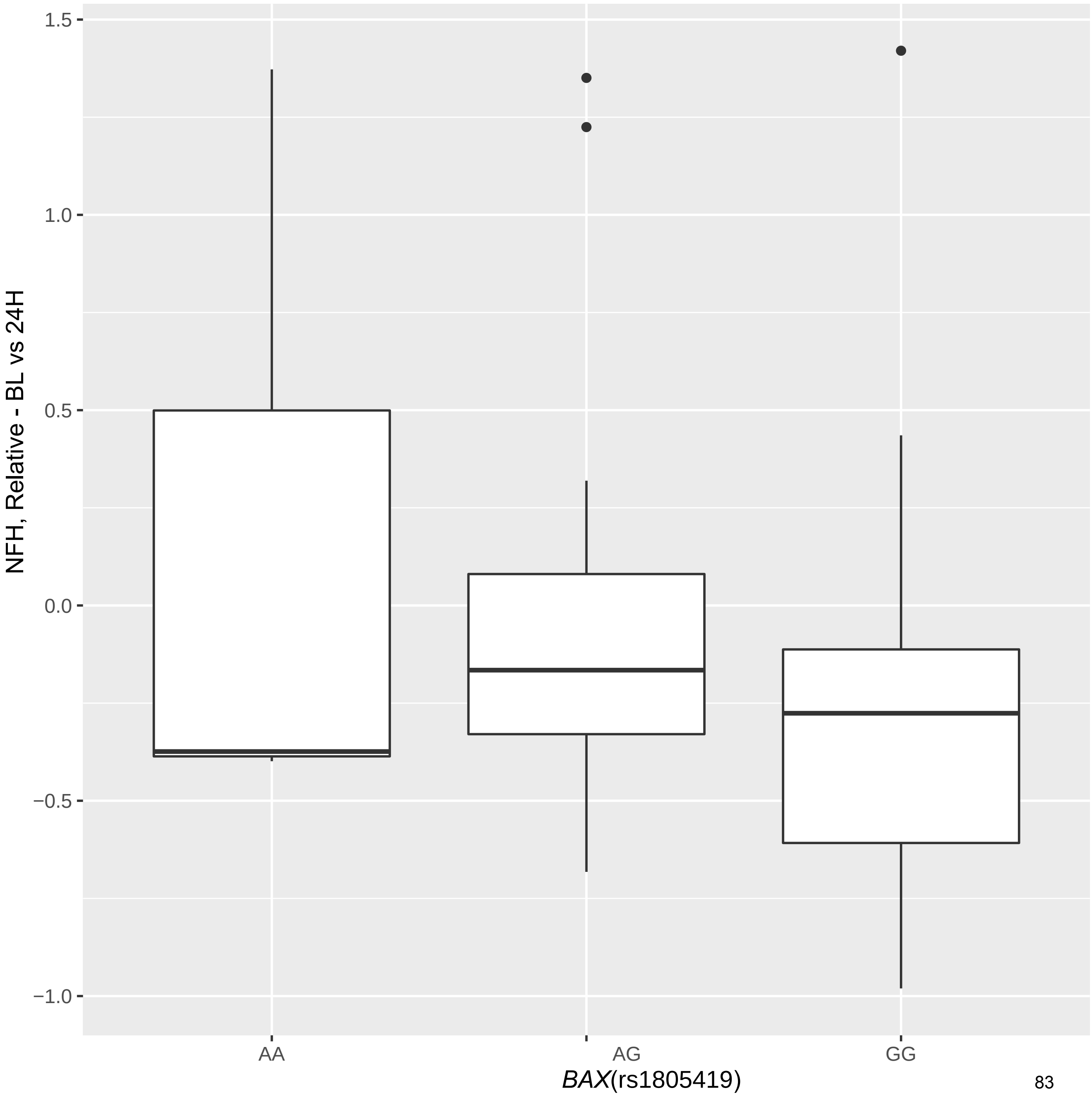
1.5
1.0
0.5
0.0
−0.5
−1.0
NFH, Relative - BL vs 24H
AA
AG
GG
BAX(rs1805419)

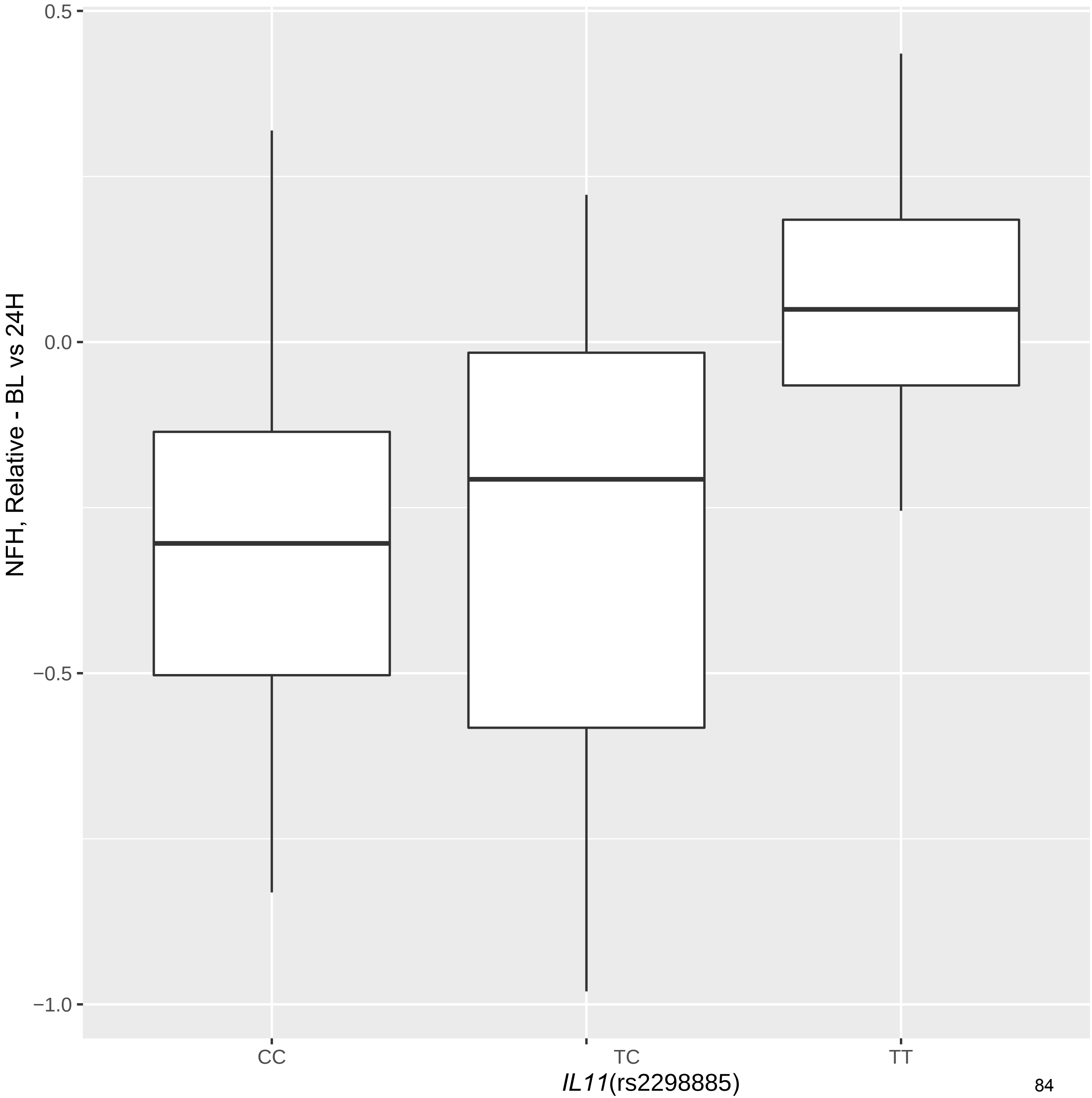

0.5
0.0
−0.5
−1.0
NFH, Relative - BL vs 24H
CC
TC
TT
IL11(rs2298885)

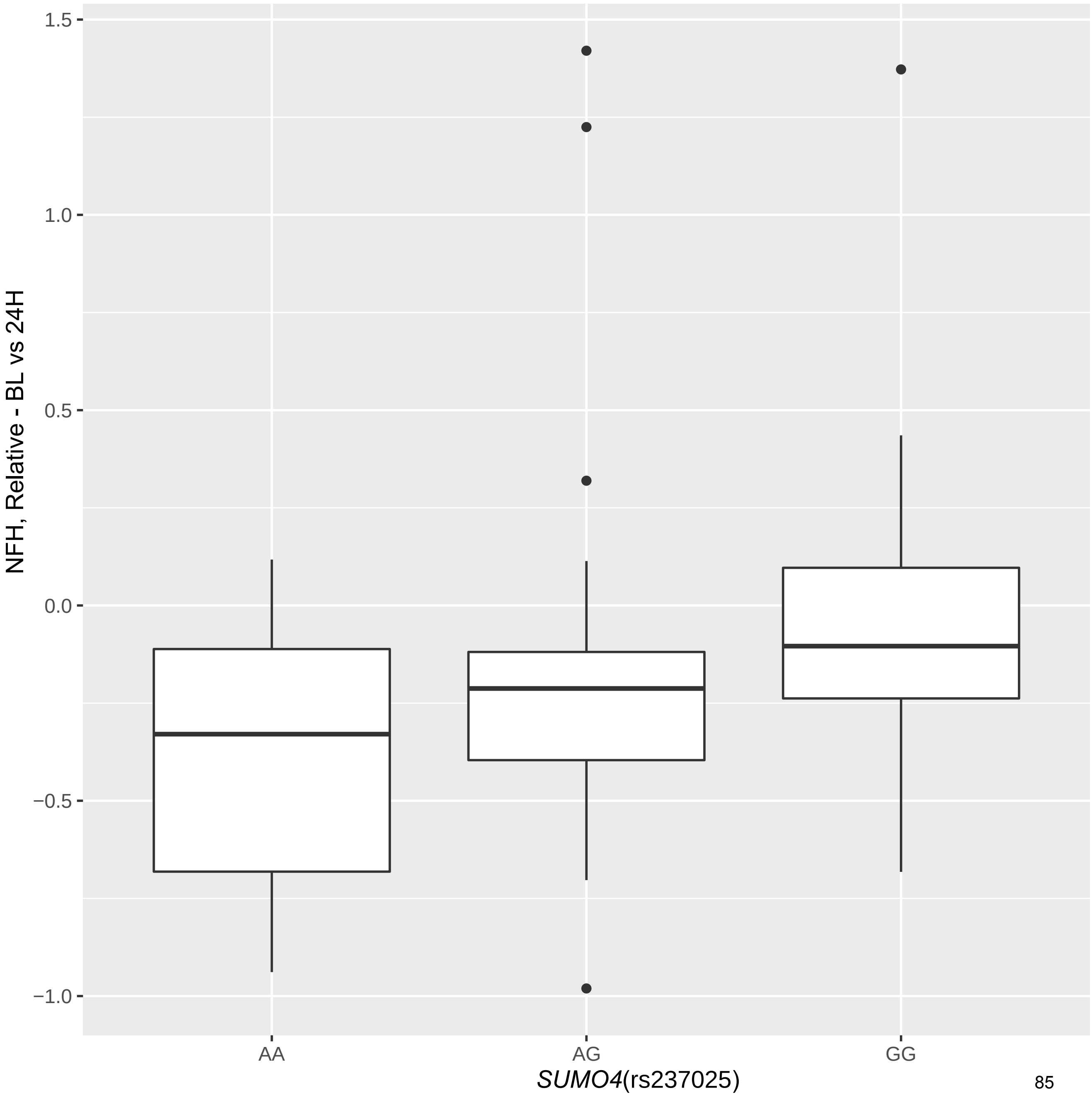
1.5
1.0
0.5
0.0
−0.5
−1.0
NFH, Relative - BL vs 24H
AA
AG
GG
SUMO4(rs237025)

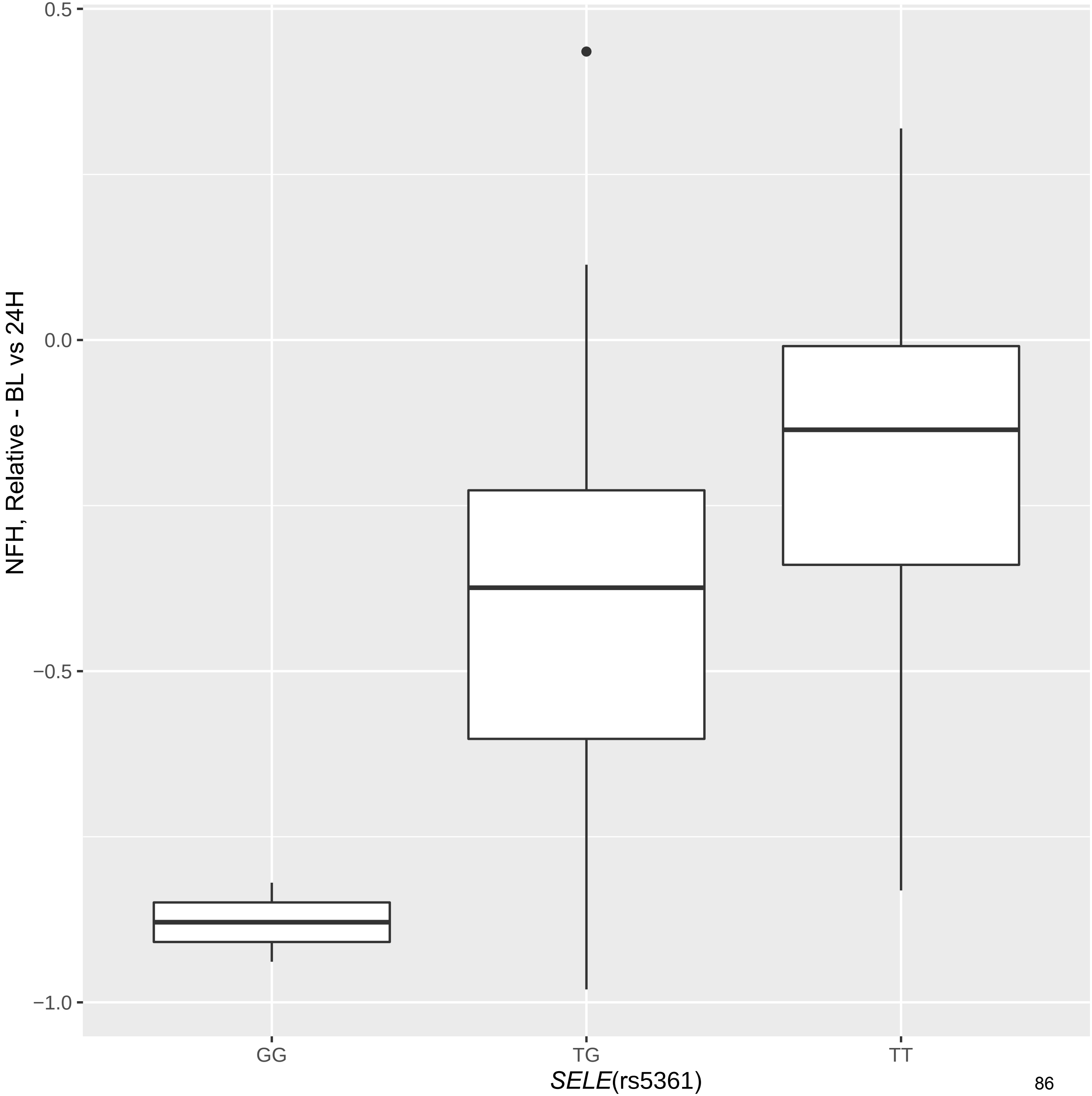

NFH, Relative - BL vs 24H
0.5
0.0
−0.5
−1.0
GG
TG
TT
SELE(rs5361)

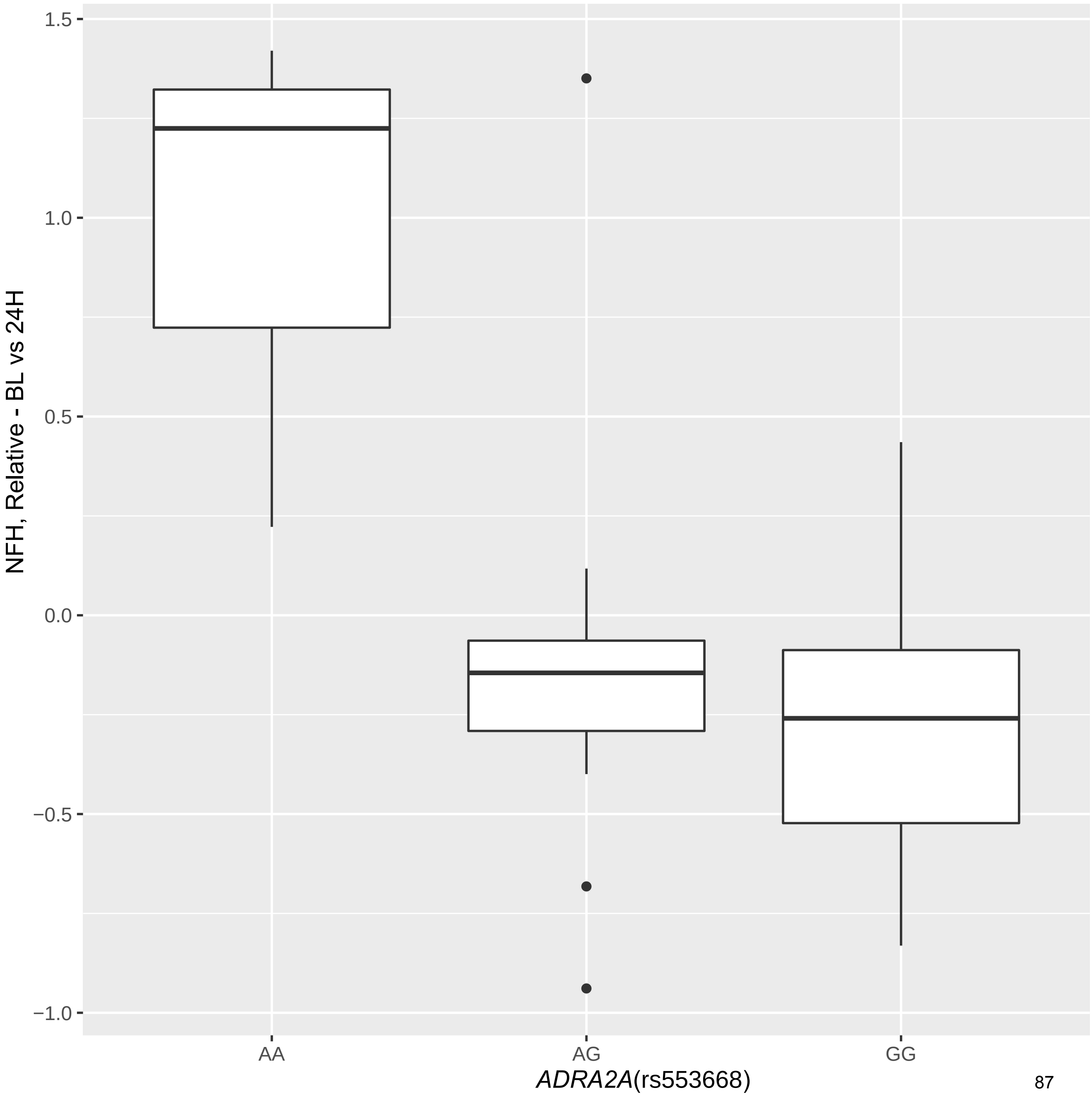

1.5
1.0
0.5
0.0
−0.5
−1.0
NFH, Relative - BL vs 24H
AA
AG
GG
ADRA2A(rs553668)

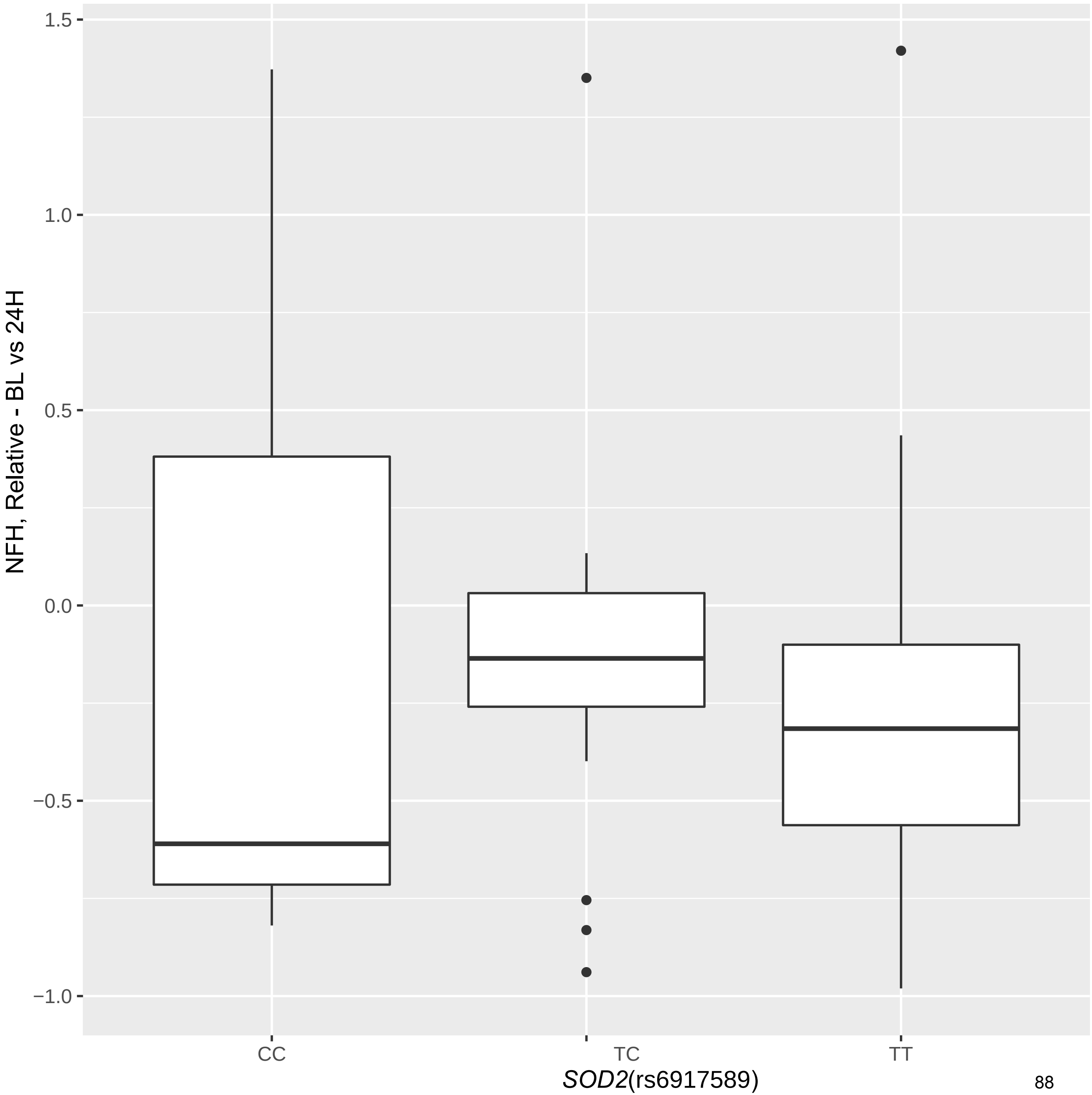

NFH, Relative - BL vs 24H
1.5
1.0
0.5
0.0
−0.5
−1.0
CC
TC
TT
SOD2(rs6917589)

Change in S100β Values-BL vs aCPB (chS100B 12)

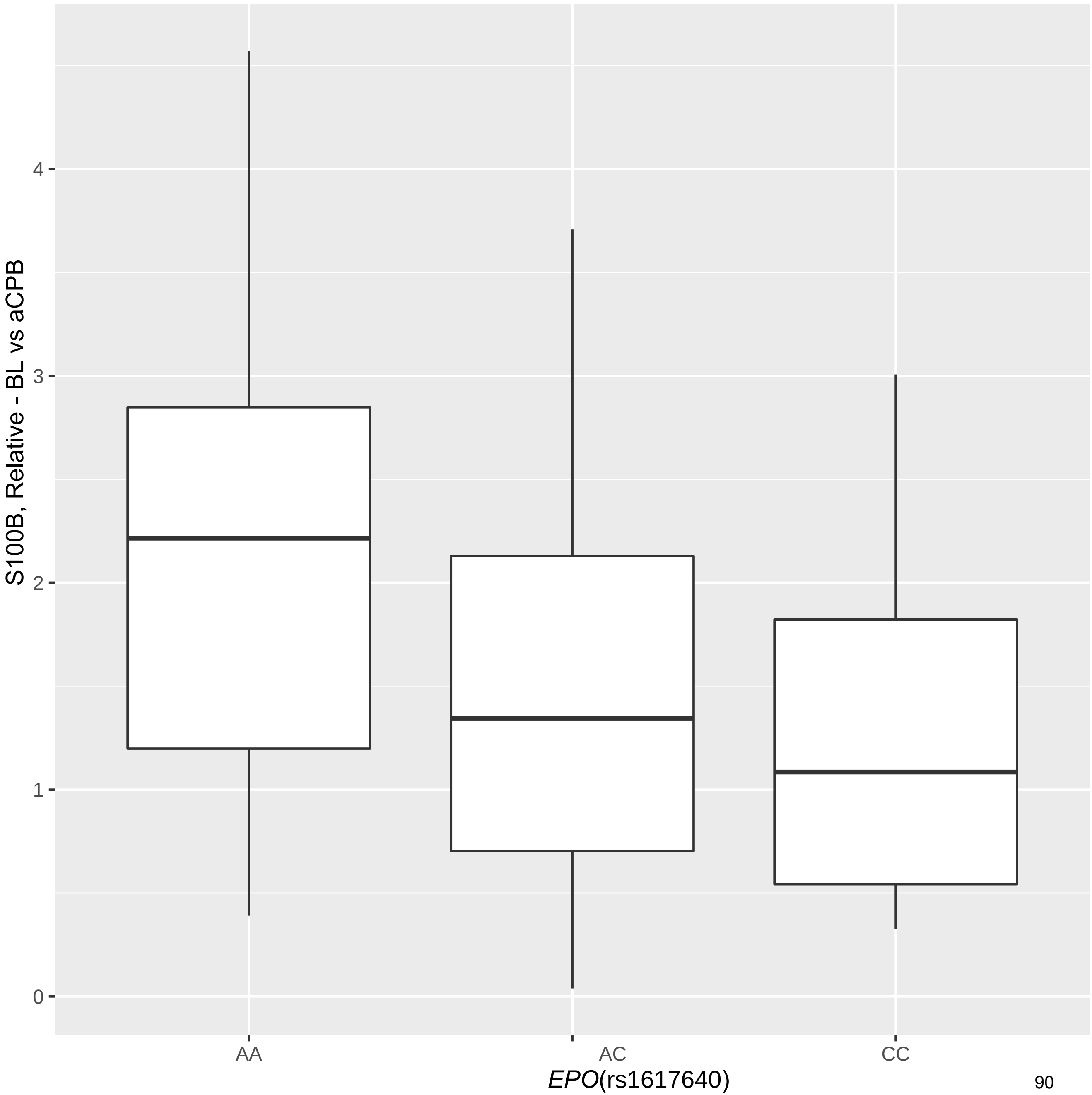
S100B, Relative - BL vs aCPB
0
1
2
3
4
AA
AC
CC
EPO(rs1617640)

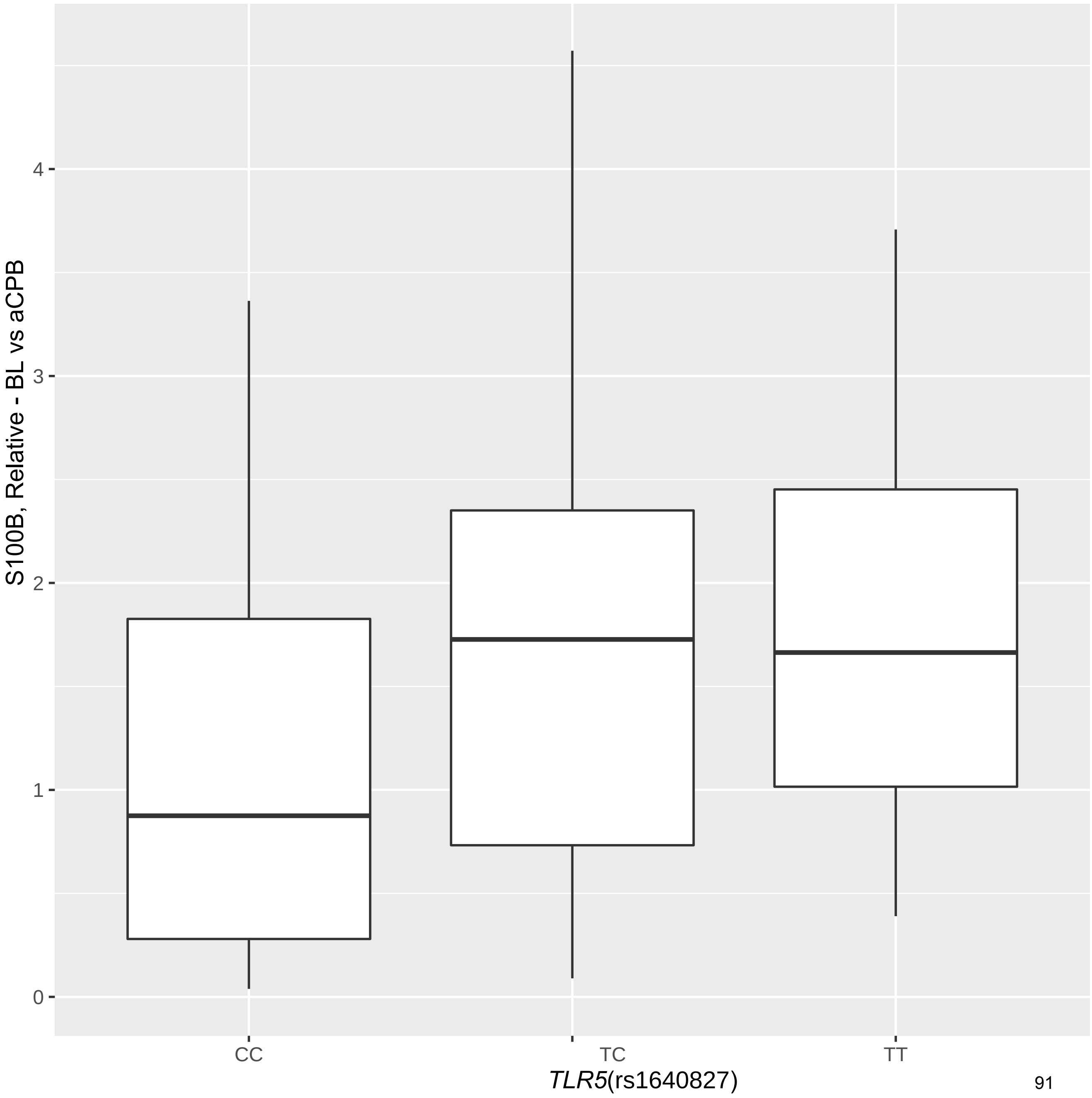

S100B, Relative - BL vs aCPB
0
1
2
3
4
CC
TC
TT
TLR5(rs1640827)

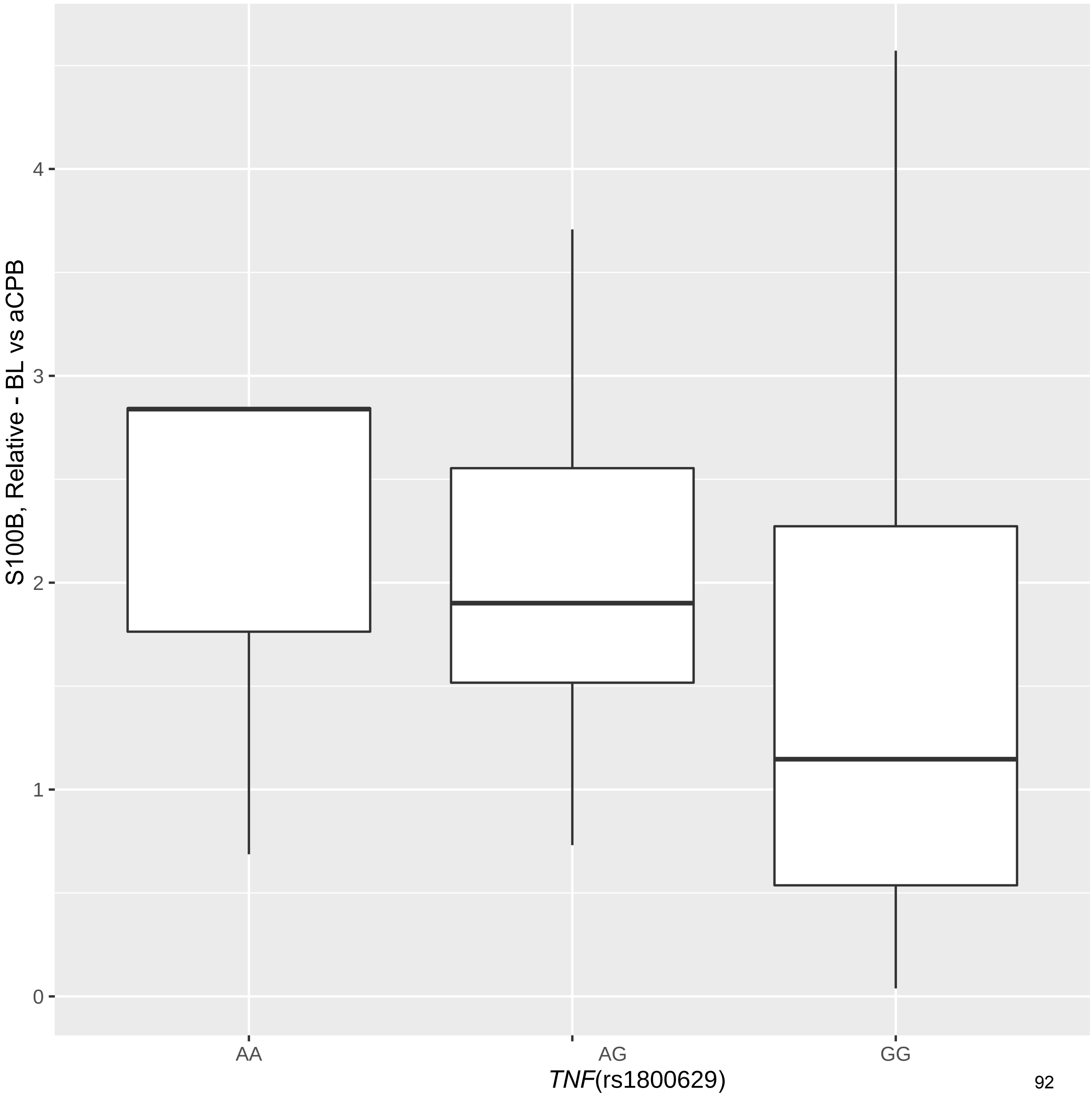
4
3
2
1
0
S100B, Relative - BL vs aCPB
AA
AG
GG
TNF(rs1800629)

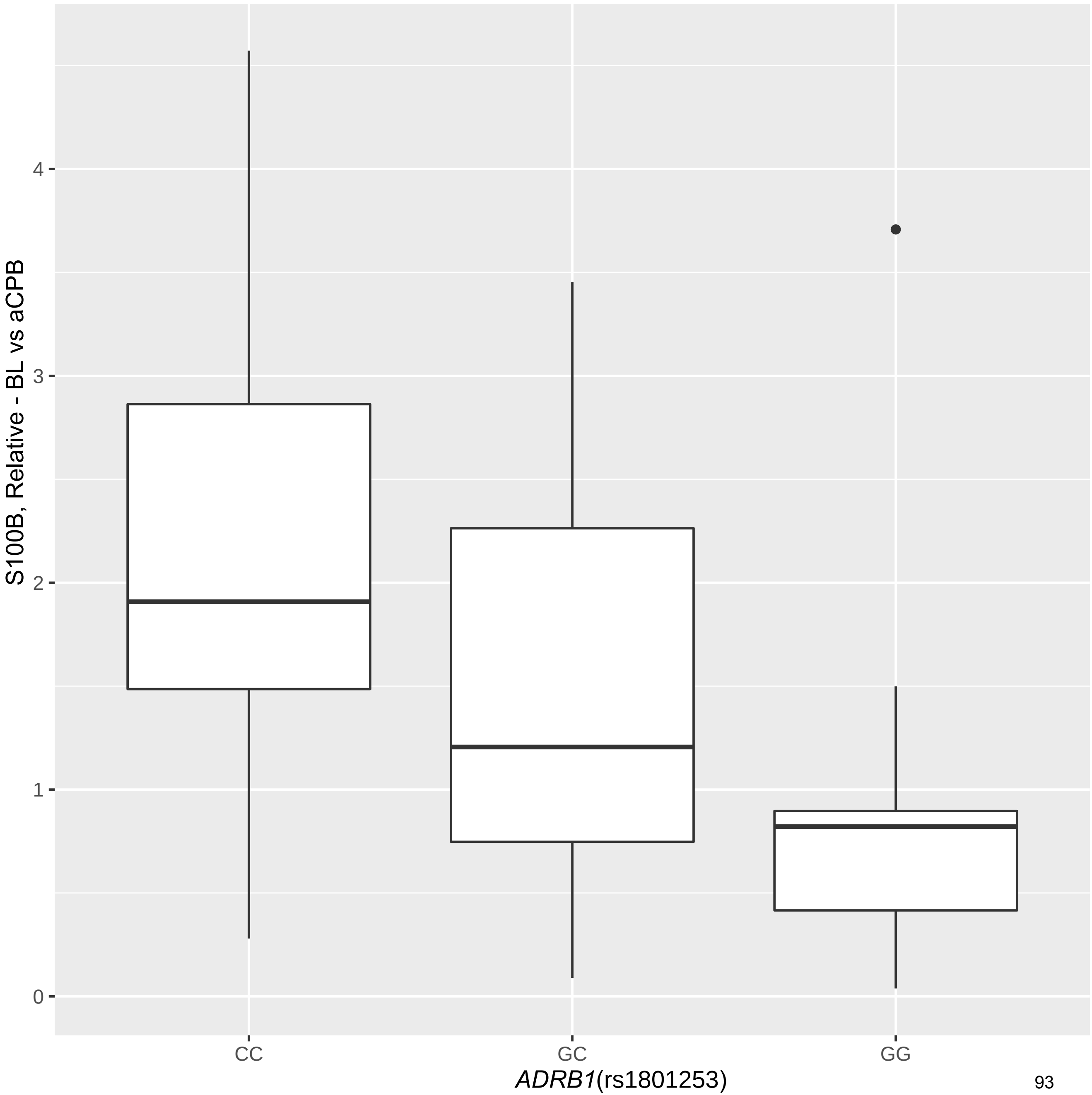
S100B, Relative - BL vs aCPB
0
1
2
3
4
CC
GC
GG
ADRB1(rs1801253)

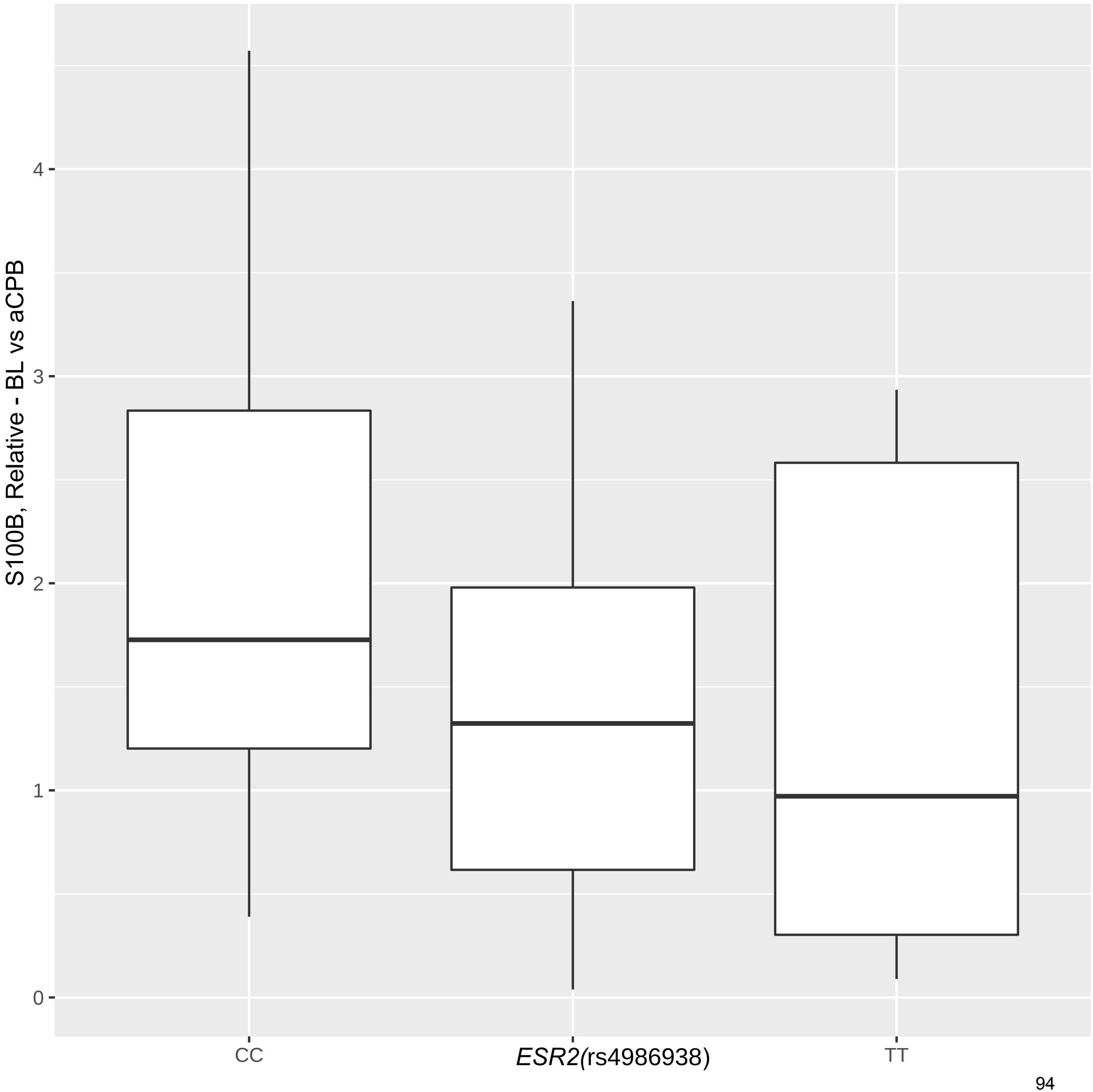
S100B, Relative - BL vs aCPB
4
3
2
1
0
CC
TT
ESR2(rs4986938)

Change in S100β Values-BL vs pCPB (chS100B 15)

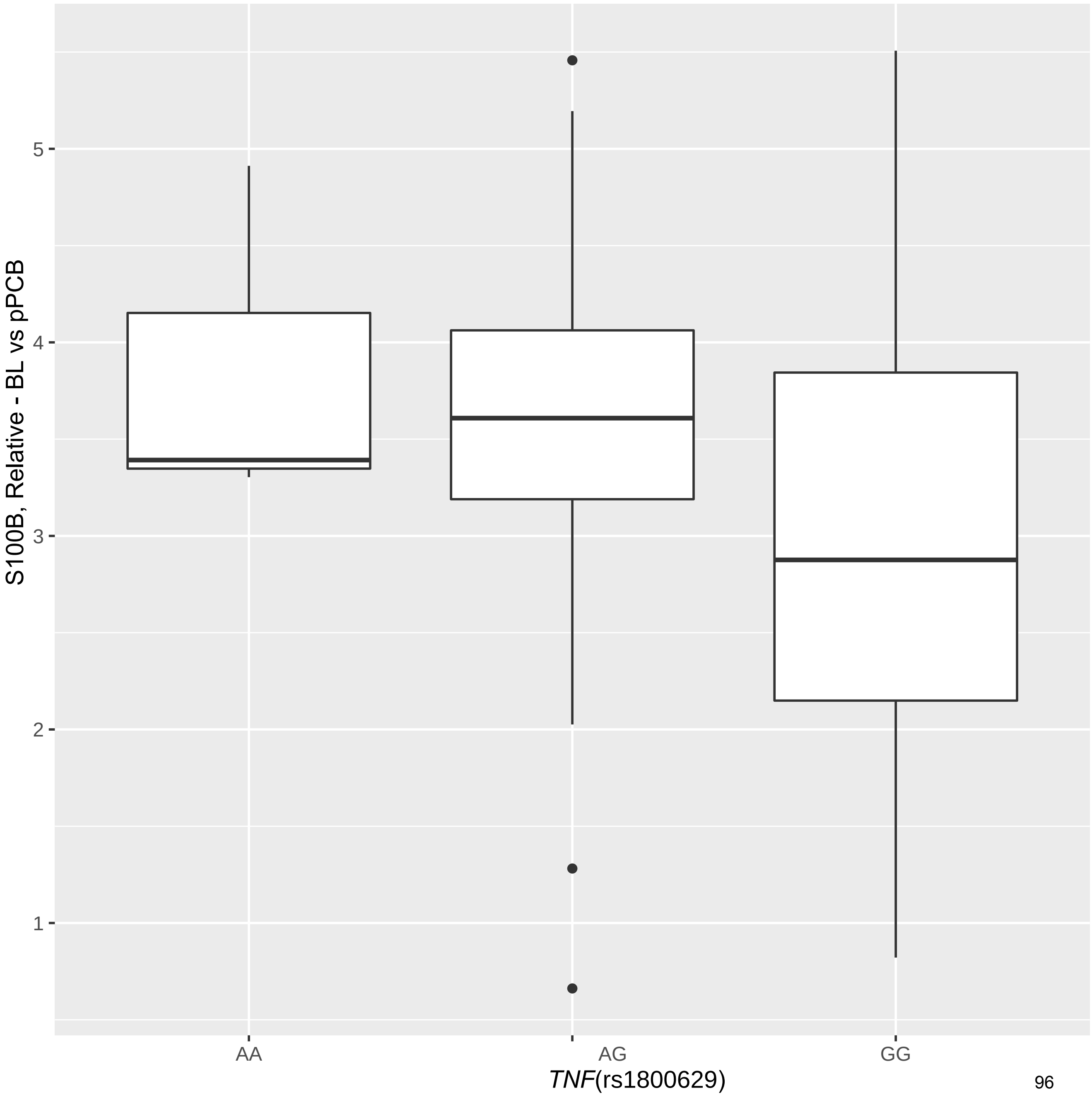
S100B, Relative - BL vs pPCB
5
4
3
2
1
AA
AG
GG
TNF(rs1800629)

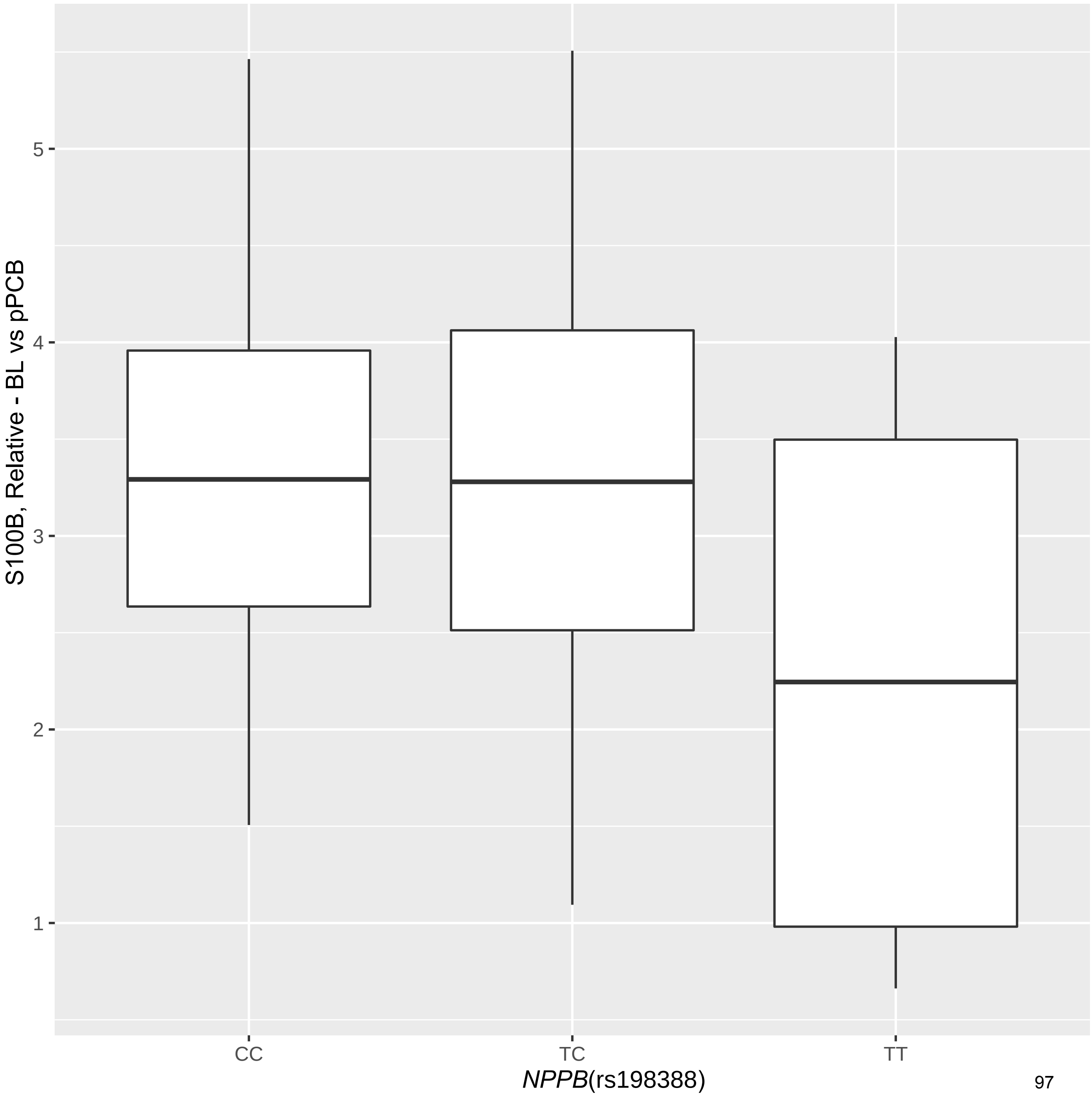
S100B, Relative - BL vs pPCB
5
4
3
2
1
CC
TC
TT
NPPB(rs198388)

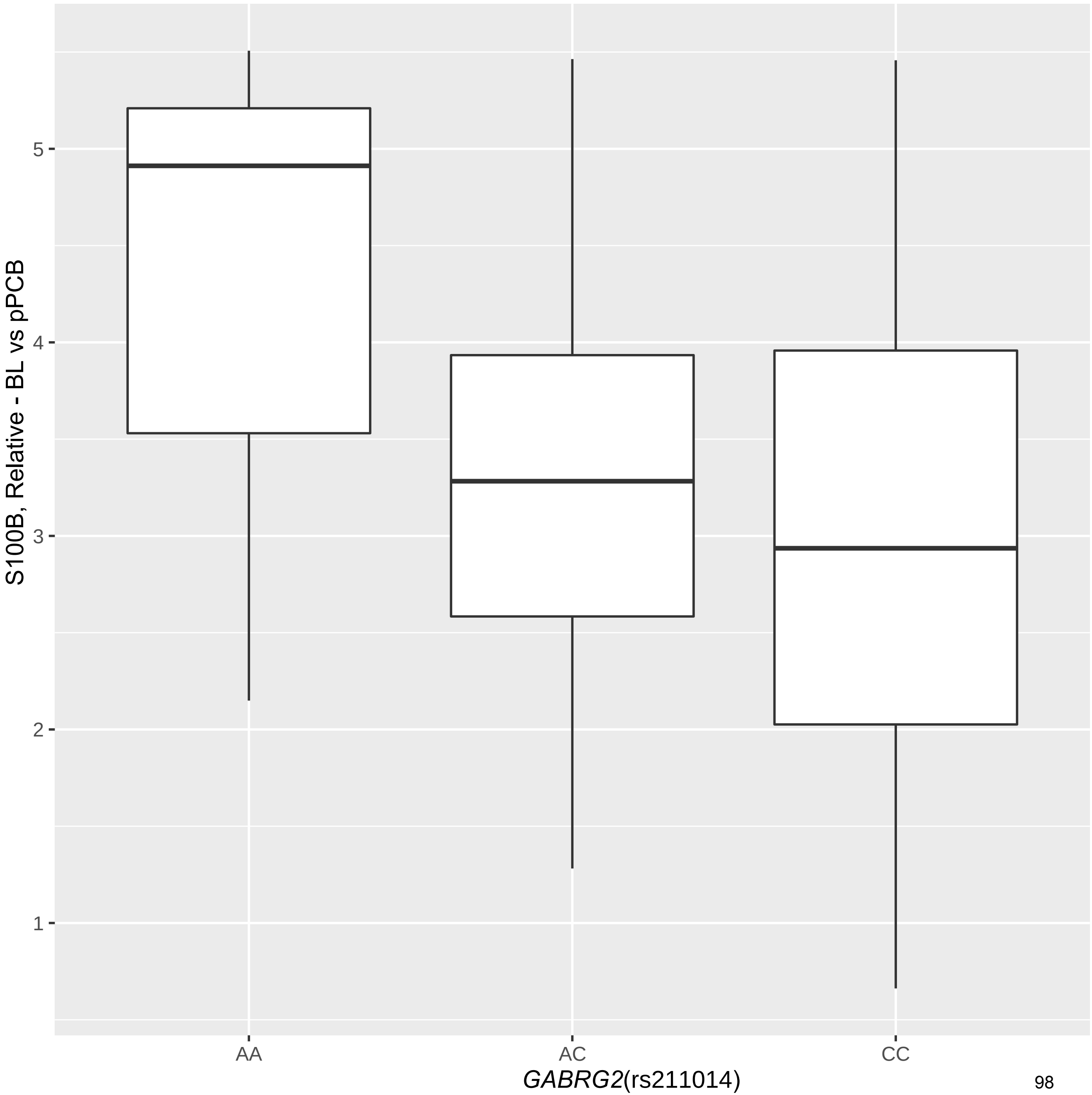

S100B, Relative - BL vs pPCB
5
4
3
2
1
AA
AC
CC
GABRG2(rs211014)

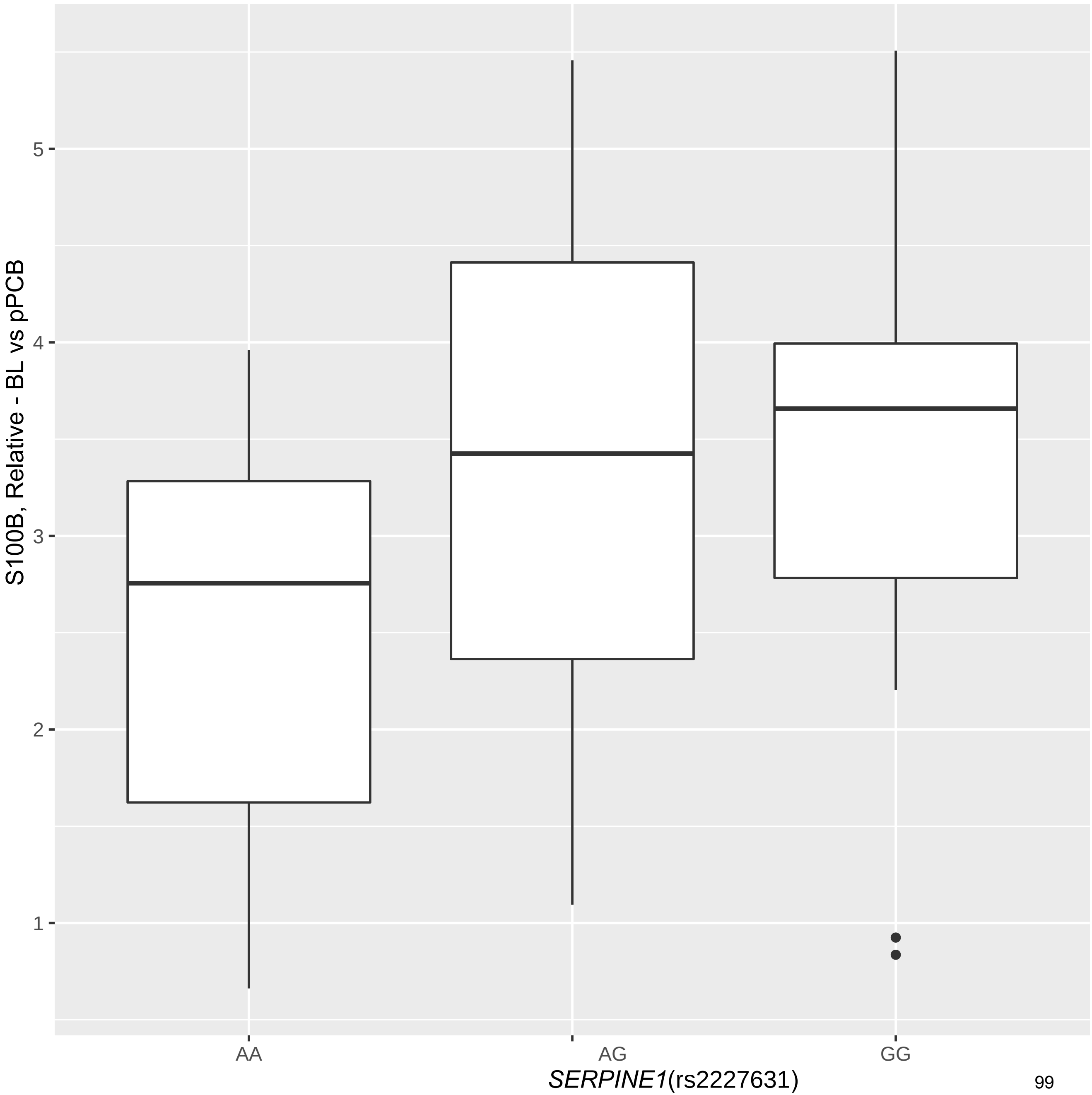

5
4
3
2
1
S100B, Relative - BL vs pPCB
AA
AG
GG
SERPINE1(rs2227631)

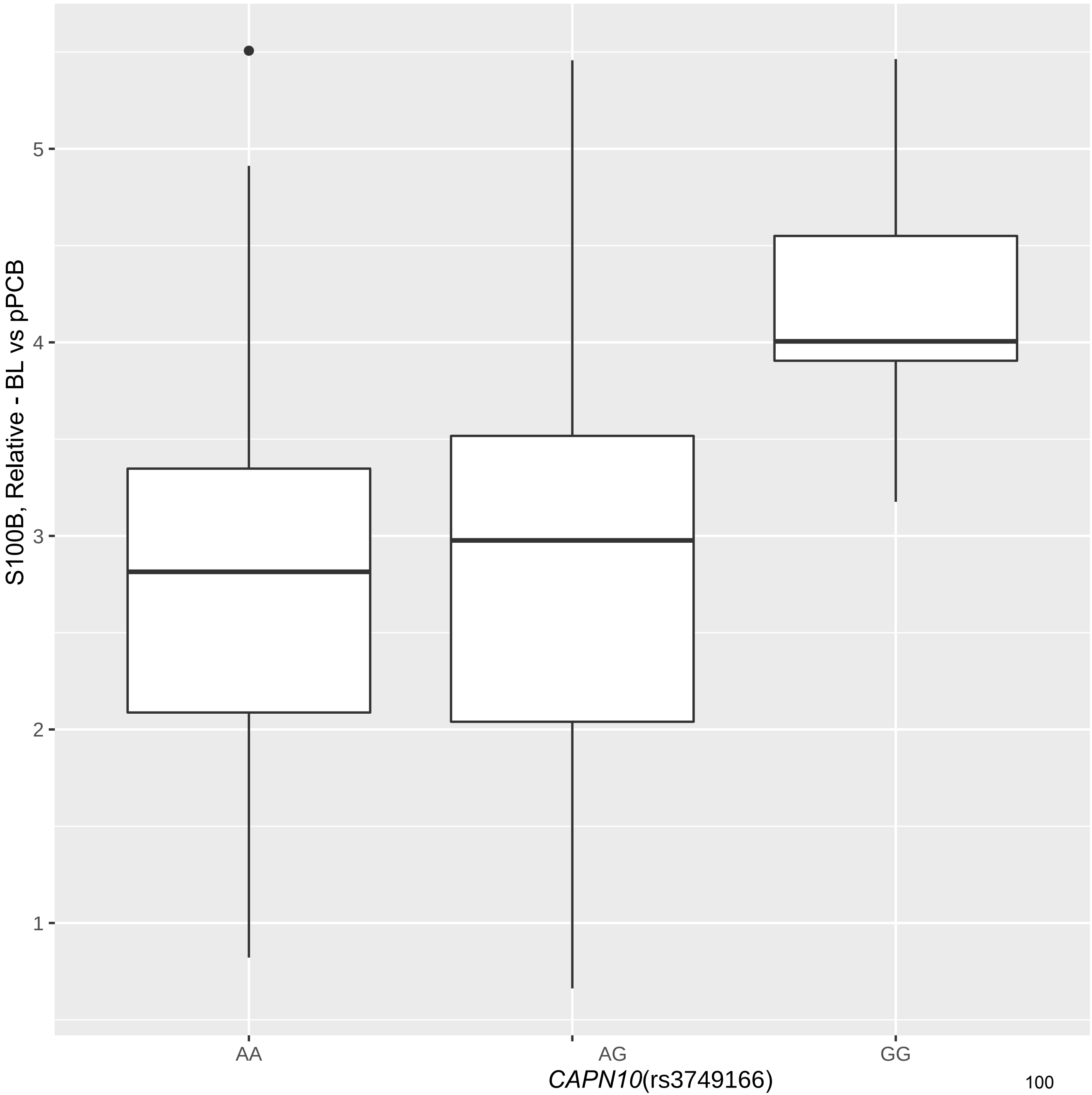
S100B, Relative - BL vs pPCB
5
4
3
2
1
AA
AG
GG
CAPN10(rs3749166)

Change in S100β Values-BL vs 24H (chS100B 16)

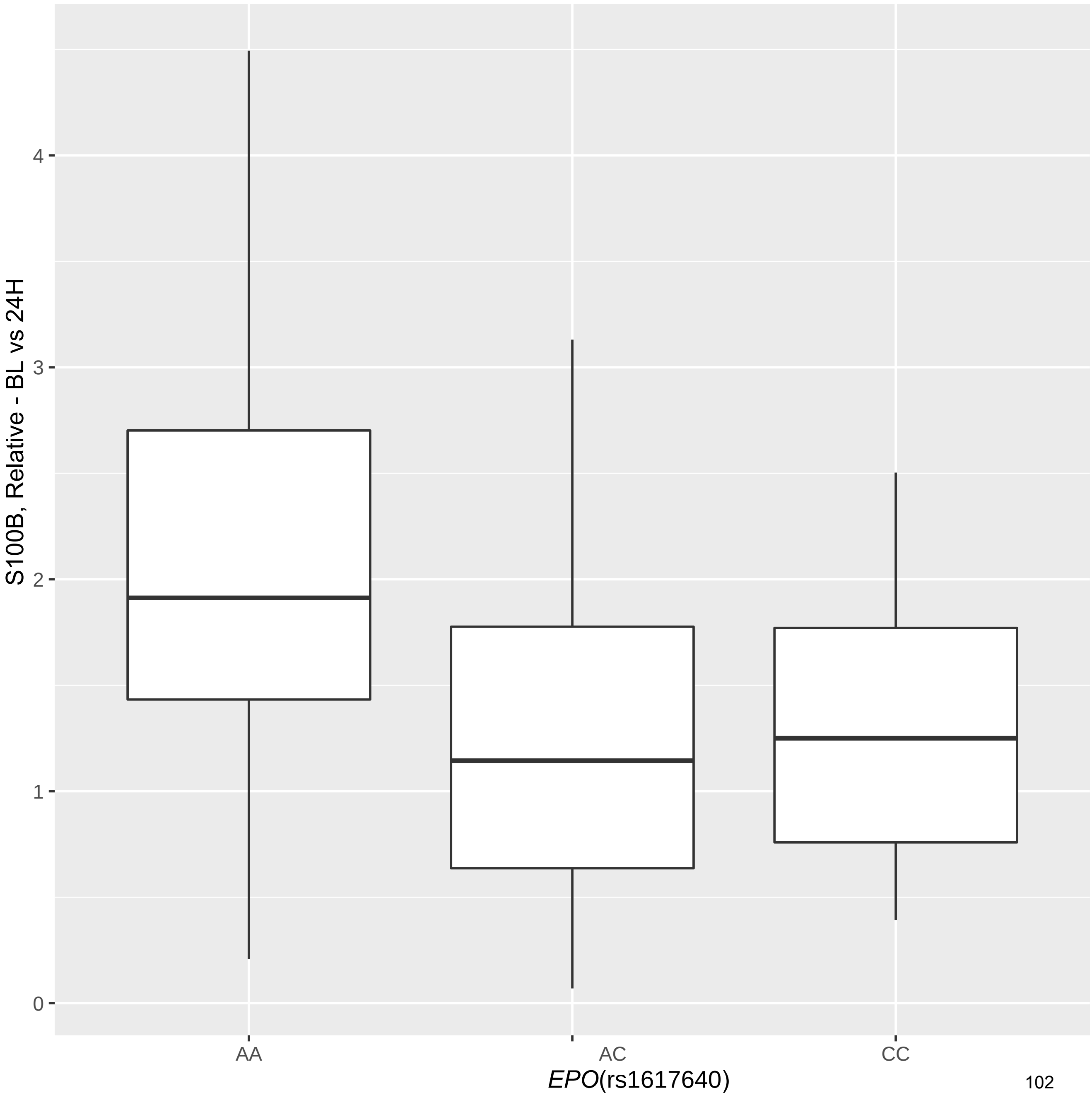
S100B, Relative - BL vs 24H
4
3
2
1
0
AA
AC
CC
EPO(rs1617640)

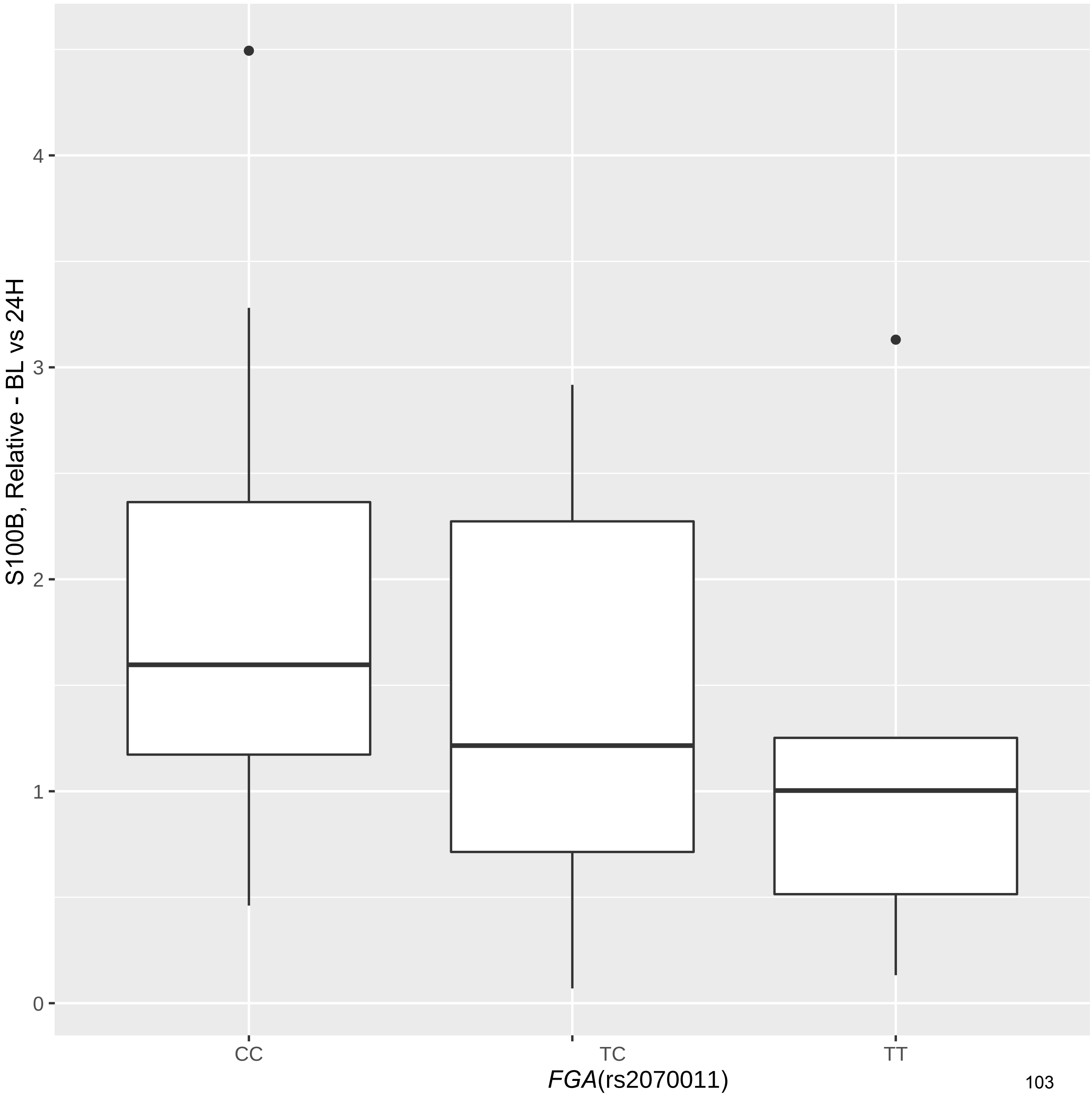
S100B, Relative - BL vs 24H
0
1
2
3
4
CC
TC
TT
FGA(rs2070011)

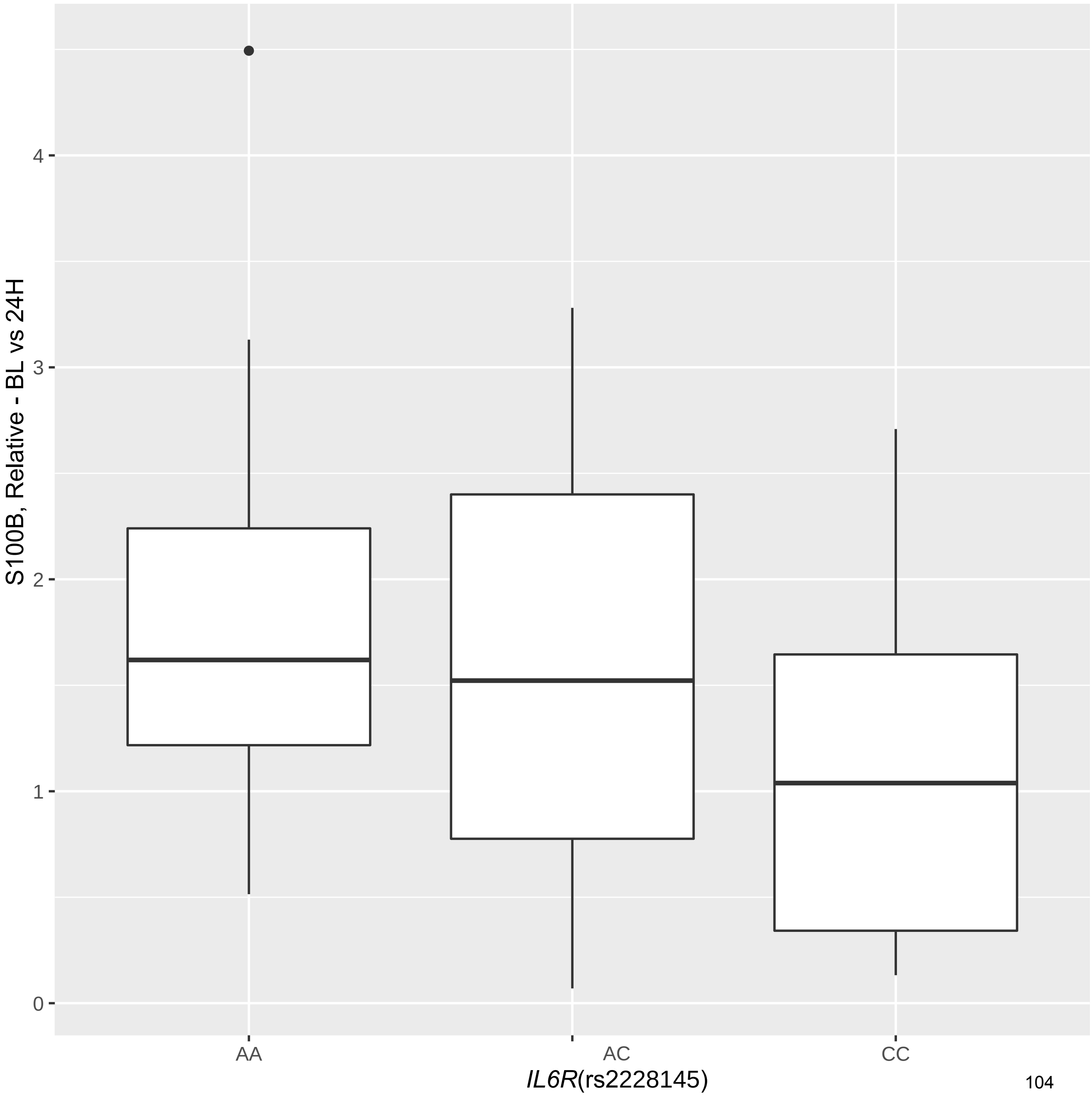
S100B, Relative - BL vs 24H
0
1
2
3
4
AA
AC
CC
IL6R(rs2228145)

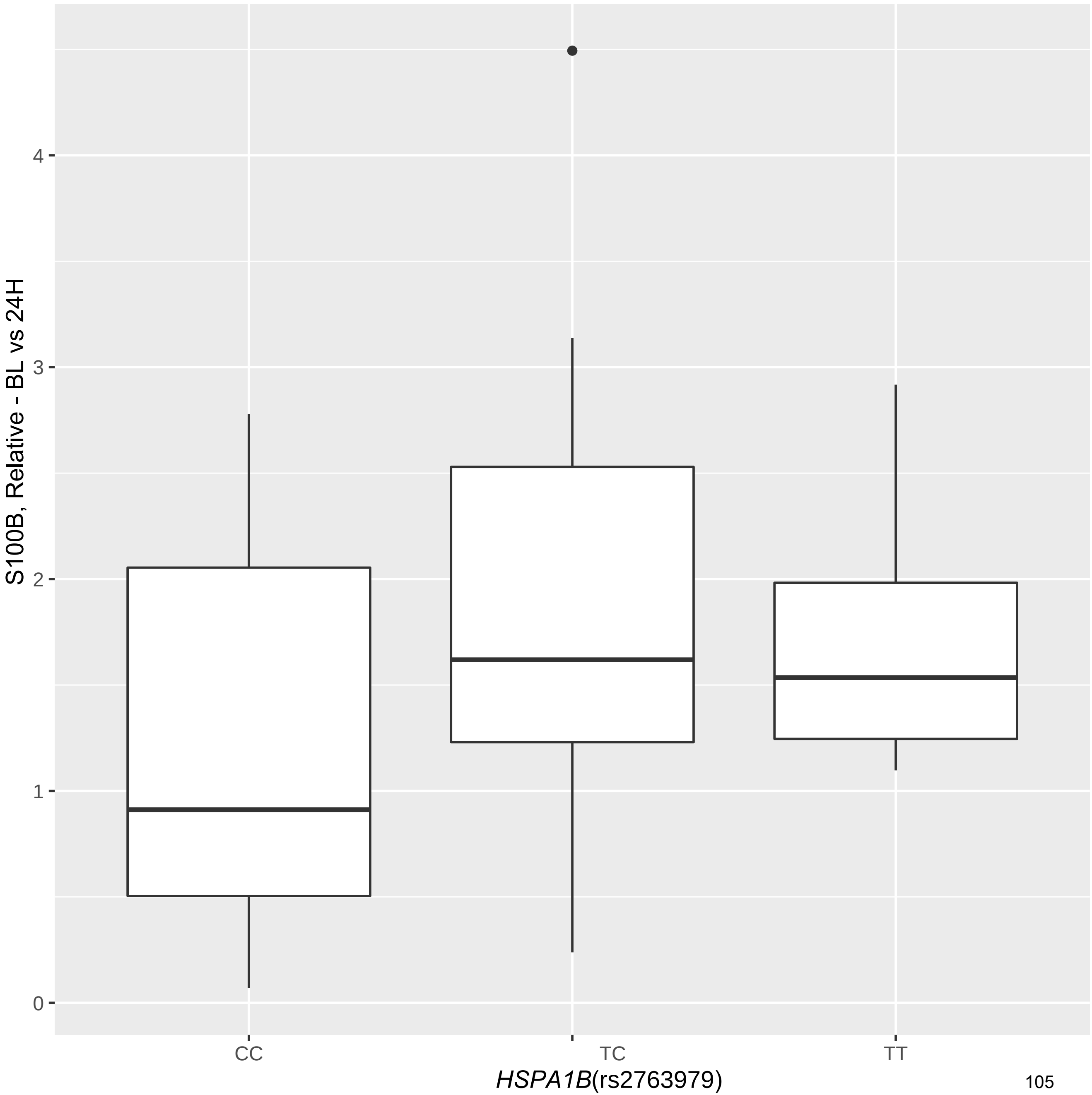
S100B, Relative - BL vs 24H
0
1
2
3
4
CC
TC
TT
HSPA1B(rs2763979)

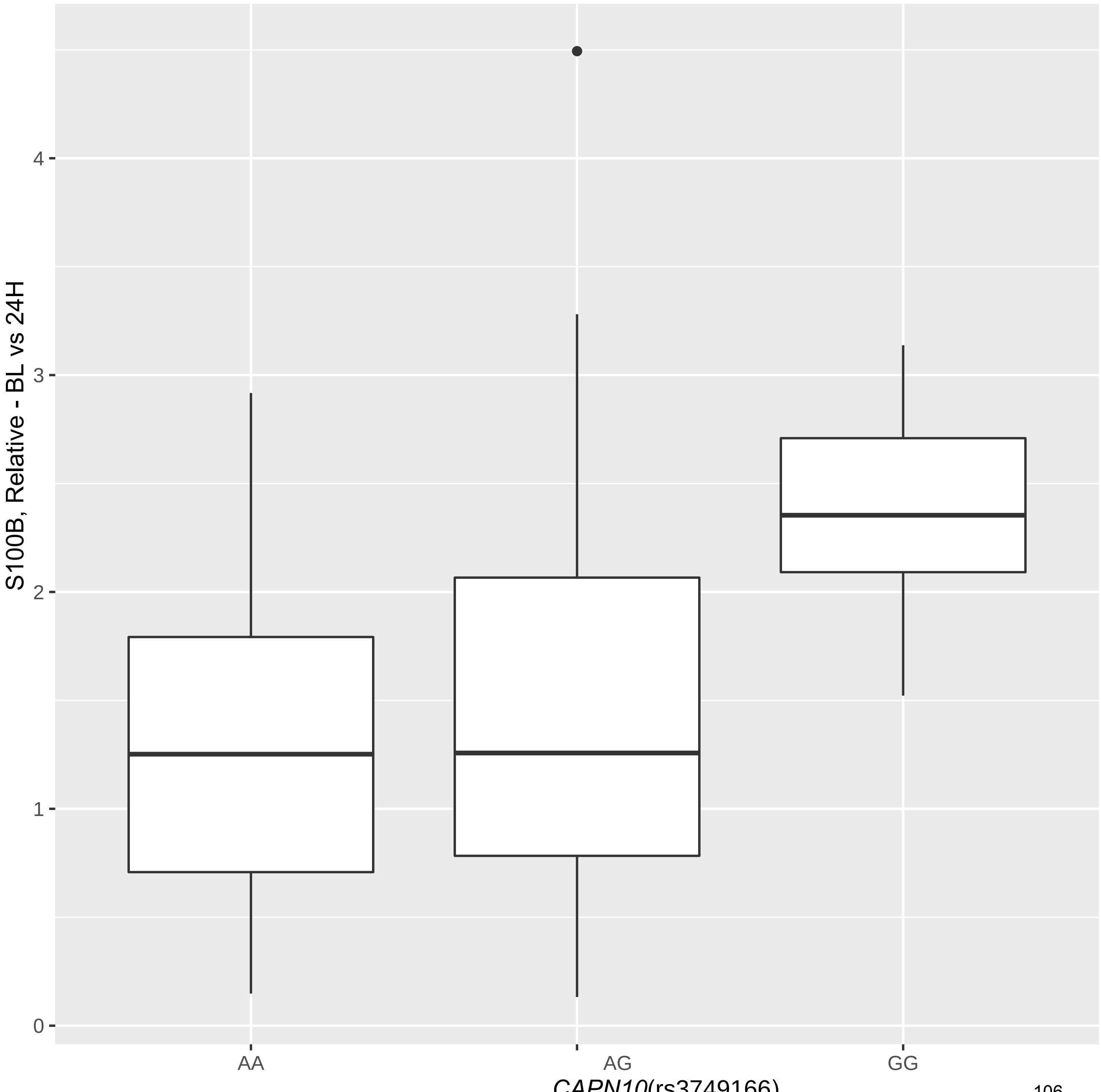
S100B, Relative - BL vs 24H
0
1
2
3
4
AA
AG
GG
CAPN10(rs3749166)

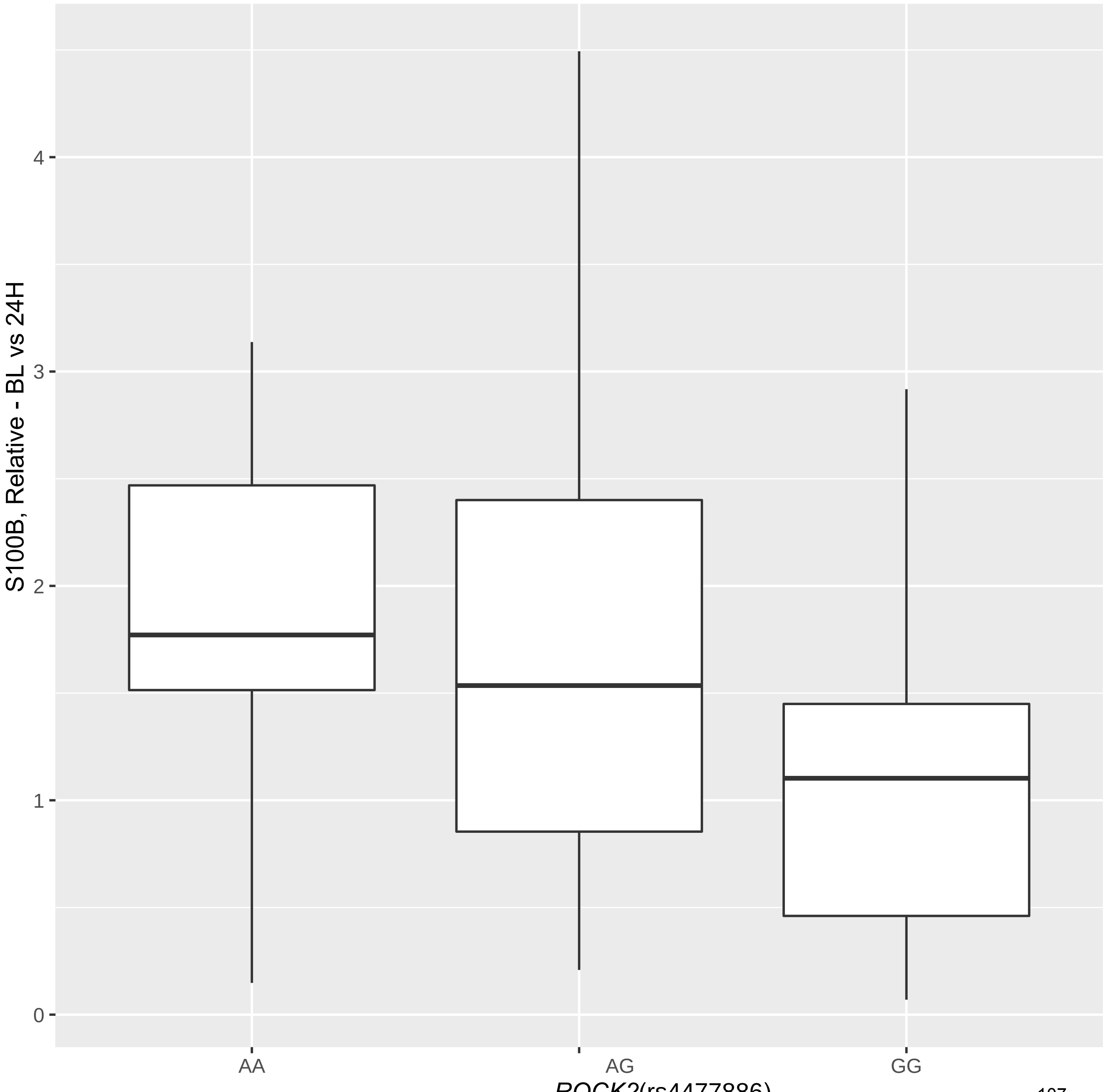
S100B, Relative - BL vs 24H
4
3
2
1
0
AA
AG
GG
ROCK2(rs4477886)

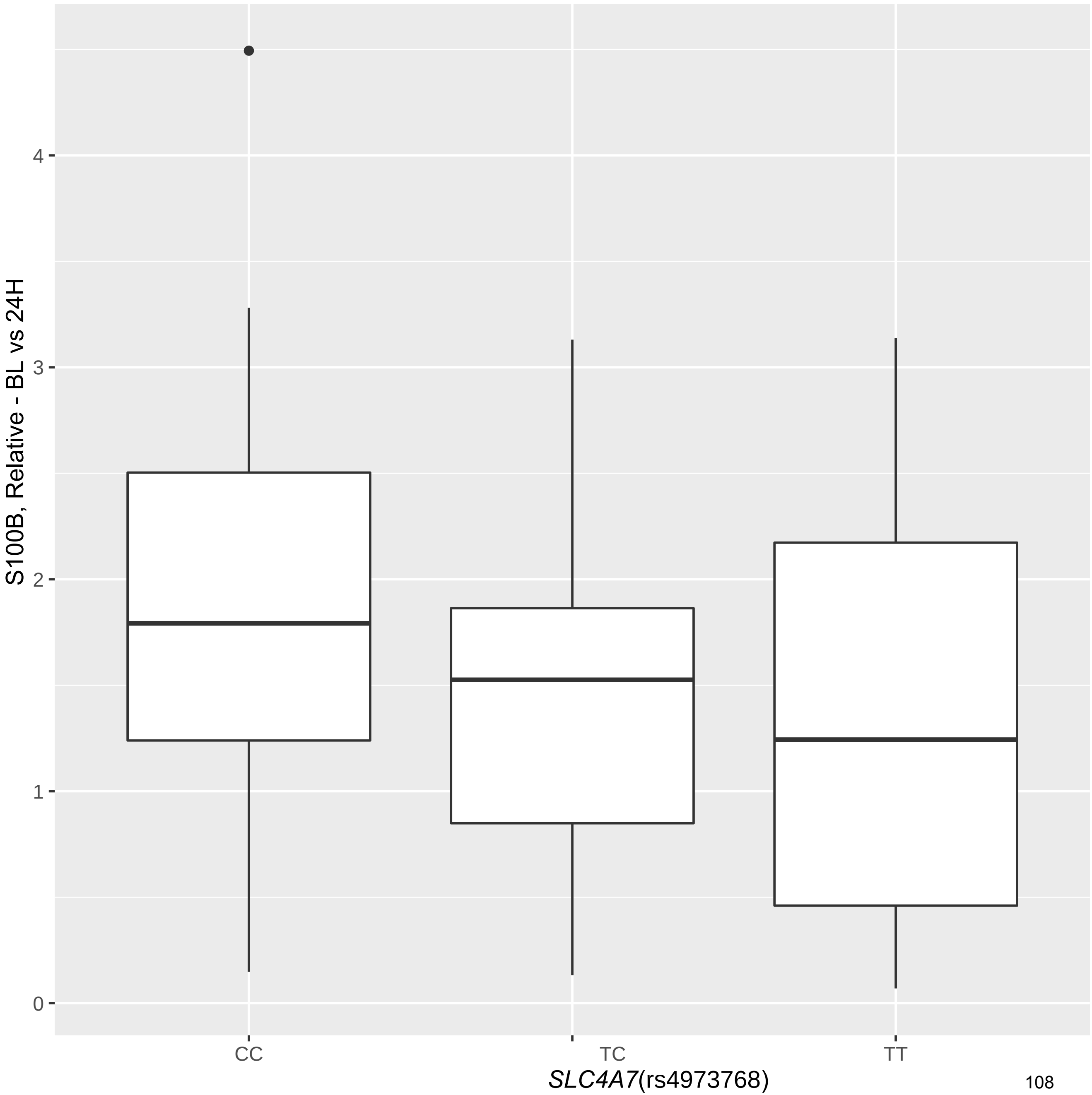

S100B, Relative - BL vs 24H
0
1
2
3
4
CC
TC
TT
SLC4A7(rs4973768)

On Line Supplementary Material References

1. Ruano G, Thompson P, Windemuth A, Smith A, Kocherla M, Holford T, Seip R, Wu A (2005) Physiogenomic analysis links serum creatine kinase activities during statin therapy to vascular smooth muscle homeostasis. Pharmacogeonomics 6(8):865-872
2. Gu D, Su S, Ge D, Chen S, Huang J, Li B, Chen R, Qiang B (2006) Association study with 33 single-nucleotide polymorphisms in 11 candidate genes for hypertension in Chinese. Hypertension 47(6):1147-1154
3. Tempfer C, Riener E, Keck C, Grimm C, Heinze G, Huber J, Gitsch G, Hefler L (2005) Polymorphisms associated with thrombophilia and vascular homeostasis and the timing of menarche and menopause in 728 white women. Menopause 12(3):325-330
4. Zhang J, Jin X, Fang S, Li Y, Wang R, Guo W, Wang N, Wang Y, Wen D, Wei L, Kuang G, Dong Z (2004) The functional SNP in the matrix metalloproteinase-3 promoter modifies susceptibility and lymphatic metastasis in esophageal squamous cell carcinoma but not in gastric cardiac adenocarcinoma. Carcinogenesis 25(12:2519-2524
5. Mogk R, Rothenmund H, Evans J, Carson N, Dawson A (2000) The frequency of the C677T substitution in the methylenetetrahydrofolate reductase gene in Manitoba. [Letter]. Clin Genetics 58(5):406-408
6. Knoblauch H, Bauerfeind A, Toliat MR, Becker C, Luganskaja T, Gunther UP, Rohde K, Schuster H, Junghans C, Luft FC, Nurnberg P, Reich JG (2004) Haplotypes and SNPs in 13 lipid-relevant genes explain most of the genetic variance in high-density lipoprotein and low-density lipoprotein cholesterol. Hum Mol Genet 13 (10):993-1004
7. Heck A, Bray M, Scott A, Blanton S, Hecht J (2005) Variation in CASP10 gene is associated with idiopathic talipes equinovarus. J Ped Orthopedics 25(5):598-602
8. Boersma B, Howe T, Goodman J, Yfantis H, Lee D, Chanock S, Ambs S (2006) Association of breast cancer outcome with status of p53 and MDM2 SNP309. J Nat Cancer Inst 98(13):911-919
9. Harris S, Gil G, Robins H, Hu W, Hirshfield K, Bond E, Bond G, Levine A (2005) Detection of functional single-nucleotide polymorphisms that affect apoptosis. Proceedings of the Natl Acad of Sci of the USA 102(45):16297-16302
10. Prasad P, Tiwari A, Kumar K, Ammini A, Gupta A, Gupta R, Sharma A, Rao A, Nagendra R, Chandra T, Tiwari S, Rastogi P, Gupta B, Thelma B (2006) Chronic renal insufficiency among Asian Indians with type 2 diabetes: I. Role of RAAS gene polymorphisms. BMC Med Genetics 7:42
11. Meng Y, Baldwin C, Bowirrat A, Waraska K, Inzelberg R, Friedland R, Farrer L (2006) Association of polymorphisms in the Angiotensin-converting enzyme gene with Alzheimer disease in an Israeli Arab community. Am J Hum Genet 78(5):871-877
12. del Ser T, Bornstein B, Barba R, Cemillan C (2001) Relationship of angiotensin converting enzyme genotype with serum triglyceride concentration in stroke patients. Neurosci Lett 316 (1):21-24
13. Kostulas K, Huang W, Crisby M, Jin Y, He B, Lannfelt L, Eggertsen G, Kostulas V, Hillert J (1999) An angiotensin-converting enzyme gene polymorphism suggests a genetic distinction between ischaemic stroke and carotid stenosis. Eur J Clin Invest 29 (6):478-483
14. Revilla M, Obach V, Cervera A, Davalos A, Castillo J, Chamorro A (2002) A -174G/C polymorphism of the interleukin-6 gene in patients with lacunar infarction. Neurosci Lett 324 (1):29-32
15. Berti R, Williams A, Moffett J, Hale S, Velarde L, Elliott P, Yao C, Dave J, Tortella F (2002) Quantitative real-time RT-PCR analysis of inflammatory gene expression associated with ischemia-reperfusion brain injury. J Cereb Blood Flow Metab 22(9):1068-1079

16. Abu-Amero K, Al-Boudari O, Mohamed G, Dzimiri N (2006) E-selectin S128R polymorphism and severe coronary artery disease in Arabs. BMC Cardiovas Disorders 7:52
17. Mas V, Fisher R, Maluf D, Archer K, Contos M, Mills S, Shiffman M, Wilkinson D, Oliveros L, Garrett C, Ferreira-Gonzalez A (2004) Polymorphisms in cytokines and growth factor genes and their association with acute rejection and recurrence of hepatitis C virus disease in liver transplantation. Clin Genetics 65(3):191-201
18. Moshynska O, Moshynskyy I, Misra V, Saxena A (2005) G125A single-nucleotide polymorphism in the human BAX promoter affects gene expression. Oncogene 24(12):2042-2049
19. Gomez-Mateo J, Marin L, Lopez-Alvarez MR, Moya-Quiles MR, Miras M, Marin-Moreno I, Botella C, Parrilla P, Alvarez-Lopez MR, Muro M (2006) TGF-beta1 gene polymorphism in liver graft recipients. Transpl Immunol 17 (1):55-57
20. Guo D, Li M, Zhang Y, Yang P, Eckenrode S, Hopkins D, Zheng W, Purohit S, Podolsky R, Muir A, Wang J, Dong Z, Brusko T, Atkinson M, Pozzilli P, Zeidler A, Raffel L, Jacob C, Park Y, Serrano-Rios M, Larrad M, Zhang Z, Garchon H, Bach J, Rotter J, She J, Wang C (2004) A functional variant of SUMO4, a new I kappa B alpha modifier, is associated with type 1 diabetes. Nature Genet 36(8):837-841
21. Jennings C, Owen C, Wilson V, Pearce S (2005) No association of the codon 55 methionine to valine polymorphism in the SUMO4 gene with Graves' disease. Clin Endocrinol 62(3):362-365
22. Topol E, McCarthy J, Gabriel S, Moliterno D, Rogers W, Newby L, Freedman M, Metivier J, Cannata R, O'Donnell C, Kottke-Marchant K, Murugesan G, Plow E, Stenina O, Daley G (2001) Single nucleotide polymorphisms in multiple novel thrombospondin genes may be associated with familial premature myocardial infarction. Circulation 104(22):2641-2644
23. Pluskota E, Stenina O, Krukovets I, Szpak D, Topol E, Plow E (2005) Mechanism and effect of thrombospondin-4 polymorphisms on neutrophil function. Blood 106(12):3970-3978
24. Ahmad-Nejad P, Mrabet-Dahbi S, Breuer K, Klotz M, Werfel T, Herz U, Heeg K, Neumaier M, Renz H (2004) The toll-like receptor 2 R753Q polymorphism defines a subgroup of patients with atopic dermatitis having severe phenotype. J Allergy Clin Immunol 113 (3):565-567
25. Lorenz E, Mira J, Frees K, Schwartz D (2002) Relevance of mutations in the TLR4 receptor in patients with gram-negative septic shock. Arch Intern med 162:1028-1032
26. Merx S, Zimmer W, Neumaier M, Ahmad-Nejad P (2006) Characterization and functional investigation of single nucleotide polymorphisms (SNPs) in the human TLR5 gene. Human Mutation 27 (3):293
27. Zeng S, Yankowitz J, Widness J, Strauss R (2001) Etiology of differences in hematocrit between males and females: sequence-based polymorphisms in erythropoietin and its receptor. J Gender-Specific Med 4(1):35-40
28. Ehrenreich H, Hasselblatt M, Dembowski C, Cepek L, Lewczuk P, Stiefel M, Rustenbeck H, Breiter N, Jacob S, Knerlich F, Bohn M, Poser W, Ruther E, Kochen M, Gefeller O, Gleiter C, Wessel T, De Ryck M, Itri L, Prange H, Cerami A, Brines M, Siren A (2002) Erythropoietin therapy for acute stroke is both safe and beneficial. Molecular Med 8(8):495-505
29. Prass K, Scharff A, Ruscher K, Löwl D, Muselmann C, Victorov I, Kapinya K, Dirnagl U, Meisel A (2003) Hypoxia-Induced Stroke Tolerance in the Mouse Is Mediated by Erythropoietin. Stroke 34:1981-1986
30. Milner C, Campbell R (1992) Polymorphic analysis of the three MHC-linked HSP70 genes. Immunogenetics 36:357-362
31. Schroder O, Schulte K, Ostermann P, Roher H, Ekkernkamp A, RA L (2003) Heat shock protein 70 genotypes HSPA1B and HSPA1L influence cytokine concentrations and interfere with outcome after major injury. Crit Care Med 31(1):73-79

32. Hoy A, Leininger-Muller B, Poirier O, Siest G, Gautier M, Elbaz A, Amarenco P, Visvikis S (2003) Myeloperoxidase polymorphisms in brain infarction. Association with infarct size and functional outcome. Atherosclerosis 167 (2):223-230
33. Ravn-Haren G, Olsen A, Tjonneland A, Dragsted L, Nexo B, Wallin H, Overvad K, Raaschou-Nielsen O, Vogel U (2006) Associations between GPX1 Pro198Leu polymorphism, erythrocyte GPX activity, alcohol consumption and breast cancer risk in a prospective cohort study. Carcinogenesis 27(4):820-825
34. Rosenblum J, Gilula N, Lerner R (1996) On signal sequence polymorphisms and diseases of distribution. Proceedings of the Natl Acad of Sci of the USA 93(9):447-443
35. Ambrosone C, Freudenheim J, Thompson P, Bowman E, Vena J, Marshall J, Graham S, Laughlin R, Nemoto T, Shields P (1999) Manganese superoxide dismutase (MnSOD) genetic polymorphisms, dietary antioxidants, and risk of breast cancer. Cancer Res 59(3):602-606
36. Forsberg L, Lyrenas L, de Faire U, Morgenstern R (2001) A common functional C-T substitution polymorphism in the promoter region of the human catalase gene influences transcription factor binding, reporter gene transcription and is correlated to blood catalase levels. Free Rad Biolog & Med 30(5):500-505
37. Rudofsky GJ, Schroedter A, Schlotterer A, Voron'ko O, Schlimme M, Tafel J, Isermann B, Humpert P, Morcos M, Bierhaus A, Nawroth P, Hamann A (2006) Functional polymorphisms of UCP2 and UCP3 are associated with a reduced prevalence of diabetic neuropathy in patients with type 1 diabetes. Diabetes Care 29(1):89-94
38. Ha E, Choe B-K, Jung K, Yoon S, Park H, Park H, Yim S-V, Chung J-H, Bae H-S, Nam M, Baik H, Hong S-J (2005) Positive relationship between melatonin receptor type 1B polymorphism and rheumatoid factor in rheumatoid arthritis patients in the Korean population. J Pineal Res 39(2):201-205
39. Tan D, Manchester L, Sainz R, Mayo J, Leon J, Reiter R (2005) Physiological ischemia/reperfusion phenomena and their relation to endogenous melatonin production: a hypothesis. Endocrine 27 (2):149-158
40. Bremer T, Man A, Kask K, Diamond C (2006) CACNA1C polymorphisms are associated with the efficacy of calcium channel blockers in the treatment of hypertension. Pharmacogeonomics 7(3):271-279
41. Kokubo Y, Inamoto N, Tomoike H, Kamide K, Takiuchi S, Kawano Y, Tanaka C, Katanosaka Y, Wakabayashi S, Shigekawa M, Hishikawa O, Miyata T (2004) Association of genetic polymorphisms of sodium-calcium exchanger 1 gene, NCX1, with hypertension in a Japanese general population. Hypertension Research - Clin & Exper 27(10):697-702
42. Honorio S, Gordon K, MacCartney D, Agathanggelou A, Latif F (2001) Identification of a single nucleotide polymorphism in the human alpha 2 delta 2 calcium channel subunit gene. Molecular & Cellular Probes 15(6):391-393
43. Angeloni D, Wei M, Duh F, Johnson B, Lerman M, Angeloni D, Wei M, Duh F, Johnson B, Lerman M (2000) G-to-A single nucleotide polymorphism in the human alpha 2 delta 2 calcium channel subunit gene that maps at chromosome 3p21.3. Molecular & Cellular Probes 14(1):53-54
44. Nakano T, Osanai T, Tomita H, Sekimata M, Homma Y, Okumura K (2002) Enhanced activity of variant phospholipase C-delta1 protein (R257H) detected in patients with coronary artery spasm. Circulation 105(17):2024-2029
45. Muralikrishna Adibhatla R, Hatcher J (2006) Phospholipase A2, reactive oxygen species, and lipid peroxidation in cerebral ischemia. Free Rad Biolog & Med 40(3):376-387
46. Milting H, Lukas N, Klauke B, Korfer R, Perrot A, Osterziel K, Vogt J, Peters S, Thieleczek R, Varsanyi M (2006) Composite polymorphisms in the ryanodine receptor 2 gene associated with arrhythmogenic right ventricular cardiomyopathy. Cardiovascular Research 71 (3):496-505

47. Mototani H, Mabuchi A, Saito S, Fujioka M, Iida A, Takatori Y, Kotani A, Kubo T, Nakamura K, Sekine A, Murakami Y, Tsunoda T, Notoya K, Nakamura Y, Ikegawa S (2005) A functional single nucleotide polymorphism in the core promoter region of CALM1 is associated with hip osteoarthritis in Japanese. Human Molecular Genetics 14(8):1009-1017
48. Seasholtz T, Wessel J, Rao F, Rana B, Khandrika S, Kennedy B, Lillie E, Ziegler M, Smith D, Schork N, Brown J, O'Connor D (2006) Rho kinase polymorphism influences blood pressure and systemic vascular resistance in human twins: role of heredity. Hypertension 47(5):937-947
49. Wiggins A, Shen P, Gundlach A (2003) Atrial natriuretic peptide expression is increased in rat cerebral cortex following spreading depression: possible contribution to sd-induced neuroprotection. Neurosci 118(3):715-726
50. Rubattu S, Stanzione R, Di Angelantonio E, Zanda B, Evangelista A, Tarasi D, Gigante B, Pirisi A, Brunetti E, Volpe M (2004) Atrial natriuretic peptide gene polymorphisms and risk of ischemic stroke in humans. Stroke 35(4):814-818
51. Lindstrom S, Wiklund F, Jonsson B, Adami H, Balter K, Brookes A, Xu J, Zheng S, Isaacs W, Adolfsson J, Gronberg H (2005) Comprehensive genetic evaluation of common E-cadherin sequence variants and prostate cancer risk: strong confirmation of functional promoter SNP. Human Genetics 118(3-4):339-347
52. Niemi A, Moilanen J, Tanaka M, Hervonen A, Hurme M, Lehtimaki T, Arai Y, Hirose N, Majamaa K (2005) A combination of three common inherited mitochondrial DNA polymorphisms promotes longevity in Finnish and Japanese subjects. Eur J Human Genetics 13(2):166-170
53. Fallucca F, Dalfra M, Sciullo E, Masin M, Buongiorno A, Napoli A, Fedele D, Lapolla A (2006) Polymorphisms of insulin receptor substrate 1 and beta3-adrenergic receptor genes in gestational diabetes and normal pregnancy. Metabolism: Clinical & Experimental 55 (11):1451-1456
54. Hodgkinson A, Page T, Millward B, Demaine A (2005) A novel polymorphism in the 5' flanking region of the glucose transporter (GLUT1) gene is strongly associated with diabetic nephropathy in patients with Type 1 diabetes mellitus. J Diabetes & Its Complicaitons 19(2):65-69
55. Benarroch E (2005) Neuron-astrocyte interactions: partnership for normal function and disease in the central nervous system. Mayo Clin Proc 80(10):1326-1338
56. Carlsson E, Fredriksson J, Groop L, Ridderstrale M (2004) Variation in the calpain-10 gene is associated with elevated triglyceride levels and reduced adipose tissue messenger ribonucleic acid expression in obese Swedish subjects. J Clin Endocrinology & Metab 89(7):3601-3605
57. Heck AL, Bray MS, Scott A, Blanton SH, Hecht JT (2005) Variation in CASP10 gene is associated with idiopathic talipes equinovarus. J Pediatr Orthop 25 (5):598-602
58. MacPherson G, Healey CS, Teare MD, Balasubramanian SP, Reed MW, Pharoah PD, Ponder BA, Meuth M, Bhattacharyya NP, Cox A (2004) Association of a common variant of the CASP8 gene with reduced risk of breast cancer. J Natl Cancer Inst 96 (24):1866-1869
59. Shima Y, Nakanishi K, Odawara M, Kobayashi T, Ohta H (2003) Association of the SNP-19 genotype 22 in the calpain-10 gene with elevated body mass index and hemoglobin A1c levels in Japanese. Clinica Chimica Acta 336(1-2):89-96
60. Magri C, Gardella R, Barlati S, Podavini D, Iatropoulos P, Bonomi S, Valsecchi P, Sacchetti E, Barlati S (2006) Glutamate AMPA receptor subunit 1 gene (GRIA1) and DSM-IV-TR schizophrenia: a pilot case-control association study in an Italian sample. Am J Med Genetics 141(3):287-293
61. Marenco S, Steele S, Egan M, Goldberg T, Straub R, Sharrief A, Weinberger D (2006) Effect of metabotropic glutamate receptor 3 genotype on N-acetylaspartate measures in the dorsolateral prefrontal cortex. Am J Psych 163(4):740-742
62. Shuang M, Liu J, Jia M, Yang J, Wu S, Gong X, Ling Y, Ruan Y, Yang X, Zhang D (2004) Family-based association study between autism and glutamate receptor 6 gene in Chinese Han trios. J Med Genetics 131(1):48-50

63. Deng X, Shibata H, Ninomiya H, Tashiro N, Iwata N, Ozaki N, Fukumaki Y (2004) Association study of polymorphisms in the excitatory amino acid transporter 2 gene (SLC1A2) with schizophrenia. BMC Psychiatry 4:21
64. Hu X, Malik S, Negroiu CC, Hubbard K, Velalar CN, Hampton B, Grosu D, Catalano J, Roeder RG, Gnatt A (2006) A Mediator-responsive form of metazoan RNA polymerase II. Proceedings of the National Academy of Sciences of the United States of America 103 (25):9506-9511
65. Guinn BA, Bland EA, Lodi U, Liggins AP, Tobal K, Petters S, Wells JW, Banham AH, Mufti GJ (2005) Humoral detection of leukaemia-associated antigens in presentation acute myeloid leukaemia. Biochem Biophys Res Commun 335 (4):1293-1304
66. Roginski RS, Mohan Raj BK, Birditt B, Rowen L (2004) The human GRINL1A gene defines a complex transcription unit, an unusual form of gene organization in eukaryotes. Genomics 84 (2):265-276
67. Hoehe M, Kopke K, Wendel B, Rohde K, Flachmeier C, Kidd K, Berrettini W, Church G (2000) Sequence variability and candidate gene analysis in complex disease: association of mu opioid receptor gene variation with substance dependence. Human Molecular Genetics 9(19):2895-2908
68. Lotsch J, Skarke C, Wieting J, Oertel B, Schmidt H, Brockmoller J, Geisslinger G (2006) Modulation of the central nervous effects of levomethadone by genetic polymorphisms potentially affecting its metabolism, distribution, and drug action. Clin Pharm & Therap 79(1):72-89
69. Bergen A, van den Bree M, Yeager M, Welch R, Ganjei J, Haque K, Bacanu S, Berrettini W, Grice D, Goldman D, Bulik C, Klump K, Fichter M, Halmi K, Kaplan A, Strober M, Treasure J, Woodside B, Kaye W (2003) Candidate genes for anorexia nervosa in the 1p33-36 linkage region: serotonin 1D and delta opioid receptor loci exhibit significant association to anorexia nervosa. Molecular Psychiatry 8(4):397-406
70. Kinirons P, Cavalleri G, Shahwan A, Wood N, Goldstein D, Sisodiya S, Delanty N, Doherty C (2006) Examining the role of common genetic variation in the gamma2 subunit of the GABA(A) receptor in epilepsy using tagging SNPs. Epilepsy Research 70 (2-3):229-238
71. Schwab S, Franke P, Hoefgen B, Guttenthaler V, Lichtermann D, Trixler M, Knapp M, Maier W, Wildenauer D (2005) Association of DNA polymorphisms in the synaptic vesicular amine transporter gene (SLC18A2) with alcohol and nicotine dependence. Neuropsychopharmacology 30 (12):2263-2268
72. Telleria J, Blanco-Quiros A, Muntion S, Garrote JA, Arranz E, Armentia A, Diez I, Castro J (2006) Tachyphylaxis to beta2-agonists in Spanish asthmatic patients could be modulated by beta2-adrenoceptor gene polymorphisms. Respiratory Medicine 100 (6):1072-1078
73. Masuo K, Katsuya T, Kawaguchi H, Fu Y, Rakugi H, Ogihara T, Tuck M (2006) Beta2-adrenoceptor polymorphisms relate to obesity through blunted leptin-mediated sympathetic activation. American Journal of Hypertension 19 (10):1084-1091
74. Papazoglou D, Papanas N, Papatheodorou K, Kotsiou S, Christakidis D, Maltezos E (2006) An insertion/deletion polymorphism in the alpha2B adrenoceptor gene is associated with age at onset of type 2 diabetes mellitus. Experimental & Clinical Endocrinology & Diabetes 114 (8):424-427
75. Yabe M, Matsubara Y, Takahashi S, Ishihara H, Shibano T, Miyaki K, Omae K, Watanabe G, Murata M, Ikeda Y (2006) Identification of ADRA2A polymorphisms related to shear-mediated platelet function. Biochemical & Biophysical Research Communications 347 (4):1001-1005
76. Neumeister A, Drevets W, Belfer I, Luckenbaugh D, Henry S, Bonne B, Herscovitch P, Goldman D, Charney D (2006) Effects of a alpha 2C-adrenoreceptor gene polymorphism on neural responses to facial expressions in depression. Neuropsychopharmacology 31 (8):1750-1756
77. Hong C, Wang Y, Liu T, Liu H, Tsai S (2001) A study of alpha-adrenoceptor gene polymorphisms and Alzheimer disease. Journal of Neural Transmission 108 (4):445-450
78. Nieminen T, Lehtimaki T, Laiho J, Rontu R, Niemela K, Koobi T, Lehtinen R, Viik J, Turjanmaa V, Kahonen M (2006) Effects of polymorphisms in beta1-adrenoceptor and alpha-subunit of G protein on heart rate and blood pressure during exercise test. The Finnish Cardiovascular Study 100 (2):507-511

79. Hunt S, Xin Y, Wu L, Cawthon R, Coon H, Hasstedt S, Hopkins P (2006) Sodium bicarbonate cotransporter polymorphisms are associated with baseline and 10-year follow-up blood pressures. Hypertension 47(3):532-536
80. Lenzen K, Heils A, Lorenz S, Hempelmann A, Hofels S, Lohoff F, Schmitz B, Sander T (2005) Supportive evidence for an allelic association of the human KCNJ10 potassium channel gene with idiopathic generalized epilepsy. Epilepsy Res 63(2-3):113-118
81. Long J, Xu H, Zhao L, Liu P, Shen H, Liu Y, Xiong D, Xiao P, Liu Y, Dvornyk V, Li J, Recker R, Deng H (2005) The oestrogen receptor alpha gene is linked and/or associated with age of menarche in different ethnic groups. J Med Genetics 42(10):796-800
82. Hakansson A, Westberg L, Nilsson S, Buervenich S, Carmine A, Holmberg B, Sydow O, Olson L, Johnels B, Eriksson E, Nissbrandt H (2005) Interaction of polymorphisms in the genes encoding interleukin-6 and estrogen receptor beta on the susceptibility to Parkinson's disease. Am J Med Genetics 133(1):88-92
83. Westberg L, Hakansson A, Melke J, Shahabi H, Nilsson S, Buervenich S, Carmine A, Ahlberg J, Grundell M, Schulhof B, Klingborg K, Holmberg B, Sydow O, Olson L, Johnels E, Eriksson E, Nissbrandt H (2004) Association between the estrogen receptor beta gene and age of onset of Parkinson's disease. Psychoneuroendocrinology 29(8):993-998
84. Akil H (Summer 2003) Scientific Strategy in Neuroscience: Discovery Science versus Hypothesis-Driven Research. Society for Neurscience Newsletter ( )
85. Ito D, Tanahashi N, Murata M, Sato H, Saito I, Watanabe K, Fukuuchi Y (2002) Notch3 gene polymorphism and ischaemic cerebrovascular disease. J Neuro, Neurosurg Psychiat 72(3):382-384
